# A coordinate-based meta-analysis of human amygdala connectivity alterations related to early life adversities

**DOI:** 10.1038/s41598-023-43057-2

**Published:** 2023-10-02

**Authors:** Eline J. Kraaijenvanger, Tobias Banaschewski, Simon B. Eickhoff, Nathalie E. Holz

**Affiliations:** 1 Department of Child and Adolescent Psychiatry and Psychotherapy, Central Institute of Mental Health, Medical Faculty Mannheim/Heidelberg University, J5, 68159 Mannheim, Germany; 2 Institute of Systems Neuroscience, Medical Faculty, Heinrich Heine University Düsseldorf, Düsseldorf, Germany; 3 Institute of Neuroscience and Medicine, Brain & Behaviour (INM-7), Research Centre Jülich, Jülich, Germany; 4 Donders Institute, Radboud University, Nijmegen, The Netherlands; 5 Radboud University Medical Centre, Nijmegen, The Netherlands

**Keywords:** Emotion, Stress and resilience, Risk factors

## Abstract

By affecting core neurobiological systems early in development, early life adversities (ELAs) might confer latent vulnerability to future psychopathologies. This coordinate-based meta-analysis aims to identify significant convergent alterations in functional connectivity of the amygdala related to ELAs across resting-state and task-based fMRI-studies. Five electronic databases were systematically searched until 22 October 2020, retrieving 49 eligible studies (*n* = 3162 participants). Convergent alterations in functional connectivity related to ELAs between the amygdala and the anterior cingulate cortex (ACC) and left hippocampus were found. Sub-analyses based on hemisphere and direction showed that connectivity seeded in the right amygdala was affected and, moreover, revealed that connectivity with ACC was decreased. Analyses based on paradigm and age showed that amygdala-ACC coupling was altered during resting state and that amygdala–left hippocampus connectivity was mostly affected during task-based paradigms and in adult participants. While both regions showed altered connectivity during emotion processing and following adverse social postnatal experiences such as maltreatment, amygdala-ACC coupling was mainly affected when ELAs were retrospectively assessed through self-report. We show that ELAs are associated with altered functional connectivity of the amygdala with the ACC and hippocampus. As such, ELAs may embed latent vulnerability to future psychopathologies by systematically affecting important neurocognitive systems.

## Introduction

The potential impact of unfavourable environmental conditions during childhood is well established across literature. By affecting core neural networks for threat, stress and autobiographical memory processing early in development, exposure to such early life adversities (ELAs) may shape one’s vulnerability to future psychopathologies^1–5^. After all, these neural recalibrations might serve to cope with the negative environments during childhood, but may become maladaptive under certain conditions later in life^2,6^. Both human and animal research into the neurobiological correlates of ELAs has highlighted the amygdala as key convergence site^3,4,7,8^. In general, amygdala activity supports emotion processing and salience detection, particularly stimuli that are associated with danger^9,10^. As these processes critically rely on continuous interactions between the amygdala and other sensory and regulatory brain regions, ELAs may impact these dynamic networks as well. Indeed, extensive evidence has linked alterations in amygdala-prefrontal circuits, playing a central role in integrating information and regulating emotional responses^11–14^, to ELAs^3–5,15–20^—ranging from prenatal stress exposure to childhood maltreatment. Similarly, altered connections between the amygdala and the hippocampus, synergistically involved in stress responsiveness and emotional memory consolidation^21–25^, have also been reported in relation to ELAs^3,26–30^. As changes in these important circuits have been observed across stress-related psychopathologies (e.g. Refs.^22,31–33^), this may suggest a mediating role of these circuits in conferring risk for pluripotent transdiagnostic trajectories in relation to ELAs.

Alterations in amygdala connectivity in participants with ELAs have been highly heterogeneous. This pertains not only to the direction of amygdala coupling^3^ but also to the target regions^20^. Likewise, original studies often prioritized investigating the effects on amygdala coupling to specific regions of interest and thereby potentially overlook broader effects on a whole-brain level (e.g. Ref.^3^). In this study, we therefore conducted a quantitative summary of individual findings on a whole-brain level to capture these heterogeneous findings. By pooling data from multiple studies and analysing coordinates of altered connectivity, we aimed to comprehensively evaluate the variability and convergence of findings across different regions to identify consistent patterns of affected amygdala connectivity in relation to ELAs. Following current recommendations for coordinate-based meta-analyses (CBMAs)^34–36^, this study is the first to decipher the overall effect of ELAs on amygdala network connectivity. In this context, we consider ELAs more broadly as developmental risk factors acting early in life and therefore included both prenatal exposures (e.g. substance exposure) and postnatal experiences (e.g. childhood maltreatment or poverty). In line with our previous meta-analyses on neural alterations related to ELAs^4,5^, we performed a coordinate-based meta-analysis using activation likelihood estimation (ALE; for an explanation, see Ref.^37^)^36,38,39^ to consolidate this yet inconclusive literature and assess robust effects of altered amygdala connectivity across samples and analytic approaches. Based on the seed-based amygdala connectivity literature (for a recent review, see Ref.^3^), we hypothesize that particularly the amygdala-PFC circuit and amygdala-hippocampus circuit are affected in relation to ELAs. As such, these functional connectivity phenotypes might serve as stratification markers for early detection and treatment of the potentially long-lasting effects of ELAs throughout life.

## Results

**Figure 1:**
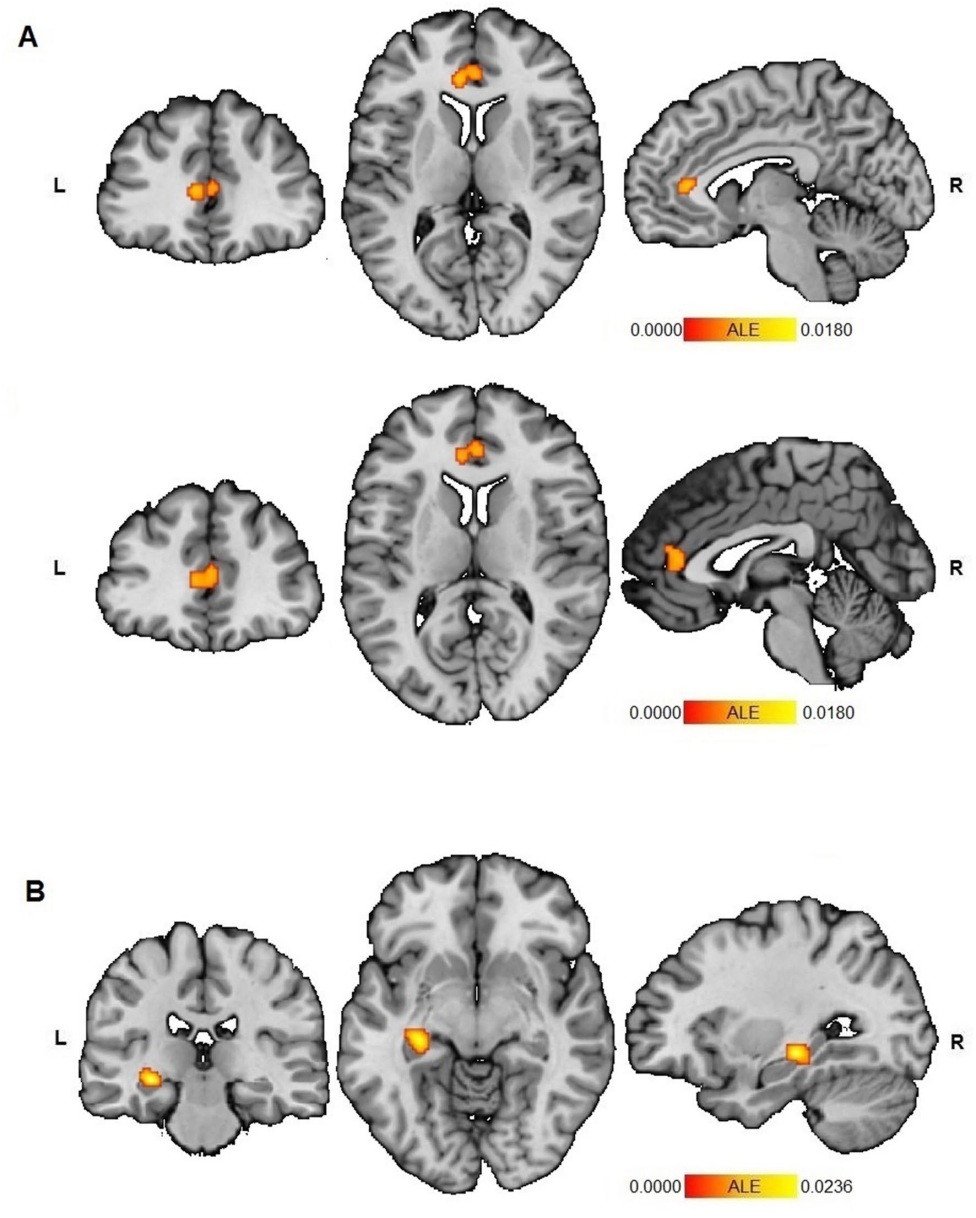
ALE analyses for functional alteration in connectivity with the amygdala as seed-region revealed two significant clusters of convergence, with the first within the **(A)** ACC (BA24), with peaks at [− 6, 36, 8], ALE = 0.0180 and at [2, 38, 10], ALE = 0.0167, and the second cluster within the **(B)** left hippocampus ([− 30, − 26, − 10], ALE = 0.0236). The ALE map was computed in MNI152 and thresholded (*p*_cluster-level_ < 0.05, *p*_uncorrected_ < 0.001). L = left, R = right. ALE analyses were conducted using GingerALE 3.0.2 (https://www.brainmap.org/ale/) and results were visualized using Mango 4.1 (https://mangoviewer.com/).

The global ALE analysis on the overall effect of ELAs was based on 45 experiments (3162 participants) and revealed convergence within the anterior cingulate cortex (ACC; L ACC: ALE-value: 0.0180, Z-score: 3.99; R ACC: ALE-value: 0.0167, Z-score: 3.78) (Fig. 1A, Table 1) and the left hippocampus (ALE-value: 0.0236, Z-score: 4.76) (Fig. 1B, Table 1).

**Table 1:** Results of the individual ALE analyses.

ALE analysis	*N*	#Experiments	#Foci	Cluster	Volume (mm^3^)	Brain region	*X*	*Y*	*Z*	ALE value	Z score	Studies contributing to cluster
All studies	3162	45	272	2	1072 904	L ACC R ACC L hippocampus	− 6 2 − 30	36 38 − 26	8 10 − 10	0.0180 0.0167 0.0236	3.99 3.78 4.76	Cisler, 2017; Fan, 2014; Kraynak, 2020; Lee, 2015; Radhakrishnan, 2020; Thomason, 2015 Holz, 2015; Jedd, 2015; Maier 2020; Radhakrishnan, 2020; van der Werff, 2013a; van der Werff, 2013b
Resting-state	1725	25	164	1	960	L/R ACC	2	38	10	0.0163	4.16	Cisler, 2017; Fan, 2014; Kraynak, 2020; Radhakrishnan, 2020; Thomason, 2015
Task-based	1601	22	109	1	672	L hippocampus	− 30	− 26	− 10	0.0204	4.92	Holz, 2015; Jedd, 2015; Maier 2020
Emotion processing	1569	21	98	2	704 488	L hippocampus L ACC	− 30 − 2	− 26 42	− 10 − 16	0.0204 0.0125	4.97 3.69	Holz, 2015; Jedd, 2015; Maier 2020 Fonzo, 2013; Hanford, 2019; Maier 2020; Peverill, 2019
Decrease in connectivity	2175	30	131	1	1344	R ACC L ACC	2 − 6	42 38	14 6	0.0146 0.0138	3.87 3.76	Cisler, 2017; Fan, 2014; Kraynak, 2020; Lee, 2015; Radhakrishnan, 2020; Thomason, 2015; Wolf, 2016
Increase in connectivity	2065	32	142	–								
Right hemisphere	2099	31	151	2	832 784	L hippocampus L ACC R ACC	− 32 − 6 2	− 24 36 42	− 8 8 14	0.0214 0.0174 0.0140	4.93 4.34 3.74	Jedd, 2015; Maier 2020; Radhakrishnan, 2020; van der Werff, 2013a; van der Werff, 2013b Fan, 2014; Kraynak, 2020; Lee, 2015; Radhakrishnan, 2020; Thomason, 2015
Left hemisphere	2592	35	135	–								
Adults	1683	21	171	1	808	L hippocampus	− 30	− 26	− 10	0.023	5.11	Holz, 2015; Jedd, 2015; Maier 2020; van der Werff, 2013a; van der Werff, 2013b
Children	1479	24	101	1	1136	L ACC	− 6	34	8	0.0155	4.18	Cisler, 2017; Lee, 2015; Radhakrishnan et al., 2020; Thomason et al., 2015
Healthy	2207	29	197	2	776 752	L ACC L hippocampus	− 6 − 2 − 30	36 38 − 26	8 10 − 10	0.0174 0.0153 0.0209	4.12 3.76 4.65	Fan, 2014; Kraynak, 2020; Lee, 2015; Radhakrishnan, 2020; Thomason, 2015 Holz, 2015; Jedd, 2015; Maier 2020
Patients	955	16*	75	Not enough studies available								
Social ELAs	2527	33	229	–								
Social ELAs, adults	1642	20	168	1	824	L hippocampus	− 30	− 26	− 8	0.0231	5.09	Holz, 2015; Jedd, 2015; Maier 2020; van der Werff, 2013a; van der Werff, 2013b
Social ELAs, children	723	14*	60	Not enough studies available								
Social ELAs, decrease	1669	21	112	1	1216	L/R ACC	2	42	14	0.0142	3.9	Cisler, 2017; Fan, 2014; Kraynak, 2020; Lee, 2015; Thomason, 2015
Social ELAs, increase	1511	22	116	–								
Social ELAs, right hemisphere	1517	21	131	–								
Social ELAs, left hemisphere	1911	23	139	–								
Socioeconomic ELAs	381	6*	17	Not enough studies available								
Postnatal ELAs	2946	40	250	–								
Postnatal ELAs, decrease	2063	27	127	1	1368	L/R ACC	2	42	14	0.0146	3.89	Cisler, 2017; Fan, 2014; Kraynak, 2020; Lee, 2015; Thomason, 2015, Wolf, 2016
Postnatal ELAs, increase	1807	28	126	–								
Postnatal ELAs, right amygdala	1982	29	186	–								
Postnatal ELAs, rest	1509	20	142	–								
Postnatal ELAs, emotion	1437	20	97	2	704	L hippocampus L ACC	− 30 − 2	− 26 42	− 10 − 16	0.0204 0.0125	4.99 3.7	Holz, 2015; Jedd, 2015; Maier 2020 Fonzo, 2013; Hanford, 2019; Maier 2020; Peverill, 2019
Postnatal ELAs, adults	1683	21	171	1	808	L hippocampus	− 30	− 26	− 10	0.023	5.11	Holz, 2015; Jedd, 2015; Maier 2020; van der Werff, 2013a; van der Werff, 2013b
Postnatal ELAs, children	1263	19	79	–								
Prenatal ELAs	335	6*	22	Not enough studies available								
Retrospective assessment	2061	31	216	1	752	L ACC	2	42	14	0.0146	3.62	Cisler, 2017; Fan, 2014; Kraynak, 2020; Lee, 2015; Thomason, 2015
Prospective assessment	1101	14*	56	Not enough studies available								
Subjective self-report by participants	1861	26	208	1	800	L ACC	2	42	14	0.0146	3.66	Cisler, 2017; Fan, 2014; Kraynak, 2020; Lee, 2015; Thomason, 2015
Objective assessment	1101	13*	56	Not enough studies available								

L = left, R = right; *X, Y, Z* coordinates in MNI152 space. ACC, anterior cingulate cortex; ALE, activation likelihood estimation; ELAs, early life adversities.

*These analyses could not be performed due to an insufficient number of experiments based on current CBMA guidelines (≥ 17 experiments)^34–36^.

Additional sub-analyses were performed if a sufficient number of experiments (*n* ≥ 17) was available^35^. Separating the analyses by paradigm (resting-state or task-based) indicated convergent altered coupling of the amygdala with the ACC during resting-state (25 experiments, *n* = 1725; ALE-value: 0.0163, Z-score: 4.16) and with the left hippocampus during task-based paradigms (22 experiments; *n* = 1601; ALE-value: 0.0204; Z-score: 4.92) in relation to ELAs. Both regions showed altered connectivity in the context of emotion processing as well (21 experiments, *n* = 1569; L hippocampus: ALE-value: 0.0204, Z-score: 4.97; L ACC: ALE-value: 0.0125, Z-score: 3.69). Analyses based on direction of altered connectivity (increase or decrease) revealed significant convergence within the ACC (30 experiments, *n* = 2175; L ACC: ALE-value: 0.0146, Z-score: 3.87; R ACC: ALE-value: 0.0138, Z-score: 3.76) reflecting a consistent decrease in connectivity in relation to ELAs, while no cluster for increase was found (32 experiments, *n* = 2065). Separating by hemisphere revealed convergence within the left hippocampus related with ELAs (ALE-value: 0.0214, Z-score: 4.93) and within the ACC (L ACC: ALE-value: 0.0174, Z-score: 4.34; R ACC: ALE-value: 0.0140, Z-score: 3.74) for connectivity analyses seeded in the right amygdala (31 experiments, *n* = 2175), but no cluster appeared for the left amygdala (35 experiments, *n* = 2592). Subdividing the experiments by age (children or adults), revealed convergence within the left hippocampus for adults (21 experiments, *n* = 1683; ALE-value: 0.023, Z-score: 5.11) and within the left ACC for children (24 experiments, *n* = 1479; ALE-value: 0.0155; Z-score: 4.18). Only including experiments with healthy participants (29 experiments, *n* = 2207), to ensure no confounding effects of psychopathology, corroborated two clusters within the left ACC (ALE-value: 0.0174 and 0.0153, Z-score: 4.12 and 3.76) and one within the left hippocampus (ALE-value: 0.0209, Z-score: 4.65).

Separate analyses per ELA-subtype revealed convergence in the left hippocampus for postnatal adverse social experiences such as maltreatment and trauma in adults (20 experiments, *n* = 1642; ALE-value: 0.0231, Z-score: 5.09), which also resulted in convergently decreased connectivity with the ACC in relation to ELAs (21 experiments, *n* = 1669; ALE-value: 0.0142, Z-score: 3.90). No additional convergence was revealed for further sub-specifications of these ELAs. Lastly, differentiating based on the type of assessment indicated that amygdala-ACC connectivity was altered in relation to ELAs in studies relying on subjective self-reports (26 experiments, *n* = 1861; ACE-value: 0.0146, Z-score: 3.66) and in participants that were assessed retrospectively (31 experiments, *n* = 2061; ACE-value: 0.0146, Z-score: 3.62).

Functional decoding confirmed the involvement of these clusters during positive and negative emotion processing, as well as higher cognitive functions (see supplement and Fig. S1).

## Discussion

Our meta-analysis provides a comprehensive overview of neuroimaging studies investigating the association of ELAs with brain connectivity using the amygdala as seed-region. We have demonstrated robust evidence for decreased amygdala-ACC and altered amygdala-hippocampus connectivity in connection with ELAs, that support a certain level of equifinality of ELAs in these neural adaptations^40^. As such, this study complements previous meta-analyses reporting neural alterations associated with ELAs^4,5,41^ and offers more insight into the mechanisms of how ELAs might become embedded into the human brain.

The overall analysis revealed significant alterations in functional connectivity related to ELAs between the amygdala and the ACC, as well as between the amygdala and the left hippocampus—which is in line with previous studies^3–5,18,42–44^. Subsequent sub-analyses were applied to further specify these results by restricting them based on paradigm, direction, hemisphere, age, disease status, ELA-subtype or -assessment.

For the ACC, these sub-analyses revealed a predominant decrease in amygdala-ACC connectivity mainly arising from the right amygdala during resting-state related to postnatal, social ELAs in healthy participants—especially when these ELAs were retrospectively assessed through self-report. This has several implications. First, as the ACC can be recruited to inhibit negative emotional processing in the amygdala^14^, decreased connectivity between the amygdala and ACC might indicate that the effectivity of this emotional stress resolution is affected in relation to ELAs—which may result in altered stress reactivity^22,45–47^ and memory extinction^48^. Second, as the right amygdala is mainly responsible for global, dynamic detection of (negative) emotional stimuli due to a faster habituation rate^49–52^, this further suggests that ELAs are related to altered automatic, emotional stress responses, which may lead to a more sustained emotional responding^49,53^. This aspect is corroborated by the fact that altered amygdala-ACC connectivity associated with ELAs primarily arises during the resting-state paradigm, a measure of intrinsic brain connectivity^29^. Overall, the altered resting-state connectivity pattern seems to be consistent across developmental stages, which is in line with previous studies^23^. Moreover, alterations in functional connectivity between the amygdala and ACC, as well as the hippocampus for that matter, are particularly associated with postnatal social ELAs, such as maltreatment. This intuitively makes sense: severe and prolonged trauma experiences can have severe (neural) consequences, and are correlated in space and time—a line of reasoning that is supported by literature^3,54^. It must however be taken into account that most of the studies investigated postnatal social ELAs and that the planned analysis for either socio-economic ELAs or prenatal exposures could not be performed due to an insufficient number of experiments. Lastly, the observation of altered amygdala-ACC connectivity primarily in individuals who self-report ELAs, might reinforce the hypothesis that such alterations contribute to enhanced cognitive biases that intensify the subjective evaluation of ELAs. This complements recent findings on different risk pathways for prospective and retrospective assessments^55^, and a superiority of the impact of subjectively experienced ELA-burden on the development of psychopathology^56^.

For the hippocampus, the sub-analyses further specified the results to an altered connectivity between right amygdala–left hippocampus. This alteration was predominantly observed in task-based experiments involving healthy adults who experienced postnatal, social ELAs. Interestingly, contributors to the task-based effect mainly employed an emotion processing paradigm, which further implicates this link between amygdala and hippocampus in emotional memory processing^21^. It also raises the assumption that ELAs might foster vulnerability to future psychopathologies via increased emotional memory consolidation^21,22^. The observation that this altered connectivity in relation to ELAs mainly arises in adult participants parallels previous literature showing hippocampal alterations in adults, but not in children^57^. Given the positive correlation between ELAs and future stress throughout life^58^, it might be that this cumulative effect of stress only manifests itself in adulthood—probably mediated by chronic stress-induced hippocampal glucocorticoid exposure^33,47,59–61^.

Multiple theoretical frameworks have tried to capture the range of relationships between ELAs and neural adaptations. These include the latent vulnerability framework^2^, implicating that changes in neurocognitive systems in relation to ELAs, reflecting an adaptation to these negative early environments, alter one’s vulnerability to future mental health problems. In later life, exposure to stress or challenge might unveil these vulnerabilities, thereby manifesting as clinical symptoms. Furthermore, the allostatic load model^56^ suggests that intense and enduring exposure to adversities can disrupt the body's ability to maintain homeostasis, resulting in a dysregulation of the stress response^56^. In addition, the cumulative stress model^62^ emphasizes the accumulation of stressors over time, implying that the combined effect of multiple stressors can have a significant impact on health outcomes. Overall, these models are not mutually exclusive in conceptualizing the complex and currently incompletely understood nature and consequences of ELAs, and instead might influence and complement each other. For example, chronic stress exposure during childhood can alter the allostatic load of physiological systems, such as amygdala-hippocampal coupling. These changes may act as latent vulnerabilities, thereby modifying responses to future stressors^2^ and in turn impacting the allostatic load even more. Another conceptualization is the dimensional model of adversity, implicating that different stressors, such as threat- and deprivation-related ELAs, might act on qualitatively different mechanisms to increase the risk for specific psychopathologies^20,63–65^. While intriguing, this framework remains to be elucidated by future meta-analyses, given that most of the included studies encompass multi-faceted adversities with only few studies making use of this suggested dimensional approach^63^. Importantly, in order to establish a clear, coherent and consistent model to conceptualize the neural adaptations in relation to ELAs in its entirety, the understanding of the exact mechanisms by which ELAs impact the brain should be advanced in future neurodevelopmental studies.

It is important to highlight the heterogeneous nature of ELAs. It comprises many exposures that are often interlinked with each other and share commonalties^3,66^. As such, the ultimate effect of ELAs reflects an intricate interplay between the exposure and one´s characteristics, such as genetic make-up and personality factors, and its socioenvironmental embedding, e.g. social support^67^. So while we aimed to stratify the results on amygdala connectivity as much as possible, we must acknowledge that—by following stringent guideline criteria for inclusion^34,35,68^—the number of available experiments was insufficient for further identification of potential moderators, such as sex, other subtypes including psychopathology, timing of ELAs, prenatal ELAs or any specification of direction within the sensitivity analyses. Concerning prenatal ELAs, while their exclusion yielded no significant result in the pooled analysis, further direction-, task-, and sample-specific sub-analyses do reveal the same convergence clusters in the ACC and the hippocampus (Table 1), supporting the robustness of the presented results. We also acknowledge that the effects—including the potential teratogenic effects^69^—of prenatal substance exposure, such as marijuana or cocaine, on the developing brain may differ from those of other ELAs, like maltreatment or poverty. However, as both types of ELAs are linked to changes in similar brain areas^4,70,71^, as well as to altered amygdala connectivity^72^, this seems to suggests some level of equifinality. Future studies should further look into this. Of note, a potential limitation might be that no correction for multiple testing across all sensitivity analyses was performed, as it was considered as too conservative for this purpose.

Furthermore, due to the heterogeneity and interrelatedness of the different types of ELAs included in this meta-analysis, our findings may rather point to the amygdala as being a nosologically unspecific network hub targeted by many kinds of adversities with effects being present independent of specific samples. Thus, its affected connections to the hippocampus and ACC reflect alterations suggestive of transdiagnostic phenotypes that may imply a latent vulnerability signature, which unfolds during system-challenging stressful situations^2^. This understanding is well in line with therapeutic studies that show neurotrophic changes in the amygdala following electroconvulsive therapy^73,74^ across (patient) samples or other methodological considerations^74^. In light of this, we speculate that these recalibrations of amygdala connectivity as reported here might represent shared mechanisms of ELAs, that may be considered as a transdiagnostic risk correlate.

Given the recent discussion on different risk pathways dependent on ELA assessment^55,75^, the number of studies permitted a separate analysis on subjective and retrospectively assessed ELAs. However, the specification of neural embedding of ELAs that were either objectively or prospectively assessed was not possible and should therefore be focused in future meta-analyses. As the convergent effects reported in our work were mainly driven by findings from healthy participants and are thus not confounded by psychopathology, they should be assumed to reflect latent vulnerability signatures as similar alterations have been reported in clinical populations^76^. However, we cannot rule out that these altered neural phenotypes in relation to ELAs might be unrelated to psychological functioning altogether nor that they might reflect compensatory mechanisms supporting adaptive functioning later in life. After all, such recalibrated responses may be either adaptive or maladaptive based on environmental conditions^6^.

Previous studies indicate that amygdala-prefrontal connectivity develops with age, with the occurrence of a valence shift in task-related amygdala-prefrontal connectivity around the age of 10 years^77^. We therefore ensured that our result of decreased amygdala-ACC coupling still holds when participants under 10 years of age are excluded (see Table S5)—even though it must be noted that the literature is not consistent in reporting such age-related alterations of functional connectivity^78–80^. To further investigate the normative developmental pattern of this circuit, as well as to clearly delineate whether the neural recalibration associated with ELAs—mostly seen in children and adolescents—presents a delay of maturation or an acceleration, longitudinal studies are warranted^5^.

Taken together, our current meta-analysis provides robust evidence for decreased amygdala-ACC and altered amygdala-hippocampus connectivity in relation to ELAs. These results are in line with previous research (for a recent review, see Ref.^3^) and fits well within the theoretical framework of latent vulnerability^2^. This inherent neural plasticity to environmental exposures also holds a promise, as it might potentially enable normative recalibration and thereby promote resilience^67,81–83^. While initial evidence does exist in relation to the reversibility of the structural and functional alterations associated with ELAs^4,67,84^, future (longitudinal) studies should examine this neural malleability in light of potential therapeutic interventions.

## Methods and materials

This meta-analysis was preregistered with PROSPERO (CRD42018107773) and integrates all neuroimaging studies on the relation between ELAs and task-specific and resting-state brain connectivity using the amygdala as seed region. The study was conducted according to the PRISMA guidelines and current consensus guidelines for CMBAs^34–36^.

All screening, evaluation and data extraction procedures were performed by two independent authors (EK, NH) to reduce the chances of selection bias, and disagreements were resolved by consensus. The review protocol and data can be accessed upon request.

### Search strategy and study selection

Relevant articles published until October 2020 were identified through a comprehensive literature search using five databases (EMBASE, MEDLINE, PsychINFO/PsychARTICLES, Scopus and Web of Science). Search strategies were composed of the search terms ‘neuroimaging’ or ‘MRI’ AND ‘preterm birth’, ‘prenatal exposure’ or ‘adverse childhood experience’ with associated synonyms, using the keywords appropriate to each individual database (for full search terms, see Supplementary Table 1). In line with the definition of ELAs as deviations from the expected environment that require adaptation^85^ and that brain development does not start at birth but at conception^86^, we conceptualized ELA in this manuscript as developmental risk factors acting early in life and therefore included both pre- and postnatal exposures and specified the analyses in further steps (see below). Additional articles were identified by reference tracking of all included studies and consultation of relevant review articles^17,19,71,87,88^.

**Figure 2:**
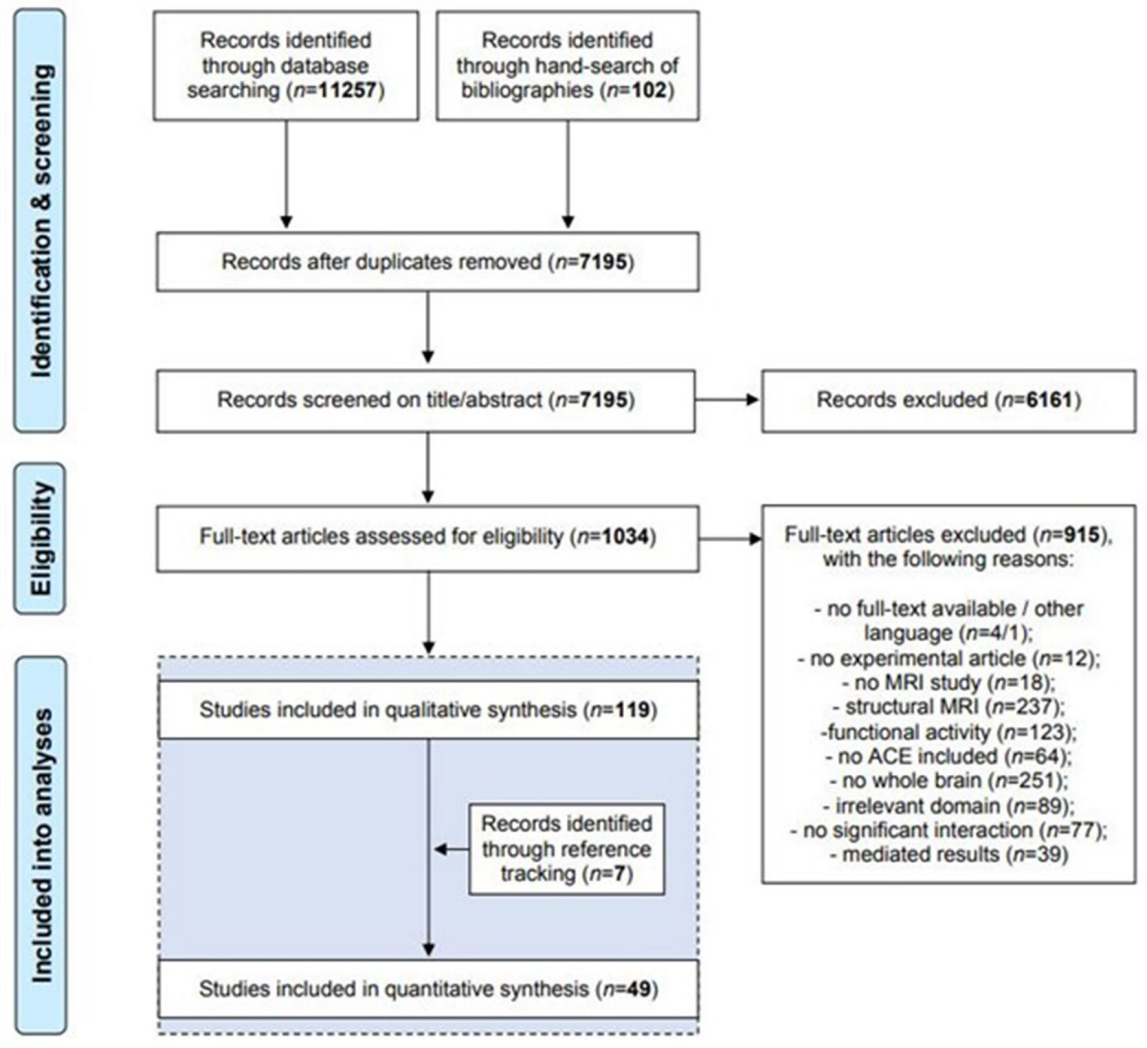
This meta-analysis was conducted according to current consensus guidelines for CBMAs and the PRISMA guidelines. Five databases were systematically searched from 2001 (which was chosen as the earliest date for studies with sufficient quality) to 22 October 2020, retrieving 49 eligible studies.

Studies were selected if (1) peer-reviewed, original articles were published in English language; (2) human brain connectivity was measured using the amygdala as seed-region; (3) prenatal exposures and/or postnatal experiences were assessed; and (4) whole-brain results with stereotactic coordinates were reported or if not, provided by the authors. As such, from the 7195 unique publications that were initially identified, 119 publications were included in the qualitative synthesis (Fig. 2; a detailed overview of the included and excluded studies is in Table 2 and Supplementary Table 2, respectively).

**Table 2:** Characteristics of all included studies.

Article	Sample				ELA				fMRI				
First author, year	N	Mean age (years)	Sex (%m)	Disorder	Time	Subtype (assessment)	Type	Assessment type	Paradigm	Contrast	Space	Hemi-sphere	# of foci
Barch, 2016	105	9.9	49	Healthy	Postnatal	Poverty (IN-ratio)	Continuous	Objective/prospective	Resting-state		Talairach	Both	7
Birn, 2014	27	26.6	100.0	17 PTSD	Postnatal	Maltreatment (CTQ)	Continuous	Subjective (self)/retrospective	Resting-state		Talairach	Both	6
Buchweitz, 2019	40	11.5	60.0	Healthy	Postnatal	Interpersonal violence (JVQ-R2)	Continuous	Subjective (self)/retrospective (last year)	Social cognition	Mental state > sex	MNI	Both	1
Cancel, 2017	46	32.6	67.4	21 SCZ	Postnatal	Maltreatment (CTQ)	Continuous	Subjective (self)/retrospective	Emotion processing	Negative vs positive	MNI	ns**	4
Cisler, 2017	56	14.9	0.0	10 PTSD	Postnatal	Assault/maltreatment (NSA/CTQ)	Continuous	Subjective (self)/retrospective	Resting-state		MNI	Left	1
Colich, 2017	98	11.4	43.1	Healthy	Postnatal	Traumatic stress (TESI-C)	Continuous	Objective/retrospective	Emotion processing	Labeling vs matching	MNI	Both	1
Dark, 2020	233	19.6	56.7	Healthy	Postnatal	Violence exposure (HPVE measure)	Continuous	Subjective (self)/prospective	Resting-state		Talairach	Left	1
Dean, 2014	15	38.4	53.3	15 addiction	Postnatal	Maltreatment (CTQ)	Continuous	Subjective (self)/retrospective	Resting-state		MNI	Both	12
Dégeilh, 2020	28	10.6	39.3	Healthy	Postnatal	SES (income-to-needs ratio)	Continuous	Objective/prospective	Resting-state		MNI	Both	3
Duque-Alarcon, 2019	33	32.2	0.0	18 BPD	Postnatal	Maltreatment (CTQ)	Continuous	Subjective (self)/retrospective	Resting-state		MNI	Right	1
Fan, 2014	18	27.8	100.0	Healthy	Postnatal	Maltreatment (CTQ)	Continuous	Subjective (self)/retrospective	Resting-state		MNI	Right	24
Fan, 2015	32	28.2	100.0	Healthy	Postnatal	Maltreatment (CTQ)	Continuous	Subjective (self)/retrospective	Stress processing	Stress > control	MNI	Both	11
Fonzo, 2013	33	39.3	0.0	33 PTSD	Postnatal	Maltreatment (CTQ)	Continuous	Subjective (self)/retrospective	Emotion processing	Negative > shape	Talairach	Left	3
Fortenbaugh, 2017	66	32.3	93.9	35 PTSD	Postnatal	Trauma (TLEQ)	Continuous	Subjective (self)/retrospective	Continuous performance		MNI	Both	2
Gard, 2020	77	20.0	100.0	Healthy	Postnatal	Neighborhood disadvantage (US Census/questionnaire)	Continuous	Objective/prospective	Emotion processing	Neutral vs baseline	MNI	Both	2
Gee, 2013	89	11.6	51.7	Healthy	Postnatal	Institutionalization	Categorical	Objective/retrospective	Emotion processing	Emotion vs shape	Talairach	Right	1
Grewen, 2015	63	213.62*	46.0	Healthy	Prenatal	Drug exposure (TLFB/urine screen)	Categorical	Objective/prospective	Resting-state		MNI	Left	1
Hanford, 2019	47	13.9	55.3	Healthy	Postnatal	Stressful life events (SLES questionnaire)	Continuous	Subjective (self)/retrospective (last year)	Emotion processing	Emotion vs shape	MNI	Both	3
Hanson, 2019	87	15.2	56.5	Healthy	Postnatal	SES (household family income)	Continuous	Objective/prospective	Resting-state		MNI	Both	3
Herringa, 2013	64	18.8	53.1	Healthy	Postnatal	Maltreatment (CTQ)	Continuous	Subjective (self)/retrospective	Resting-state		Talairach	Both	4
Herringa, 2016	132	18.6	47.7	38 IND, 12 EXD	Postnatal	Family adversity	Continuous	Subjective (caregiver)/prospective	Emotion processing	Positive vs neutral	MNI	Right	1
Holz, 2015	153	25.0	43.1	Healthy	Postnatal	Maltreatment (CTQ)	Continuous	Subjective (self)/retrospective	Emotion processing	Negative > neutral	MNI	Left	45
Javanbakht, 2015	52	23.7	53.0	Healthy	Postnatal	SES (income-to-needs ratio)	Continuous	Objective/retrospective	Emotion processing	Negative > positive	MNI	Left	1
Jedd, 2015	71	30.1	47.9	Healthy	Postnatal	Maltreatment (records/MMCI)	Categorical	Objective/prospective	Emotion processing	Emotion > shape	MNI	Both	10
Kaiser, 2018	70	26.4	0.0	36 MDD, 15 CM	Postnatal	Threat-related early life stress (TAC interview)	Continuous	Subjective (self)/retrospective	Resting-state		MNI	Both	2
Keding, 2016	53	14.3	32.2	25 PTSD	Postnatal	Trauma (KSADS)	Continuous	Subjective (self and caregiver)/retrospective	Emotion processing	Positive > shape	MNI	Right	2
Kopala-Sibley, 2020	62	10.3	54.0	Healthy	Postnatal	Maternal hostility (TTB)	Continuous	Objective/prospective	Emotion processing	Negative vs neutral	MNI	Both	3
Krause-Utz, 2014	37	28.6	0.0	20 BPD	Postnatal	Maltreatment (CTQ/PSDS)	Categorical	Subjective (self)/retrospective	Resting-state		MNI	Right	3
Kraynak, 2019	303	40.3	40.3	Healthy	Postnatal	Physical abuse (CTQ)	Continuous	Subjective (self)/retrospective	Resting-state		MNI	Both	1
La Buissonniere-Ariza, 2019	84	14.0	14.0	Healthy	Postnatal	Harsh parenting (NLSCY/PACOTIS)	Categorical	Subjective (caregiver)/prospective	Fear processing	CS + > CS-	MNI	Left	4
Lee, 2015	31	16.1	100.0	Healthy	Postnatal	Verbal abuse (VAS)	Continuous	Subjective (self)/retrospective	Emotion processing	Negative > neutral	MNI	Right	2
Li, 2019	41	16.6	51.2	Healthy	Prenatal	Cocaine exposure (self-report/urine screen)	Categorical	Subjective and objective/prospective	Resting-state		MNI	Both	1
Maier, 2020	50	24.5	48.0	Healthy	Postnatal	Maltreatment (CTQ)	Continuous	Subjective (self)/retrospective	Emotion processing	Stress vs sport	MNI	Right	3
Pagliaccio, 2015	120	11.2	51.7	54 ANX, 40 MDD, 41 EXD	Postnatal	Stress/trauma events (PAPA/CAPA)	Continuous	Subjective (self and caregiver)/retrospective	Resting-state		MNI	Left	1
Park, 2018	79	6.1	49.4	Healthy	Postnatal	Stressful life events (LES-C)	Continuous	Subjective (caregiver)/retrospective (last year)	Resting-state		MNI	Both	1
Peverill, 2019	57	16.9	38.6	Healthy	Postnatal	Maltreatment (CECA/CTQ)	Continuous	Subjective (self)/retrospective	Emotion processing	Negative > neutral	MNI	Left	2
Posner, 2016	64	40.6*	42.2	Healthy	Prenatal	Maternal depression (CES-D/HRSD)	Categorical	Objective/prospective	Resting-state		MNI	Both	2
Quidé, 2020	109	39.8	43.1	40 SCZ, 35 BD	Postnatal	Maltreatment (CTQ)	Continuous	Subjective (self)/retrospective	Emotion processing	Emotion > shape	MNI	Right	2
Radhakrishnan, 2020	22	30.1*	36.4	Healthy	Prenatal	Opioid exposure (records/questionnaire)	Categorical	Objective and subjective/prospective	Resting-state		MNI	Left	16
Salzwedel, 2015	119	306.3*	50.7	Healthy	Prenatal	Drug exposure (TLFB/urine toxicology)	Categorical	Objective and subjective/prospective	Resting-state		MNI	Left	1
Scheinost, 2016	26	263.6*	53.8	Healthy	Prenatal	Maternal depression/anxiety	Categorical	Objective/retrospective	Resting-state		MNI	Left	1
Silvers, 2016	89	12.1	38.2	Healthy	Postnatal	Institutionalization	Categorical	Objective/retrospective	Fear processing	CS + > CS-	MNI	Both	4
Thomason, 2015	42	12.6	32.0	Healthy	Postnatal	Trauma (TACSC-C)	Categorical	Subjective (self and caregiver)/retrospective	Resting-state		MNI	Both	36
Turesky, 2019	32	77.8*	53.1	Healthy	Postnatal	Poverty (IN-ratio)	Categorical	Objective and subjective/prospective	Resting-state		MNI	Both	1
van der Werff, 2013	88	38.3	27.3	32 MDD, 16 ANX, 24 CM	Postnatal	Maltreatment (NEMESIS interview)	Categorical	Subjective (self)/retrospective	Resting-state		MNI	Right	12
van der Werff, 2013	33	40.2	47.7	3 MDD, 3 ANX, 5 CM	Postnatal	Maltreatment (NEMESIS interview)	Categorical	Subjective (self)/retrospective	Resting-state		MNI	Left	19
van Rooij, 2020	69	10.8	47.8	11 PTSD	Postnatal	Trauma (TESI)/violence exposure (VEX-R)	Continuous	Subjective (self)/retrospective	Emotional response inhibition	Negative nogo vs neutral Go	MNI	Left	1
Wolf, 2016	48	14.2	39.6	24 PTSD	Postnatal	Trauma (KSADS)	Categorical	Subjective (self and caregiver)/retrospective	Emotion processing	Negative > shape	MNI	Left	2
Zielinski, 2018	61	21.3	0.0	20 PTSD, 17 ANX, 12 MDD	Postnatal	Violence exposure (NSA/NWS)	Categorical	Subjective (self)/retrospective	Resting-state		Talairach	Left	1

*Age reported in days, **ns = not specified (also not upon contacting authors).

ANX, anxiety; BD, bipolar disorder; BPD, borderline personality disorder; CECA, childhood experience of care and abuse; CES-D, center for epidemiologic studies depression; CM, comorbid MDD and anxiety; CTQ, childhood trauma questionnaire; EXD, externalizing disorder; HPVE, healthy passages violence exposure; HRSD, Hamilton rating scale for depression; IND, internalizing disorder; JVQ-(R2), juvenile victimization questionnaire (2nd revision); KSADS, kiddie schedule for affective disorders and schizophrenia; LES-C, life enjoyment and satisfaction for children; MDD, major depressive disorder; MCMI, millon clinical multiaxial inventory; NEMESIS, Netherlands mental health survey and incidence study; NLSCY, national longitudinal survey of children and youth; NSA, negative symptom assessment; NWS, PACOTIS, parental hostile-reactive behavior scale; PAPA/CAPA, preschool age psychiatric assessment /child and adolescent psychiatric assessment; PSDS, Pittsburgh service delivery study; PTSD, post-traumatic stress disorder; SCZ, schizophrenia; SES, social-economic status; SLES, stressful life events screening; TAC, trauma awareness center; TACSC-C, trauma assessment center screen checklist for children; TESI-C, traumatic events screening inventory for children; TLEQ, traumatic life events questionnaire; TLFB, timeline followback; TTB, teaching tasks battery; VAS, verbal abuse scale; VEX-R, violence exposure scale—revised.

### Data extraction and quality assessment

The following variables were extracted: bibliographic information, sample characteristics (e.g. sample size, age and sex) and methodological specifics regarding ELA assessment and fMRI procedures (e.g. acquisition, paradigm and analyses) (see Table 2). Criteria for quality assessment were based on best-practice guidelines^21,22^. Studies passed the stringent quality assessment if (1) sample characteristics were properly described; (2) ELA subtype and assessment was reported; (3) details about fMRI paradigm (resting-state or task-based) and acquisition (scanner, settings and seed-region) were provided; and (4) details about fMRI processing (motion correction), analyses (whole-brain and where applicable, contrast(s) of interest) and results (coordinates of peak foci of activation in MNI or Talairach space) were provided. When details were not explicitly mentioned in the article itself, corresponding authors were contacted.

After quality assessment, 49 unique publications were included in this meta-analysis, which comprised 3162 participants (weighted mean = 19.93 years), with 6 studies (12%) reporting exposure to prenatal adversities and 43 studies (88%) reporting exposure to postnatal adversities (Fig. 2; a detailed overview of the included and excluded studies is in Table 2 and Supplementary Table 2, respectively). Participants’ age for prenatal studies (*n* = 335) ranged from 30.1 days to 16.6 years (weighted mean = 3.12 years), with 47% identified as male and 100% reported as healthy. Participants’ age for postnatal studies (*n* = 2946) ranged from 77.8 days to 40.6 years (weighted mean = 21.8 years), with 47% identified as male and 58% reported as healthy. Of these postnatal studies, 44% reported on functional connectivity of children (*n* = 1479, weighted mean = 11.9 years), and 56% of that of adults (*n* = 1683, weighted mean = 29.02 years). Twenty-six (53%) studies reported on a task-based paradigm, with 96% using emotion recognition and processing tasks.

### Analytical procedures

Coordinate-based ALE analyses^89^ were conducted using GingerALE 3.0.2 based on CBMA consensus guidelines^34–36^. Activation likelihood estimation is amongst the most common algorithm for CMBAs and determines the convergence of reported coordinates across different experiments. This analysis considers activation foci not as single point but rather as centers of spatial probability distributions. An activation likelihood map is created based on the voxel-by-voxel union of these distributions, tested for statistical significance against randomly generated sets of foci^36,89^. ALE is a reliable way of combining results from multiple studies^38^ and was used successfully in previous CMBAs on the neurological consequences of ELAs (e.g. Refs.^4,5^).

First, Talairach coordinates were converted to MNI coordinates using the Lancaster transform (icbm2tal) function in GingerALE. For each analysis, coordinates from separate contrasts were grouped into one experiment to limit within-group effects^39^. As the samples of several studies (partially) overlapped, coordinates were organized by subjects to limit within-subject effects as well^38^. Full-width at half maximum (FWHM) was subject-based^89^ and the modeled activation maps were computed using as more conservative mask size. Cluster-level interference thresholding was used based on uncorrected p-values (p < 0.001), with a cluster-level family wise error corrected threshold of p < 0.05 and 1000 permutations. Results were visualized using Mango 4.1.

A global ALE analysis was performed across all experiments (*n* = 49 studies) to assess the relation between ELAs and functional brain connectivity irrespective of direction, hemisphere or paradigm. Regarding the latter, the joint analysis across heterogeneous designs, i.e. task-dependent and task-independent, reflects alterations of network connectivity across several mental states that are internally and externally determined. In a further step, we therefore specified the analyses into task-based (*n* = 22 studies) and resting-state (*n* = 27 studies) to delineate whether reported changes reflect common disturbances in neural mechanisms or paradigm-specific effects^90^. Additional sub-analyses based on direction (decrease: *n* = 31 studies, increase: *n* = 34 studies), hemisphere (left amygdala seed: *n* = 37 studies, right amygdala seed: *n* = 33 studies), age (adults: *n* = 23 studies, children: *n* = 26 studies), and disease status (healthy: *n* = 31 studies) were performed to further specify the effect (see Supplementary Table 4 for included studies per analysis). Moreover, separate sub-analyses on social ELAs (*n* = 35 studies) only, such as maltreatment, trauma, violence exposure as well as negative parenting, and on all postnatal ELAs excluding prenatal adversities were carried out (for a complete overview, see Supplementary Tables 3 and 4). Also, given recent evidence of a stronger link of subjective when compared to objective reports^75^ and that prospectively and retrospectively assessed populations may follow different risk pathways^55^, such sub-analyses were added as well (subjective self-report: *n* = 29 studies and retrospective report: *n* = 34 studies).

Subsequently, the resulting ALE clusters were functionally decoded using all eligible BrainMap experiments (*n* = 19,044) on healthy subjects coded in terms of all behavioral domains (cognition, action, perception, emotion, and interoception) and paradigm classes to avoid preselection bias^91–94^. Of note, this database reflects activity and not connectivity, and emotion regulation is not included. For functional characterization, we considered forward inference using a binomial test (significant at p < 0.05) that determines in which domains and classes the probability of finding activation in the respective cluster [P (Activation|Task)] was significantly higher than the overall chance, i.e., across the entire database [P(Activation)].

### Supplementary Information

Supplementary Figure S1.

Supplementary Information 1.

Supplementary Information 2.

## Data Availability

The datasets used and analysed during the current study available from the corresponding author on reasonable request.
