# Supplementary figures and images for: A coordinate-based meta-analysis of human amygdala connectivity alterations related to early life adversities
Source: Sci Rep. 2023 Oct 2;13:16541. doi: 10.1038/s41598-023-43057-2 (PMC10545708; doi:10.1038/s41598-023-43057-2)

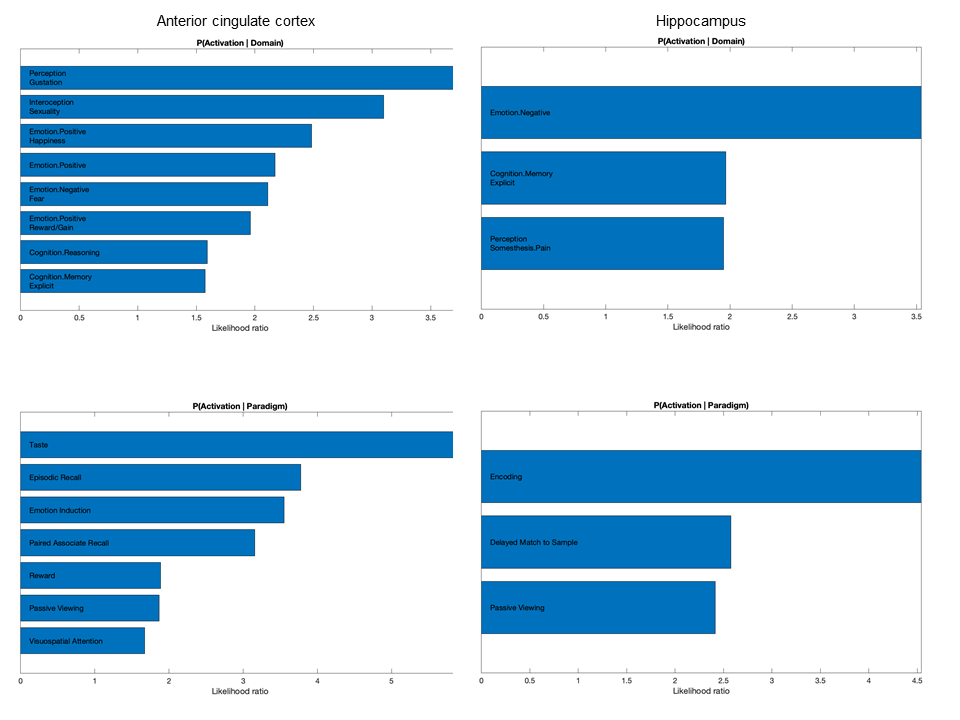

Supplement: Supplementary file 1 — Supplementary Figure S1. [file 41598_2023_43057_MOESM1_ESM.tif]
