# Supplementary material for: A coordinate-based meta-analysis of human amygdala connectivity alterations related to early life adversities
Source: Sci Rep. 2023 Oct 2;13:16541. doi: 10.1038/s41598-023-43057-2 (PMC10545708; doi:10.1038/s41598-023-43057-2)
Supplement: Supplementary file 2 — Supplementary Information 1. [file 41598_2023_43057_MOESM2_ESM.docx]

**A coordinate-based meta-analysis of human amygdala connectivity alterations related to early life adversities**

**Running Title: Early adversity and amygdala connectivity**

Kraaijenvanger, Eline J., MSc.^1^, Banaschewski, Tobias, Prof. Dr. Dr.^1^., Eickhoff, Simon B., Dr.^2,3^, Holz, Nathalie E., Dr.^1, 4, 5^

*^1^ Department of Child and Adolescent Psychiatry and Psychotherapy, Central Institute of Mental Health, Medical Faculty Mannheim/Heidelberg University, Mannheim, Germany*

*^2^ Institute of Systems Neuroscience, Medical Faculty, Heinrich Heine University Düsseldorf, Düsseldorf, Germany.*

*^3^ Institute of Neuroscience and Medicine, Brain & Behaviour (INM-7), Research Centre Jülich, Jülich, Germany.*

*^4^ Donders Institute, Radboud University, Nijmegen, the Netherlands*

*^5^ Radboud University Medical Centre, Nijmegen, the Netherlands*

Corresponding author at:

Department of Child and Adolescent Psychiatry and Psychotherapy, Central Institute of Mental Health, J5, 68159 Mannheim, Germany.

*Email address:* nathalie.holz@zi-mannheim.de (N.E. Holz)

**Supplemental material**

**Supplemental Results**

Functional characterization revealed that the ACC cluster of the overall analysis was significantly associated with several behavioral domains especially emotion (negative, fear, positive, happiness and reward) and higher cognitive functions (reasoning, memory) and with perception and interoception. In addition, it was associated with the paradigm classes taste, episodic recall, emotion induction, paired associate recall, reward, passive viewing and visuospatial attention. The hippocampus cluster was explicitly related to negative emotions and memory, as well as pain perception. Concerning paradigm classes, the hippocampal cluster was most activated during encoding, delayed match to sample and passive viewing (see Figure S1). Functional decoding of the clusters detected in the sub analyses yielded similar results.

**Supplemental Figure Legend**

**Figure S1.** Functional decoding for the ELA-related convergence cluster in the ACC (left) and the hippocampus (right). For each cluster, the figure shows the functional domain (upper part of each figure) and the paradigm (lower part).

**Supplementary Table 1. Search strategies**

| **Embase** | |
| --- | --- |
| *Search string* | ('child* maltreatment' OR 'child* abuse' OR 'child* neglect' OR 'childhood experience*' OR 'early life stress'/exp OR 'early life stress' OR 'early life adversit*' OR 'childhood trauma'/exp OR 'childhood trauma' OR 'social status'/exp OR 'prenatal exposure'/exp OR ‘prenatal stress’ OR 'prenatal drug exposure'/exp OR 'premature labor'/exp OR 'low birth weight'/exp) AND ('functional magnetic resonance imaging'/exp OR 'functional magnetic resonance imaging' OR 'functional imaging'/exp OR 'functional imaging' OR 'nuclear magnetic resonance imaging'/exp OR 'nuclear magnetic resonance imaging') |
| *Initial search (28-08-2018)* | 528 articles, with limitations ‘art/artip’ AND ‘human’ AND ‘1991-2018’ |
| *Novel search (10-03-2019) Novel search (22-10-2020)* | 494 articles, with limitations ‘art/artip’ AND ‘human’ AND ’2018-2020’ (*selected: sept.2018 – now)* 362 articles, with limitations ‘art/artip’ AND ‘human’ AND ‘2020-2020’ (*selected: march 2020 – now)* |
| **Pubmed** | |
| *Search string* | ("child maltreatment" OR "child mistreatment" OR "child abuse" OR "child neglect" OR "child abuse"[MeSH Terms] OR "adverse childhood experience*" OR "life change events"[MeSH Terms] OR "early life stress" OR "Adult Survivors of Child Adverse Events"[MeSH Terms] OR “premature birth*” OR “preterm birth*” OR “premature birth”[MeSH Terms] OR “infant, low birth weight”[MeSH Terms] OR “prenatal adversity” OR “prenatal exposure” OR “prenatal stress” OR “prenatal exposure delayed effects”[MeSH Terms] OR “maternal exposure”[MeSH Terms] OR “socioeconomic disadvantage*” OR “socioeconomic factors”[MeSH Terms] OR “poverty” OR “poverty”[MeSH Terms]) AND ("fmri" OR "magnetic resonance imaging" OR "functional mri*" OR “functional magnetic resonance imaging” OR "mri scan*" OR "magnetic resonance imaging"[MeSH Terms]) |
| *Initial search (28-08-2018)* | 2618 articles, with limitations ‘1991-now’ AND ‘humans’ |
| *Novel search (22-10-2020) Novel search (22-10-2020)* | 205 articles, with limitations ‘humans’ and ‘28-08-2018-now’ 81 articles, with limitations ‘humans’ and ’10-03-2020-now’ |
| **PsychINFO / PsychARTICLES** | |
| *Search string* | ((DE "Childhood Development" OR DE "Early Childhood Development" OR DE "Zone of Proximal Development") OR (DE "Child Neglect") OR (DE "Child Abuse") OR (DE "Early Experience" OR DE "Life Experiences") OR (DE "Life Changes") OR (DE "Premature Birth") OR (DE "Birth Weight") OR (DE "Prenatal Exposure") OR (DE "Poverty")) AND ((DE "Magnetic Resonance Imaging" OR DE "Functional Magnetic Resonance Imaging") OR (DE "Brain Size")) |
| *Initial search (28-08-2018)* | 1201 articles, with limitations ‘1991-now’ AND ‘human’ |
| *Novel search (10-03-2020) Novel search (22-10-2020)* | 140 articles, with limitations ’09-2018-now’ AND ‘human’ 68 articles, with limitations ’03-2020-now’ AND ‘human’ |
| **Scopus** | |
| *Search string* | TITLE-ABS-KEY ( "child* maltreatment" OR "child* neglect" OR "child* abuse" OR "adverse childhood experience*" OR "early life stress" OR "early life adversit*" OR "child* trauma" OR "prenatal exposure*" OR "prenatal stress" OR "preterm birth" OR "premature birth" OR "small gestational age" OR "low birth weight" OR "poverty" OR "social status" OR "socioeconomic status" ) AND TITLE-ABS-KEY ( "functional magnetic resonance imaging" OR "magnetic resonance imaging" OR "MRI" OR "fMRI" ) |
| *Initial search (28-08-2018)* | 2828 articles, with limitations ‘art/artip’ AND ‘human(s)’ |
| *Novel search (10-03-2020) Novel search (22-10-2020)* | 401 articles, with limitations ‘art/artip’ AND ‘human(s)’ AND ‘2018-2020’ *(selected: sept.2018 – now)* 178 articles, with limitations ‘ar/artip’ AND ‘human(s)’ AND ‘2020-2020’ (*selected: march 2020 – now)* |
| **Web of Science** | |
| *Search string* | TS=( "child* maltreatment" OR "child* neglect" OR "child* abuse" OR "adverse childhood experience*" OR "early life stress" OR "early life adversit*" OR "child* trauma" OR "prenatal exposure*" OR "prenatal stress" OR "preterm birth" OR "premature birth" OR "small gestational age" OR "low birth weight" OR "poverty" OR "social status" OR "socioeconomic status" ) AND TS=( "functional magnetic resonance imaging" OR "magnetic resonance imaging" OR "MRI" OR "fMRI" ) |
| *Initial search (28-08-2018)* | 1755 articles, with limitations ‘1991-2018’ AND ‘article’ |
| *Novel search (10-03-2020) Novel search (22-10-2020)* | 313 articles, with limitations ‘2018-2020’ AND ‘article’ (*selected: sept.2018 – now*) 155 articles, with limitations ‘2020-2020’ AND ‘article’ (*selected: march 2020 – now)* |

**Supplementary Table 2. Excluded full text studies.**

| \| **Name** \| **Title** \| **Year** \| **Reason of exclusion** \| \| --- \| --- \| --- \| --- \| \| Aanes \| Reduced hippocampal subfield volumes and memory function in school-aged children born preterm with very low birthweight (VLBW). \| 2019 \| structural MRI \| \| Aas \| Childhood trauma is associated with increased brain responses to emotionally negative as compared with positive faces in patients with psychotic disorders \| 2017 \| functional activity \| \| Aas \| BDNF val66met modulates the association between childhood trauma, cognitive and brain abnormalities in psychoses \| 2013 \| no whole brain \| \| Aas \| Interplay between childhood trauma and BDNF val66met variants on blood BDNF mRNA levels and on hippocampus subfields volumes in schizophrenia spectrum and bipolar disorders \| 2014 \| no whole brain \| \| Aas \| Is there a link between childhood trauma, cognition, and amygdala and hippocampus volume in first-episode psychosis? \| 2012 \| no whole brain \| \| Abercrombie \| Neural signaling of cortisol, childhood emotional abuse, and depression-related memory bias. \| 2018 \| irrelevant domain \| \| Achterberg \| Longitudinal changes in DLPFC activation during childhood are related to decreased aggression following social rejection \| 2020 \| functional activity \| \| Acosta \| Prenatal maternal depressive symptoms are associated with smaller amygdalar volumes of four-year-old children. \| 2020 \| structural MRI \| \| Acosta \| Sex-specific association between infant caudate volumes and a polygenic risk score for major depressive disorder. \| 2020 \| structural MRI \| \| Adise \| Is brain response to food rewards related to overeating? A test of the reward surfeit model of overeating in children. \| 2018 \| no experimental article \| \| Aghajani \| Abnormal functional architecture of amygdala-centered networks in adolescent posttraumatic stress disorder \| 2016 \| no whole brain \| \| Ahn \| What is the impact of child abuse on gray matter abnormalities in individuals with major depressive disorder: a case control study. \| 2016 \| irrelevant domain \| \| Akyuz \| Structural brain imaging in children and adolescents following prenatal cocaine exposure: preliminary longitudinal findings. \| 2014 \| no significant interaction \| \| Alexander \| Interaction of the serotonin transporter-linked polymorphic region and environmental adversity: increased amygdala-hypothalamus connectivity as a potential mechanism linking neural and endocrine hyperreactivity. \| 2012 \| no ACE \| \| Alexander \| Changes in neonatal regional brain volume associated with preterm birth and perinatal factors. \| 2019 \| structural MRI \| \| Alnæs \| Patterns of sociocognitive stratification and perinatal risk in the child brain. \| 2020 \| structural MRI \| \| Alves \| Sex differences in the association between prenatal exposure to maternal obesity and hippocampal volume in children. \| 2020 \| structural MRI \| \| Anblagan \| Maternal smoking during pregnancy and fetal organ growth: a magnetic resonance imaging study. \| 2013 \| structural MRI \| \| Andero \| Amygdala-dependent fear is regulated by Oprl1 in mice and humans with PTSD. \| 2013 \| no whole brain \| \| Andersen \| Preliminary evidence for sensitive periods in the effect of childhood sexual abuse on regional brain development. \| 2008 \| no whole brain \| \| Anderson \| Abnormal T2 relaxation time in the cerebellar vermis of adults sexually abused in childhood: potential role of the vermis in stress-enhanced risk for drug abuse. \| 2002 \| no whole brain \| \| Archibald \| Brain dysmorphology in individuals with severe prenatal alcohol exposure \| 2001 \| no whole brain \| \| Armio \| Amygdala subnucleus volumes in psychosis high-risk state and first-episode psychosis: Amygdala subnuclei and psychosis. \| 2020 \| structural MRI \| \| Assari \| Family income mediates the effect of parental education on adolescents’ hippocampus activation during an n-back memory task. \| 2020 \| functional activity \| \| Astley \| Functional magnetic resonance imaging outcomes from a comprehensive magnetic resonance study of children with fetal alcohol spectrum disorders \| 2009 \| no whole brain \| \| Astley \| Magnetic resonance imaging outcomes from a comprehensive magnetic resonance study of children with fetal alcohol spectrum disorders. \| 2009 \| structural MRI \| \| Aust \| How emotional abilities modulate the influence of early life stress on hippocampal functioning. \| 2014 \| no main effect \| \| Aust \| Differential effects of early life stress on hippocampus and amygdala volume as a function of emotional abilities. \| 2014 \| no whole brain \| \| Autti-Rämö \| MRI findings in children with school problems who had been exposed prenatally to alcohol. \| 2002 \| no whole brain \| \| Avants \| Effects of heavy in utero cocaine exposure on adolescent caudate morphology \| 2007 \| no whole brain \| \| Avants \| Relation of childhood home environment to cortical thickness in late adolescence: specificity of experience and timing. \| 2015 \| no whole brain \| \| Bach \| Effects of social exclusion and physical pain in chronic opioid maintenance treatment: fMRI correlates. \| 2019 \| functional activity \| \| Bachi \| Reduced orbitofrontal gray matter concentration as a marker of premorbid childhood trauma in cocaine use disorder. \| 2018 \| structural MRI \| \| Badura-Brack \| Hippocampal and parahippocampal volumes vary by sex and traumatic life events in children. \| 2020 \| structural MRI \| \| Baker \| Impact of early vs. late childhood early life stress on brain morphometrics. \| 2013 \| no whole brain \| \| Baldaçera \| Reduction of anterior cingulate in adults with urban violence-related PTSD. \| 2014 \| no whole brain \| \| Baldwin \| Neural correlates of healing prayers, depression and traumatic memories: A preliminary study. \| 2016 \| no ACE \| \| Banihashemi \| Childhood physical abuse predicts stressor-evoked activity within central visceral control regions. \| 2013 \| functional activity \| \| Banihashemi \| Interactions between childhood maltreatment and combat exposure trauma on stress-related activity within the cingulate cortex: a pilot study. \| 2020 \| structural MRI \| \| Baranger \| PER1 rs3027172 genotype interacts with early life stress to predict problematic alcohol use, but not reward-related ventral striatum activity. \| 2016 \| no whole brain \| \| Barch \| Early childhood adverse experiences, inferior frontal gyrus connectivity, and the trajectory of externalizing psychopathology. \| 2018 \| no whole brain \| \| Barker \| Childhood adversity and hippocampal and amygdala volumes in a population at familial high risk of schizophrenia. \| 2016 \| no whole brain \| \| Barker \| Childhood adversity and cortical thickness and surface area in a population at familial high risk of schizophrenia. \| 2016 \| structural MRI \| \| Barnes-Davis \| Extremely preterm children exhibit increased interhemispheric connectivity for language: findings from fMRI-constrained MEG analysis. \| 2018 \| no MRI \| \| Barnes-Davis \| Rewiring the extremely preterm brain: Altered structural connectivity relates to language function. \| 2020 \| no MRI \| \| Bartlett \| Depression severity over 27 months in adolescent girls Is predicted by stress-linked cortical morphology. \| 2019 \| structural MRI \| \| Bauer \| Cerebellar volume and cognitive functioning in children who experienced early deprivation. \| 2009 \| no whole brain \| \| Beblo \| Functional MRI correlates of the recall of unresolved life events in borderline personality disorder. \| 2006 \| no ACE \| \| Benedetti \| The serotonin transporter genotype modulates the relationship between early stress and adult suicidality in bipolar disorder. \| 2014 \| no main effect \| \| Benedetti \| Emotional reactivity in chronic schizophrenia: structural and functional brain correlates and the influence of adverse childhood experiences. \| 2011 \| no whole brain \| \| Benedetti \| Caudate gray matter volume in obsessive-compulsive disorder is influenced by adverse childhood experiences and ongoing drug treatment. \| 2012 \| structural MRI \| \| Benetti \| Attachment style, affective loss and gray matter volume: A voxel-based morphometry study. \| 2010 \| no ACE \| \| Bennett \| Prenatal tobacco exposure predicts differential brain function during working memory in early adolescence: a preliminary investigation. \| 2013 \| functional activity \| \| Bennett \| Response inhibition among early adolescents prenatally exposed to tobacco: an fMRI study. \| 2009 \| functional activity \| \| Bermingham \| Effect of genetic variant in BICC1 on functional and structural brain changes in depression. \| 2012 \| no main effect \| \| Betancourt \| Effect of socioeconomic status (SES) disparity on neural development in female African-American infants at age 1month. \| 2016 \| no whole brain \| \| Biazoli \| Socioeconomic status in children is associated with spontaneous activity in right superior temporal gyrus. \| 2019 \| irrelevant measure \| \| Biffen \| Reductions in corpus callosum volume partially mediate effects of prenatal alcohol exposure on IQ \| 2018 \| structural MRI \| \| Biffen \| Validity of automated FreeSurfer segmentation compared to manual tracing in detecting prenatal alcohol exposure-related subcortical and corpus callosal alterations in 9- to 11-year-old children \| 2020 \| structural MRI \| \| Bilek \| Deficient amygdala habituation to threatening stimuli in borderline personality disorder relates to adverse childhood experiences \| 2019 \| functional activity \| \| Binter \| Exposure of pregnant women to organophosphate insecticides and child motor inhibition at the age of 10-12 years evaluated by fMRI. \| 2020 \| functional activity \| \| Binter \| Prenatal exposure to glycol ethers and motor inhibition function evaluated by functional MRI at the age of 10 to 12 years in the PELAGIE mother-child cohort. \| 2019 \| functional activity \| \| Birn \| Early childhood stress exposure, reward pathways, and adult decision making. \| 2016 \| functional activity \| \| Bjorkquist \| Cingulate gyrus morphology in children and adolescents with fetal alcohol spectrum disorders. \| 2010 \| structural MRI \| \| Bjørnebekk \| Development of children born to mothers with mental health problems: Subcortical volumes and cognitive performance at 4½ years. \| 2015 \| no whole brain \| \| Blair \| Association of different types of childhood maltreatment with emotional responding and response control among youths. \| 2019 \| functional activity \| \| Blair \| Sexual abuse in adolescents is associated with atypically increased responsiveness within regions implicated in self-referential and emotional processing to approaching animate threats \| 2020 \| functional activity \| \| Blasi \| Early life adversities and borderline intellectual functioning negatively impact limbic system connectivity in childhood: A connectomics-based study \| 2020 \| structural MRI \| \| Bleker \| Brain magnetic resonance imaging findings in children after antenatal maternal depression treatment, a longitudinal study built on a pilot randomized controlled trial. \| 2019 \| structural MRI \| \| Blesa \| Early breast milk exposure modifies brain connectivity in preterm infants. \| 2019 \| no main effect \| \| Boecker \| Impact of early life adversity on reward processing in young adults: EEG-fMRI results from a prospective study over 25 years. \| 2014 \| no whole brain \| \| Boecker-Schlier \| Interaction between COMT Val(158)Met polymorphism and childhood adversity affects reward processing in adulthood. \| 2016 \| no main effect \| \| Bogdan \| Mineralocorticoid receptor Iso/Val (rs5522) genotype moderates the association between previous childhood emotional neglect and amygdala reactivity. \| 2012 \| no whole brain \| \| Bomyea \| Neurocognitive markers of childhood abuse in individuals with PTSD: Findings from the INTRuST Clinical Consortium \| 2020 \| structural MRI \| \| Booij \| DNA methylation of the serotonin transporter gene in peripheral cells and stress-related changes in hippocampal volume: a study in depressed patients and healthy controls. \| 2015 \| no whole brain \| \| Bookstein \| Damage to the human cerebellum from prenatal alcohol exposure: The anatomy of a simple biometrical explanation. \| 2006 \| no whole brain \| \| Bookstein \| Geometric morphometrics of corpus callosum and subcortical structures in the fetal-alcohol-affected brain. \| 2001 \| no whole brain \| \| Bookstein \| Midline corpus callosum is a neuroanatomical focus of fetal alcohol damage \| 2002 \| no whole brain \| \| Boronat \| Correlation between morphological MRI findings and specific diagnostic categories in fetal alcohol spectrum disorders. \| 2017 \| irrelevant domain \| \| Bos \| Cortisol administration increases hippocampal activation to infant crying in males depending on childhood neglect. \| 2014 \| irrelevant domain \| \| Brambilla \| Anatomical MRI study of borderline personality disorder patients. \| 2004 \| structural MRI \| \| Bremner \| The relationship between cognitive and brain changes in posttraumatic stress disorder. \| 2006 \| no experimental article \| \| Bremner \| MRI and PET study of deficits in hippocampal structure and function in women with childhood sexual abuse and posttraumatic stress disorder. \| 2003 \| no whole brain \| \| Brito \| Associations between cortical thickness and neurocognitive skills during childhood vary by family socioeconomic factors. \| 2017 \| no whole brain \| \| Brody \| Protective prevention effects on the association of poverty with brain development. \| 2017 \| no whole brain \| \| Brooks \| Childhood adversity is linked to differential brain volumes in adolescents with alcohol use disorder: a voxel-based morphometry study. \| 2014 \| structural MRI \| \| Brooks \| Early-life adversity and orbitofrontal and cerebellar volumes in adults with obsessive-compulsive disorder: voxel-based morphometry study. \| 2016 \| structural MRI \| \| Brossard-Racine \| Early extra-uterine exposure alters regional cerebellar growth in infants born preterm. \| 2019 \| structural MRI \| \| Brown \| Adolescence and the trajectory of alcohol use: Basic to clinical studies. \| 2004 \| no experimental article \| \| Bruce \| Patterns of brain activation in foster children and nonmaltreated children during an inhibitory control task. \| 2013 \| functional activity \| \| Brunetti \| Response inhibition failure to visual stimuli paired with a “single-type” stressor in PTSD patients: an fMRI pilot study. \| 2015 \| no ACE \| \| Buchheim \| Neural correlates of attachment trauma in borderline personality disorder: a functional magnetic resonance imaging study. \| 2008 \| no ACE \| \| Buchheim \| Neural response during the activation of the attachment system in patients with borderline personality disorder: An fMRI study. \| 2016 \| no ACE \| \| Bücker \| Childhood maltreatment and corpus callosum volume in recently diagnosed patients with bipolar I disorder: Data from the Systematic Treatment Optimization Program for Early Mania (STOP-EM). \| 2014 \| no whole brain \| \| Buss \| Maternal cortisol over the course of pregnancy and subsequent child amygdala and hippocampus volumes and affective problems. \| 2012 \| no whole brain \| \| Buss \| High pregnancy anxiety during mid-gestation is associated with decreased gray matter density in 6-9-year-old children. \| 2010 \| structural MRI \| \| Busso \| Child abuse, neural structure, and adolescent psychopathology: a longitudinal study. \| 2017 \| no whole brain \| \| Butler \| Community violence exposure correlates with smaller gray matter volume and lower IQ in urban adolescents. \| 2018 \| structural MRI \| \| Butterworth \| The association between financial hardship and amygdala and hippocampal volumes: results from the PATH through life project. \| 2012 \| no whole brain \| \| Calderón-Garcidueñas \| Exposure to severe urban air pollution influences cognitive outcomes, brain volume and systemic inflammation in clinically healthy children. \| 2011 \| no whole brain \| \| Calvo \| Reduced hippocampal volume in adolescents with psychotic experiences: A longitudinal population-based study \| 2020 \| structural MRI \| \| Cancel \| Childhood neglect predicts disorganization in schizophrenia through grey matter decrease in dorsolateral prefrontal cortex. \| 2015 \| structural MRI \| \| Cannon \| Fetal hypoxia and structural brain abnormalities in schizophrenic patients, their siblings, and controls. \| 2002 \| structural MRI \| \| Cara \| An fMRI study of inhibitory control and the effects of exposure to violence in Latin-American early adolescents: alterations in frontoparietal activation and performance \| 2019 \| functional activity \| \| Carballedo \| Brain-derived neurotrophic factor Val66Met polymorphism and early life adversity affect hippocampal volume. \| 2013 \| no whole brain \| \| Carballedo \| Early life adversity is associated with brain changes in subjects at family risk for depression. \| 2012 \| no whole brain \| \| Cardenas \| Automated cerebellar segmentation: Validation and application to detect smaller volumes in children prenatally exposed to alcohol. \| 2014 \| no whole brain \| \| Carrión \| Reduced hippocampal activity in youth with posttraumatic stress symptoms: An fMRI study. \| 2010 \| no whole brain \| \| Carrión \| Stress predicts brain changes in children: A pilot longitudinal study on youth stress, posttraumatic stress disorder, and the hippocampus. \| 2007 \| no whole brain \| \| Carrión \| Attenuation of frontal asymmetry in pediatric posttraumatic stress disorder. \| 2001 \| structural MRI \| \| Carrión \| Converging evidence for abnormalities of the prefrontal cortex and evaluation of midsagittal structures in pediatric posttraumatic stress disorder: An MRI study. \| 2009 \| structural MRI \| \| Carrión \| Decreased prefrontal cortical volume associated with increased bedtime cortisol in traumatized youth. \| 2010 \| structural MRI \| \| Catena \| On the relationship between head circumference, brain size, prenatal long-chain PUFA/5-methyltetrahydrofolate supplementation and cognitive abilities during childhood. \| 2019 \| structural MRI \| \| Cavanagh \| Socioeconomic status and the cerebellar grey matter volume. Data from a well-characterised population sample. \| 2013 \| no whole brain \| \| Chalavi \| Similar cortical but not subcortical gray matter abnormalities in women with posttraumatic stress disorder with versus without dissociative identity disorder. \| 2015 \| no ACE \| \| Chalavi \| Abnormal hippocampal morphology in dissociative identity disorder and post-traumatic stress disorder correlates with childhood trauma and dissociative symptoms. \| 2015 \| no whole brain \| \| Chaney \| Effect of childhood maltreatment on brain structure in adult patients with major depressive disorder and healthy participants. \| 2014 \| no significant interaction \| \| Chang \| Smaller subcortical volumes and cognitive deficits in children with prenatal methamphetamine exposure. \| 2004 \| no significant interaction \| \| Chau \| Hippocampus, amygdala, and thalamus volumes in very preterm children at 8 years: Neonatal pain and genetic variation. \| 2019 \| structural MRI \| \| Chen \| Increased inhibition of the amygdala by the mPFC may reflect a resilience factor in post-traumatic stress disorder: A resting-state fMRI granger causality analysis. \| 2018 \| no ACE \| \| Chen \| Understanding specific effects of prenatal alcohol exposure on brain structure in young adults. \| 2012 \| structural MRI \| \| Cheng \| Functional MRI of human eyeblink classical conditioning in children with fetal alcohol spectrum disorders. \| 2017 \| irrelevant domain \| \| Chura \| Organizational effects of fetal testosterone on human corpus callosum size and asymmetry. \| 2010 \| no whole brain \| \| Cisler \| Differential roles of the salience network during prediction error encoding and facial emotion processing among female adolescent assault victims. \| 2019 \| functional activity \| \| Clark \| High early life stress and aberrant amygdala activity: risk factors for elevated neuropsychiatric symptoms in HIV plus adults. \| 2017 \| no significant interaction \| \| Clark \| Effects of HIV and early life stress on amygdala morphometry and neurocognitive function. \| 2012 \| no whole brain \| \| Clark \| Early life stress-related elevations in reaction time variability are associated with brain volume reductions in HIV plus adults. \| 2018 \| structural MRI \| \| Cohen \| Early life stress and morphometry of the adult anterior cingulate cortex and caudate nuclei. \| 2006 \| no whole brain \| \| Coles \| Memory and brain volume in adults prenatally exposed to alcohol \| 2011 \| no whole brain \| \| Colich \| The association between early life stress and prefrontal cortex activation during implicit emotion regulation is moderated by sex in early adolescence. \| 2017 \| functional activity \| \| Colle \| Early life adversity is associated with a smaller hippocampus in male but not female depressed in-patients: a case-control study. \| 2017 \| no whole brain \| \| Conant \| The relationship between maternal education and the neural substrates of phoneme perception in children: Interactions between socioeconomic status and proficiency level. \| 2017 \| irrelevant domain \| \| Cope \| Developmental maturation of inhibitory control circuitry in a high-risk sample: A longitudinal fMRI study. \| 2020 \| functional activity \| \| Corbo \| Reduced cortical thickness in veterans exposed to early life trauma. \| 2014 \| no significant interaction \| \| Corcoles-Parada \| Born too early and too small: higher order cognitive function and brain at risk at ages 8-16 \| 2019 \| structural MRI \| \| Corral-Frías \| Stress-related anhedonia is associated with ventral striatum reactivity to reward and transdiagnostic psychiatric symptomatology. \| 2015 \| no whole brain \| \| Cortese \| Magnetic resonance and spectroscopic imaging in prenatal alcohol-exposed children: preliminary findings in the caudate nucleus. \| 2006 \| no significant interaction \| \| Crossley \| Structural brain abnormalities in schizophrenia in adverse environments: examining the effect of poverty and violence in six Latin American cities. \| 2020 \| structural MRI \| \| Croy \| Women with a history of childhood maltreatment exhibit more activation in association areas following non-traumatic olfactory stimuli: a fMRI study. \| 2010 \| irrelevant domain \| \| Crozier \| Neural correlates of cognitive and affective processing in maltreated youth with posttraumatic stress symptoms: does gender matter? \| 2014 \| functional activity \| \| Cservenka \| Emotional processing and brain activity in youth at high risk for alcoholism. \| 2014 \| functional activity \| \| Cservenka \| Atypical frontal lobe activity during verbal working memory in youth with a family history of alcoholism. \| 2012 \| irrelevant domain \| \| Cullen \| Pituitary gland volume and psychosocial stress among children at elevated risk for schizophrenia. \| 2015 \| no whole brain \| \| Cullen \| Polygenic risk for neuropsychiatric disease and vulnerability to abnormal deep grey matter development. \| 2019 \| structural MRI \| \| Dahmen \| Effects of early-life adversity on hippocampal structures and associated HPA axis functions. \| 2018 \| FT not found \| \| Dalvie \| The BDNF p.Val66Met polymorphism, childhood trauma, and brain volumes in adolescents with alcohol abuse. \| 2014 \| no main effect \| \| Daniels \| Neural correlates and predictive power of trait resilience in an acutely traumatized sample: a pilot investigation. \| 2012 \| no whole brain \| \| Dannlowski \| Childhood maltreatment is associated with an automatic negative emotion processing bias in the amygdala. \| 2013 \| functional activity \| \| Dannlowski \| Limbic scars: long-term consequences of childhood maltreatment revealed by functional and structural magnetic resonance imaging. \| 2012 \| functional activity \| \| Dannlowski \| Disadvantage of social sensitivity: interaction of oxytocin receptor genotype and child maltreatment on brain structure. \| 2016 \| structural MRI \| \| Davis \| Prenatal maternal cortisol concentrations predict neurodevelopment in middle childhood. \| 2017 \| no whole brain \| \| Davis \| Fetal glucocorticoid exposure is associated with preadolescent brain development. \| 2013 \| structural MRI \| \| Davis \| Prenatal maternal stress, child cortical thickness, and adolescent depressive symptoms. \| 2020 \| structural MRI \| \| de Araújo \| Tubulin Polymerization Promoting Protein (TPPP) gene methylation and corpus callosum measures in maltreated children \| 2020 \| structural MRI \| \| De Asis-Cruz \| Functional brain connectivity in ex utero premature infants compared to in utero fetuses. \| 2020 \| no ACE \| \| De Bellis \| Neural substrates for processing task-irrelevant emotional distracters in maltreated adolescents with depressive disorders: a pilot study. \| 2012 \| functional activity \| \| De Bellis \| Demographic, maltreatment, and neurobiological correlates of PTSD symptoms in children and adolescents. \| 2010 \| no whole brain \| \| De Bellis \| Superior temporal gyrus volumes in maltreated children and adolescents with PTSD. \| 2002 \| no whole brain \| \| De Bellis \| A pilot longitudinal study of hippocampal volumes in pediatric maltreatment-related posttraumatic stress disorder. \| 2001 \| structural MRI \| \| De Bellis \| Brain structures in pediatric maltreatment-related posttraumatic stress disorder: a sociodemographically matched study. \| 2002 \| structural MRI \| \| De Bellis \| Cerebellar volumes in pediatric maltreatment-related posttraumatic stress disorder. \| 2006 \| structural MRI \| \| De Bellis \| Posterior structural brain volumes differ in maltreated youth with and without chronic posttraumatic stress disorder. \| 2015 \| structural MRI \| \| De Bellis \| Sex differences in brain maturation in maltreatment-related pediatric posttraumatic stress disorder. \| 2003 \| structural MRI \| \| De Brito \| Reduced orbitofrontal and temporal grey matter in a community sample of maltreated children. \| 2013 \| structural MRI \| \| De Guio \| A study of cortical morphology in children with fetal alcohol spectrum disorders. \| 2014 \| structural MRI \| \| de Rooij \| Prenatal famine exposure has sex-specific effects on brain size \| 2016 \| structural MRI \| \| de Zeeuw \| Prenatal exposure to cigarette smoke or alcohol and cerebellum volume in attention-deficit/hyperactivity disorder and typical development. \| 2012 \| structural MRI \| \| Deater-Deckard \| Poverty and puberty: a neurocognitive study of inhibitory control in the transition to adolescence. \| 2019 \| functional activity \| \| DelDonno \| Influence of childhood adversity, approach motivation traits, and depression on individual differences in brain activation during reward anticipation. \| 2019 \| functional activity \| \| Demers \| Separable effects of childhood maltreatment and adult adaptive functioning on amygdala connectivity during emotion processing. \| 2018 \| no significant interaction \| \| Demers \| Dorsal anterior cingulate thickness is related to alexithymia in childhood trauma-related PTSD. \| 2015 \| no whole brain \| \| Demir \| Parental socioeconomic status and the neural basis of arithmetic: differential relations to verbal and visuo-spatial representations. \| 2015 \| irrelevant domain \| \| Demir-Lira \| Neural correlates of math gains vary depending on parental socioeconomic status (SES). \| 2016 \| irrelevant domain \| \| Dennison \| Neurobehavioral markers of resilience to depression amongst adolescents exposed to child abuse. \| 2016 \| no whole brain \| \| Derauf \| Subcortical and cortical structural central nervous system changes and attention processing deficits in preschool-aged children with prenatal methamphetamine and tobacco exposure. \| 2012 \| no significant interaction \| \| Di Iorio \| Hypothalamic-pituitary-adrenal axis genetic variation and early stress moderates amygdala function. \| 2017 \| no whole brain \| \| Díaz-Arteche \| Associations between early life stress and anterior pituitary gland volume development - A novel index of long-term hypothalamic-pituitary-adrenal axis functioning \| 2020 \| structural MRI \| \| Dillon \| Childhood adversity is associated with left basal ganglia dysfunction during reward anticipation in adulthood. \| 2009 \| no whole brain \| \| Disselhoff \| Inhibition is associated with whole-brain structural brain connectivity on network level in school-aged children born very preterm and at term. \| 2020 \| structural MRI \| \| Diwadkar \| Differences in cortico-striatal-cerebellar activation during working memory in syndromal and nonsyndromal children with prenatal alcohol exposure. \| 2013 \| no whole brain \| \| Dodge \| Prenatal alcohol exposure and interhemispheric transfer of tactile information: Detroit and Cape Town findings. \| 2009 \| no whole brain \| \| Dodge \| Reduced Hippocampal Volumes Partially Mediate Effects of Prenatal Alcohol Exposure on Spatial Navigation on a Virtual Water Maze Task in Children. \| 2020 \| structural MRI \| \| Donald \| Alcohol exposure in utero is associated with decreased gray matter volume in neonates. \| 2016 \| structural MRI \| \| Dotterer \| Connections that characterize callousness: Affective features of psychopathy are associated with personalized patterns of resting-state network connectivity. \| 2020 \| no ACE \| \| Dougherty \| Smoking mediates the relationship between SES and brain volume: The CARDIA study \| 2020 \| structural MRI \| \| Driessen \| One-year functional magnetic resonance imaging follow-up study of neural activation during the recall of unresolved negative life events in borderline personality disorder. \| 2009 \| no ACE \| \| Driessen \| Magnetic resonance imaging volumes of the hippocampus and the amygdala in women with borderline personality disorder and early traumatization. \| 2000 \| no whole brain \| \| du Plessis \| Childhood trauma and hippocampal subfield volumes in first-episode schizophrenia and healthy controls. \| 2020 \| structural MRI \| \| Duarte \| Gray matter brain volumes in childhood-maltreated patients with bipolar disorder type I: A voxel-based morphometric study. \| 2016 \| no significant interaction \| \| Duncan \| Negative childhood experiences alter a prefrontal-insular-motor cortical network in healthy adults: A preliminary multimodal rsfMRI-fMRI-MRS-dMRI study. \| 2015 \| irrelevant domain \| \| Duricic \| Social, clinical, and radiological characteristics of physical abuse of children under three years of age hospitalized in a tertiary health institution. \| 2017 \| no MRI \| \| Duval \| Childhood poverty is associated with altered hippocampal function and visuospatial memory in adulthood. \| 2017 \| no whole brain \| \| Dvir \| Psychiatric Symptomatology, Mood Regulation, and Resting State Functional Connectivity of the Amygdala: Preliminary Findings in Youth With Mood Disorders and Childhood Trauma \| 2020 \| no significant interaction \| \| Eckstrand \| Trauma-associated anterior cingulate connectivity during reward learning predicts affective and anxiety states in young adults. \| 2019 \| no ACE \| \| Eckstrand \| Persistent dose-dependent changes in brain structure in young adults with low-to-moderate alcohol exposure in utero. \| 2012 \| no significant interaction \| \| Edmiston \| Childhood maltreatment and response to novel face stimuli presented during functional magnetic resonance imaging in adults. \| 2013 \| no significant interaction \| \| Edmiston \| Corticostriatal-limbic gray matter morphology in adolescents with self-reported exposure to childhood maltreatment. \| 2011 \| structural MRI \| \| Ekblad \| Maternal smoking during pregnancy and regional brain volumes in preterm infants. \| 2010 \| no significant interaction \| \| El Marroun \| Prenatal exposure to maternal and paternal depressive symptoms and brain morphology: a population-based prospective neuroimaging study in young children. \| 2016 \| no whole brain \| \| El Marroun \| Prenatal Cannabis and Tobacco Exposure in Relation to Brain Morphology: A Prospective Neuroimaging Study in Young Children. \| 2016 \| structural MRI \| \| El Marroun \| Prenatal tobacco exposure and brain morphology: A prospective study in young children. \| 2014 \| structural MRI \| \| Ellman \| Structural brain alterations in schizophrenia following fetal exposure to the inflammatory cytokine interleukin-8. \| 2010 \| no whole brain \| \| Ellwood-Lowe \| Time-varying effects of income on hippocampal volume trajectories in adolescent girls. \| 2018 \| no whole brain \| \| Elsey \| Childhood trauma and neural responses to personalized stress, favorite-food and neutral-relaxing cues in adolescents. \| 2015 \| irrelevant domain \| \| Elton \| Effects of childhood maltreatment on the neural correlates of stress- and drug cue-induced cocaine craving. \| 2015 \| irrelevant domain \| \| Elton \| Childhood maltreatment is associated with a sex-dependent functional reorganization of a brain inhibitory control network. \| 2014 \| no significant interaction \| \| Engert \| Investigating the association between early life parental care and stress responsivity in adulthood. \| 2010 \| no whole brain \| \| Evans \| Childhood cumulative risk exposure and adult amygdala volume and function. \| 2016 \| no whole brain \| \| Everaerd \| Childhood abuse and deprivation are associated with distinct sex-dependent differences in brain morphology. \| 2016 \| structural MRI \| \| Everaerd \| Sex modulates the interactive effect of the serotonin transporter gene polymorphism and childhood adversity on hippocampal volume. \| 2012 \| structural MRI \| \| Faghiri \| Brain development includes linear and multiple nonlinear trajectories: a cross-sectional resting-state functional magnetic resonance imaging study. \| 2019 \| no significant interaction \| \| Fanti \| Amygdala functioning during threat acquisition and extinction differentiates antisocial subtypes. \| 2020 \| functional activity \| \| Farah \| Childhood poverty: Specific associations with neurocognitive development. \| 2006 \| no MRI \| \| Farrow \| Associations between early life stress and anterior pituitary gland volume development during late childhood. \| 2020 \| structural MRI \| \| Favaro \| Neural signatures of the interaction between the 5-HTTLPR genotype and stressful life events in healthy women. \| 2014 \| no ACE \| \| Favaro \| Effects of obstetric complications on volume and functional connectivity of striatum in anorexia nervosa patients. \| 2014 \| no whole brain \| \| Felmingham \| Neural responses to masked fear faces: sex differences and trauma exposure in posttraumatic stress disorder. \| 2010 \| no ACE \| \| Filippi \| Family history of alcohol use disorder is associated with brain structural and functional changes in healthy first-degree relatives. \| 2019 \| functional activity \| \| Finn \| Functional brain organization of working memory in adolescents varies in relation to family income and academic achievement. \| 2017 \| no whole brain \| \| Firat \| Putting race in context: social class modulates processing of race in the ventromedial prefrontal cortex and amygdala. \| 2017 \| no ACE \| \| Flanagan \| Effects of oxytocin on working memory and executive control system connectivity in posttraumatic stress disorder. \| 2018 \| no whole brain \| \| Fonzo \| Early life stress and the anxious brain: evidence for a neural mechanism linking childhood emotional maltreatment to anxiety in adulthood. \| 2016 \| no main effect \| \| Fonzo \| History of childhood maltreatment augments dorsolateral prefrontal processing of emotional valence in PTSD. \| 2016 \| no significant interaction \| \| Fonzo \| Neural functional and structural correlates of childhood maltreatment in women with intimate-partner violence-related posttraumatic stress disorder. \| 2013 \| structural MRI \| \| Ford \| Neurocognitive correlates of problem behavior in environmentally at-risk adolescents. \| 2007 \| FT not found \| \| Fourie \| Effects of early adversity and social discrimination on empathy for complex mental states: An fMRI investigation. \| 2019 \| functional activity \| \| Franke \| Premature brain aging in humans exposed to maternal nutrient restriction during early gestation. \| 2018 \| structural MRI \| \| Frewen \| Self-referential processing in women With PTSD: affective and neural response. \| 2011 \| no whole brain \| \| Frissen \| No evidence of association between childhood urban environment and cortical thinning in psychotic disorder. \| 2017 \| no whole brain \| \| Frissen \| Evidence that reduced gray matter volume in psychotic disorder is associated with exposure to environmental risk factors. \| 2018 \| structural MRI \| \| Fritz \| Mood and neural responses to social rejection do not seem to be altered in resilient adolescents with a history of adversity. \| 2020 \| functional activity \| \| Frodl \| BDNF Val66Met genotype interacts with childhood adversity and influences the formation of hippocampal subfields. \| 2014 \| no whole brain \| \| Frodl \| Childhood adversity impacts on brain subcortical structures relevant to depression. \| 2017 \| no whole brain \| \| Frodl \| Interaction of childhood stress with hippocampus and prefrontal cortex volume reduction in major depression. \| 2010 \| structural MRI \| \| Fryer \| Prenatal alcohol exposure affects frontal-striatal BOLD response during inhibitory control. \| 2007 \| functional activity \| \| Fryer \| Caudate volume predicts neurocognitive performance in youth with heavy prenatal alcohol exposure. \| 2012 \| no whole brain \| \| Fujisawa \| Neural basis of psychological growth following adverse experiences: a resting-state functional MRI study. \| 2015 \| no ACE \| \| Fujisawa \| Oxytocin receptor DNA methylation and alterations of brain volumes in maltreated children. \| 2019 \| structural MRI \| \| Fujisawa \| Type and timing of childhood maltreatment and reduced visual cortex volume in children and adolescents with reactive attachment disorder. \| 2018 \| structural MRI \| \| Gaffrey \| Amygdala reward reactivity mediates the association between preschool stress response and depression severity. \| 2018 \| no ACE \| \| Gale \| Association between exposure to air pollution and prefrontal cortical volume in adults: A cross-sectional study from the UK biobank. \| 2020 \| structural MRI \| \| Ganella \| Early life stress alters pituitary growth during adolescence-a longitudinal study. \| 2015 \| no whole brain \| \| Ganzel \| Stress and the healthy adolescent brain: evidence for the neural embedding of life events. \| 2013 \| functional activity \| \| Gard \| The long reach of early adversity: parenting, stress, and neural pathways to antisocial behavior in adulthood. \| 2017 \| no whole brain \| \| Garrett \| Brain activation to facial expressions in youth with PTSD symptoms. \| 2012 \| no main effect \| \| Gatt \| Impact of the HTR3A gene with early life trauma on emotional brain networks and depressed mood. \| 2010 \| no whole brain \| \| Gatt \| Interactions between BDNF Val66Met polymorphism and early life stress predict brain and arousal pathways to syndromal depression and anxiety. \| 2009 \| no whole brain \| \| Gautam \| Developmental Trajectories for Visuo-Spatial Attention are Altered by Prenatal Alcohol Exposure: A Longitudinal FMRI Study. \| 2015 \| functional activity \| \| Gautam \| Volume changes and brain-behavior relationships in white matter and subcortical gray matter in children with prenatal alcohol exposure. \| 2015 \| no whole brain \| \| Gautam \| Executive function and cortical thickness in youths prenatally exposed to cocaine, alcohol and tobacco. \| 2015 \| structural MRI \| \| Gee \| Early developmental emergence of human amygdala-prefrontal connectivity after maternal deprivation. \| 2013 \| functional activity \| \| Gee \| Maternal buffering of human amygdala-prefrontal circuitry during childhood but not during adolescence. \| 2014 \| no ACE \| \| Geller \| Effects of age, sex, and independent life events on amygdala and nucleus accumbens volumes in child bipolar I disorder. \| 2009 \| no whole brain \| \| Geng \| Long-term effects of prenatal drug exposure on the neural correlates of memory at encoding and retrieval. \| 2018 \| no significant interaction \| \| Gerin \| A neurocomputational investigation of reinforcement-based decision making as a candidate latent vulnerability mechanism in maltreated children. \| 2017 \| irrelevant domain \| \| Gerritsen \| Childhood maltreatment modifies the relationship of depression with hippocampal volume. \| 2015 \| no whole brain \| \| Gerritsen \| HPA axis genes, and their interaction with childhood maltreatment, are related to cortisol levels and stress-related phenotypes. \| 2017 \| no whole brain \| \| Gerritsen \| BDNF Val66Met genotype modulates the effect of childhood adversity on subgenual anterior cingulate cortex volume in healthy subjects. \| 2012 \| structural MRI \| \| Ghassabian \| Downstream effects of maternal hypothyroxinemia in early pregnancy: nonverbal IQ and brain morphology in school-age children. \| 2014 \| no significant interaction \| \| Ghersin \| Trends in the epidemiology of inflammatory bowel disease among Jewish Israeli adolescents: a population-based study. \| 2019 \| no MRI \| \| Gianaros \| Parental education predicts corticostriatal functionality in adulthood. \| 2011 \| functional activity \| \| Gianaros \| Potential neural embedding of parental social standing. \| 2008 \| no whole brain \| \| Glass \| Academic Difficulties in Children with Prenatal Alcohol Exposure: Presence, Profile, and Neural Correlates. \| 2017 \| no main effect \| \| Goetschius \| Childhood violence exposure and social deprivation predict adolescent amygdala-orbitofrontal cortex white matter connectivity. \| 2020 \| irrelevant measure \| \| Goff \| Reduced nucleus accumbens reactivity and adolescent depression following early-life stress. \| 2013 \| no whole brain \| \| Gold \| Childhood abuse and reduced cortical thickness in brain regions involved in emotional processing. \| 2016 \| no significant interaction \| \| Golde \| Healthy women with severe early life trauma show altered neural facilitation of emotion inhibition under acute stress \| 2020 \| functional activity \| \| Gollier-Briant \| Neural correlates of three types of negative life events during angry face processing in adolescents. \| 2016 \| no ACE \| \| Gonzalez \| Lower neighborhood quality in adolescence predicts higher mesolimbic sensitivity to reward anticipation in adulthood. \| 2016 \| functional activity \| \| Gonzalez \| Adolescent neighborhood quality predicts adult dACC response to social exclusion. \| 2014 \| irrelevant domain \| \| Gorka \| Reduced hippocampal and medial prefrontal gray matter mediate the association between reported childhood maltreatment and trait anxiety in adulthood and predict sensitivity to future life stress. \| 2014 \| no whole brain \| \| Grabe \| Effect of the interaction between childhood abuse and rs1360780 of the FKBP5 gene on gray matter volume in a general population sample. \| 2016 \| structural MRI \| \| Graham \| Maternal cortisol concentrations during pregnancy and sex-specific associations with neonatal amygdala connectivity and emerging internalizing behaviors. \| 2019 \| no main effect \| \| Graham \| Maternal Systemic Interleukin-6 During Pregnancy Is Associated With Newborn Amygdala Phenotypes and Subsequent Behavior at 2 Years of Age. \| 2018 \| no whole brain \| \| Grant \| Childhood trauma history differentiates amygdala response to sad faces within MDD. \| 2011 \| no ACE \| \| Grewen \| Prenatal cocaine effects on brain structure in early infancy. \| 2014 \| structural MRI \| \| Grimm \| Early life stress modulates oxytocin effects on limbic system during acute psychosocial stress. \| 2014 \| no whole brain \| \| Grohs \| Prenatal maternal and childhood bisphenol a exposure and brain structure and behavior of young children. \| 2019 \| no MRI \| \| Gross \| Neural correlates of verbal memory in youth with heavy prenatal alcohol exposure. \| 2018 \| no whole brain \| \| Gui \| Longitudinal study of neonatal brain tissue volumes in preterm infants and their ability to predict neurodevelopmental outcome. \| 2019 \| structural MRI \| \| Guyer \| Temperament and parenting styles in early childhood differentially influence neural response to peer evaluation in adolescence. \| 2015 \| no whole brain \| \| Habets \| Reduced cortical thickness as an outcome of differential sensitivity to environmental risks in schizophrenia. \| 2011 \| no whole brain \| \| Hackman \| Socioeconomic position and age-related disparities in regional cerebral blood flow within the prefrontal cortex. \| 2018 \| irrelevant domain \| \| Haghighi \| Prenatal exposure to maternal cigarette smoking, amygdala volume, and fat intake in adolescence. \| 2013 \| no significant interaction \| \| Hair \| Association of child poverty, brain development, and academic achievement. \| 2015 \| no whole brain \| \| Hanson \| Cumulative stress in childhood is associated with blunted reward-related brain activity in adulthood. \| 2016 \| functional activity \| \| Hanson \| Association between income and the hippocampus. \| 2011 \| no whole brain \| \| Hanson \| Behavioral problems after early life stress: contributions of the hippocampus and amygdala. \| 2015 \| no whole brain \| \| Hanson \| Blunted ventral striatum development in adolescence reflects emotional neglect and predicts depressive symptoms. \| 2015 \| no whole brain \| \| Hanson \| Family poverty affects the rate of human infant brain growth. \| 2013 \| structural MRI \| \| Hanson \| Structural variations in prefrontal cortex mediate the relationship between early childhood stress and spatial working memory. \| 2012 \| structural MRI \| \| Hanson \| Lower structural integrity of the uncinate fasciculus is associated with a history of child maltreatment and future psychological vulnerability to stress. \| 2015 \| structural connectivity \| \| Hardee \| Development of impulse control circuitry in children of alcoholics. \| 2014 \| no main effect \| \| Harms \| Early life stress, FK506 binding protein 5 gene (FKBP5) methylation, and inhibition-related prefrontal function: A prospective longitudinal study. \| 2017 \| functional activity \| \| Harms \| Instrumental learning and cognitive flexibility processes are impaired in children exposed to early life stress. \| 2018 \| irrelevant domain \| \| Harnett \| Negative life experiences contribute to racial differences in the neural response to threat. \| 2019 \| functional activity \| \| Hart \| Altered fear processing in adolescents with a history of severe childhood maltreatment: an fMRI study. \| 2018 \| functional activity \| \| Hasler \| Variations in brain morphometry among healthy preschoolers born preterm. \| 2020 \| structural MRI \| \| Hatchard \| Effects of low-level alcohol use on cognitive interference: an fMRI study in young adults. \| 2015 \| no ACE \| \| Haukvik \| Cerebral cortical thickness and a history of obstetric complications in schizophrenia. \| 2009 \| no significant interaction \| \| Heckendorf \| Neural processing of familiar and unfamiliar children’s faces: Effects of experienced love withdrawal, but no effects of neutral and threatening priming. \| 2016 \| irrelevant domain \| \| Hedderich \| Aberrant gyrification contributes to the link between gestational age and adult IQ after premature birth. \| 2019 \| structural MRI \| \| Hedderich \| An analysis of MRI derived cortical complexity in premature-born adults: Regional patterns, risk factors, and potential significance. \| 2020 \| structural MRI \| \| Heim \| Decreased cortical representation of genital somatosensory field after childhood sexual abuse. \| 2013 \| structural MRI \| \| Heitzeg \| Left middle frontal gyrus response to inhibitory errors in children prospectively predicts early problem substance use. \| 2014 \| no ACE \| \| Hendrickson \| Cortical gyrification is abnormal in children with prenatal alcohol exposure. \| 2017 \| no significant interaction \| \| Hendrickson \| Two-year cortical trajectories are abnormal in children and adolescents with prenatal alcohol exposure. \| 2018 \| no significant interaction \| \| Hentze \| Functional correlates of childhood maltreatment and symptom severity during affective theory of mind tasks in chronic depression. \| 2006 \| irrelevant domain \| \| Hernaus \| Brain-derived neurotrophic factor/FK506-binding protein 5 genotype by childhood trauma interactions do not impact on hippocampal volume and cognitive performance. \| 2014 \| no whole brain \| \| Herringa \| Childhood and adult trauma both correlate with dorsal anterior cingulate activation to threat in combat veterans. \| 2013 \| functional activity \| \| Herzog \| Increased recruitment of cognitive control in the presence of traumatic stimuli in complex PTSD. \| 2017 \| no significant interaction \| \| Herzog \| Influence of severity of type and timing of retrospectively reported childhood maltreatment on female amygdala and hippocampal volume. \| 2020 \| structural MRI \| \| Heyn \| Longitudinal cortical markers of persistence and remission of pediatric PTSD \| 2019 \| structural MRI \| \| Hill \| Cerebellum volume in high-risk offspring from multiplex alcohol dependence families: Association with allelic variation in GABRA2 and BDNF. \| 2011 \| no whole brain \| \| Hill \| Disruption of orbitofrontal cortex laterality in offspring from multiplex alcohol dependence families. \| 2009 \| no whole brain \| \| Hill \| Right amygdala volume in adolescent and young adult offspring from families at high risk for developing alcoholism. \| 2001 \| no whole brain \| \| Hirjak \| Cortical folding patterns are associated with impulsivity in healthy young adults. \| 2017 \| no ACE \| \| Hoare \| Accelerated epigenetic aging in adolescents from low-income households is associated with altered development of brain structures. \| 2020 \| structural MRI \| \| Hodel \| Duration of early adversity and structural brain development in post-institutionalized adolescents. \| 2015 \| no whole brain \| \| Hoffmann \| Risk-taking, peer-influence and child maltreatment: a neurocognitive investigation. \| 2018 \| irrelevant domain \| \| Holmes \| Parenting and salience network connectivity among african americans: a protective pathway for health-risk behaviors. \| 2018 \| no ACE \| \| Holmes \| Peer influence, Frontostriatal connectivity, and delay discounting in African American emerging adults \| 2020 \| no ACE \| \| Holz \| Effect of prenatal exposure to tobacco smoke on inhibitory control: neuroimaging results from a 25-year prospective study. \| 2014 \| functional activity \| \| Holz \| Evidence for a sex-dependent MAOA× childhood stress interaction in the neural circuitry of aggression. \| 2016 \| functional activity \| \| Holz \| Ventral striatum and amygdala activity as convergence sites for early adversity and conduct disorder. \| 2017 \| functional activity \| \| Holz \| Role of FKBP5 in emotion processing: results on amygdala activity, connectivity and volume \| 2015 \| no main effect \| \| Holz \| The long-term impact of early life poverty on orbitofrontal cortex volume in adulthood: results from a prospective study over 25 years. \| 2015 \| structural MRI \| \| Hoy \| Childhood trauma and hippocampal and amygdalar volumes in first-episode psychosis. \| 2012 \| no whole brain \| \| Hu \| Social status modulates the neural response to unfairness. \| 2015 \| no ACE \| \| Hunt \| Association of neighborhood-level disadvantage with cerebral and hippocampal volume \| 2019 \| structural MRI \| \| Huntley \| Adolescent substance use and functional connectivity between the ventral striatum and hippocampus \| 2020 \| no ACE \| \| Hurt \| Children with and without gestational cocaine exposure: A neurocognitive systems analysis. \| 2009 \| no MRI \| \| Hurt \| Functional magnetic resonance imaging and working memory in adolescents with gestational cocaine exposure. \| 2008 \| no whole brain \| \| Ikonomidou \| Brain morphology alterations in the basal ganglia and the hypothalamus following prenatal exposure to antiepileptic drugs. \| 2007 \| no significant interaction \| \| Infante \| Atypical cortical gyrification in adolescents with histories of heavy prenatal alcohol exposure. \| 2015 \| irrelevant domain \| \| Inkelis \| Neurodevelopment in adolescents and adults with fetal alcohol spectrum disorders (FASD): A magnetic resonance region of interest analysis. \| 2020 \| structural MRI \| \| Ivanovic \| Long-term effects of severe undernutrition during the first year of life on brain development and learning in Chilean high-school graduates. \| 2000 \| structural MRI \| \| Jacobsen \| Gender-specific effects of prenatal and adolescent exposure to tobacco smoke on auditory and visual attention. \| 2007 \| irrelevant domain \| \| Jacobsen \| Heavy Prenatal Alcohol Exposure is Related to Smaller Corpus Callosum in Newborn MRI Scans. \| 2017 \| no significant interaction \| \| Jacobsen \| Allelic variation of calsyntenin 2 (CLSTN2) modulates the impact of developmental tobacco smoke exposure on mnemonic processing in adolescents. \| 2009 \| no whole brain \| \| Jacobsen \| Visuospatial memory deficits emerging during nicotine withdrawal in adolescents with prenatal exposure to active maternal smoking. \| 2006 \| no whole brain \| \| Janiri \| Amygdala and hippocampus volumes are differently affected by childhood trauma in patients with bipolar disorders and healthy controls. \| 2017 \| no whole brain \| \| Jankowski \| Preliminary evidence of the impact of early childhood maltreatment and a preventive intervention on neural patterns of response inhibition in early adolescence. \| 2017 \| no main effect \| \| Janulewicz \| Structural Magnetic Resonance Imaging in an adult cohort following prenatal and early postnatal exposure to tetrachloroethylene (PCE)-contaminated drinking water. \| 2013 \| no significant interaction \| \| Jatzko \| Cerebral lesions at fetal magnetic resonance imaging and neurologic outcome after single fetal death in monochorionic twins. \| 2015 \| irrelevant domain \| \| Javanbakht \| Childhood poverty predicts adult amygdala and frontal activity and connectivity in response to emotional faces. \| 2015 \| no whole brain \| \| Javanbakht \| Sex-specific effects of childhood poverty on neurocircuitry of processing of emotional cues: a neuroimaging study \| 2016 \| no whole brain \| \| Jaworska \| A preliminary study of the influence of age of onset and childhood trauma on cortical thickness in major depressive disorder. \| 2014 \| structural MRI \| \| Jedd \| Long-term consequences of childhood maltreatment: Altered amygdala functional connectivity. \| 2015 \| functional activity \| \| Jednoróg \| The influence of socioeconomic status on children’s brain structure. \| 2012 \| structural MRI \| \| Jenkins \| Subcortical structural variations associated with low socioeconomic status in adolescents \| 2020 \| structural MRI \| \| Jenness \| Alterations in neural circuits underlying emotion regulation following child maltreatment: a mechanism underlying trauma-related psychopathology \| 2020 \| functional activity \| \| Jensen \| Effect of early adversity and childhood internalizing symptoms on brain structure in young men. \| 2015 \| no whole brain \| \| Jha \| Antenatal depression, treatment with selective serotonin reuptake inhibitors, and neonatal brain structure: A propensity-matched cohort study. \| 2016 \| no significant interaction \| \| Jin \| A voxel-based morphometric MRI study in young adults with borderline personality disorder. \| 2016 \| no significant interaction \| \| Johnson \| Effects of stressful life events on cerebral white matter hyperintensity progression. \| 2017 \| no ACE \| \| Jones \| Exploring emotion regulation in juveniles who have sexually offended: An fMRI study. \| 2018 \| functional activity \| \| Joseph \| Neural correlates of oxytocin and cue reactivity in cocaine-dependent men and women with and without childhood trauma. \| 2020 \| functional activity \| \| Joseph \| Oxytocin-induced changes in intrinsic network connectivity in cocaine use disorder: modulation by gender, childhood trauma, and years of use. \| 2019 \| no main effect \| \| Joss \| Effects of a mindfulness based behavioral intervention for young adults with childhood maltreatment history on hippocampal morphometry: a pilot MRI study with voxel-based morphometry \| 2020 \| structural MRI \| \| Juhasz \| The CREB1-BDNF-NTRK2 pathway in depression: multiple gene-cognition-environment interactions. \| 2011 \| no main effect \| \| Kaag \| Enhanced amygdala-striatal functional connectivity during the processing of cocaine cues in male cocaine users with a history of childhood trauma. \| 2018 \| irrelevant domain \| \| Kaess \| Childhood maltreatment, pituitary volume and adolescent hypothalamic-pituitary-adrenal axis – Evidence for a maltreatment-related attenuation. \| 2018 \| no whole brain \| \| Kahila \| Brain magnetic resonance imaging of infants exposed prenatally to buprenorphine. \| 2007 \| irrelevant domain \| \| Kamkar \| Ventral striatal activity links adversity and reward processing in children. \| 2017 \| no whole brain \| \| Kamps \| Connectivity at the origins of domain specificity in the cortical face and place networks. \| 2020 \| no ACE \| \| Kapetanovic \| Effect of HIV and Interpersonal Trauma on Cortical Thickness, Cognition, and Daily Functioning. \| 2020 \| structural MRI \| \| Karcher \| Environmental Risk Factors and Psychotic-Like Symptoms in Children Aged 9-11. \| 2020 \| structural MRI \| \| Keding \| Paradoxical prefrontal-amygdala recruitment to angry and happy expressions in pediatric posttraumatic stress disorder. \| 2016 \| functional activity \| \| Keding \| Abnormal structure of fear circuitry in pediatric post-traumatic stress disorder. \| 2015 \| structural MRI \| \| Kelly \| Cortical thickness, surface area, and gyrification abnormalities in children exposed to maltreatment: neural markers of vulnerability? \| 2013 \| structural MRI \| \| Kelly \| The sexually dimorphic impact of maltreatment on cortical thickness, surface area and gyrification. \| 2016 \| structural MRI \| \| Khundrakpam \| Distinct influence of parental occupation on cortical thickness and surface area in children and adolescents: Relation to self-esteem. \| 2020 \| structural MRI \| \| Kim \| Association between posttraumatic stress disorder severity and amygdala habituation to fearful stimuli. \| 2019 \| functional activity \| \| Kim \| Effects of childhood poverty and chronic stress on emotion regulatory brain function in adulthood. \| 2013 \| functional activity \| \| Kim \| Childhood social inequalities influences neural processes in young adult caregiving. \| 2015 \| irrelevant domain \| \| Kim \| Alexithymia and frontal-amygdala functional connectivity in North Korean refugees. \| 2020 \| no ACE \| \| Kim \| A link between childhood adversity and trait anger reflects relative activity of the amygdala and dorsolateral prefrontal cortex. \| 2018 \| no whole brain \| \| Kim \| Childhood poverty and the organization of structural brain connectome. \| 2019 \| structural MRI \| \| Kim \| Prenatal Maternal Cortisol Has Sex-Specific Associations with Child Brain Network Properties. \| 2017 \| structural connectivity \| \| Kitayama \| Morphologic alterations in the corpus callosum in abuse-related posttraumatic stress disorder: a preliminary study. \| 2007 \| no whole brain \| \| Kitayama \| Smaller volume of anterior cingulate cortex in abuse-related posttraumatic stress disorder. \| 2006 \| no whole brain \| \| Klabunde \| The moderating effects of sex on insula subdivision structure in youth with posttraumatic stress symptoms. \| 2017 \| no whole brain \| \| Kline \| Early cortical maturation predicts neurodevelopment in very preterm infants. \| 2019 \| structural MRI \| \| Klucken \| Individual differences in neural correlates of fear conditioning as a function of 5-HTTLPR and stressful life events. \| 2013 \| no ACE \| \| Kneer \| Serotonergic influence on depressive symptoms and trait anxiety is mediated by negative life events and frontal activation in children and adolescents. \| 2020 \| functional activity \| \| Knickmeyer \| Rate of Chiari I Malformation in Children of Mothers with Depression with and without Prenatal SSRI Exposure. \| 2014 \| no whole brain \| \| Knickmeyer \| Impact of demographic and obstetric factors on infant brain volumes: a population neuroscience study. \| 2017 \| structural MRI \| \| Koc \| Investigation of structure-function correlation among the young offspring of patients with bipolar disorder. \| 2020 \| structural MRI \| \| Kodali \| Differential Recruitment of Brain Regions During Response Inhibition in Children Prenatally Exposed to Alcohol. \| 2017 \| functional activity \| \| Koelkebeck \| Gray matter volume reductions in patients with schizophrenia: A replication study across two cultural backgrounds. \| 2019 \| no ACE \| \| Kok \| Normal variation in early parental sensitivity predicts child structural brain development. \| 2015 \| no ACE \| \| Kolla \| Disentangling possible effects of childhood physical abuse on gray matter changes in violent offenders with psychopathy. \| 2014 \| structural MRI \| \| Korgaonkar \| Early exposure to traumatic stressors impairs emotional brain circuitry. \| 2013 \| no whole brain \| \| Krautheim \| Outgroup emotion processing in the vACC is modulated by childhood trauma and CACNA1C risk variant. \| 2018 \| no whole brain \| \| Krishnadas \| Socioeconomic deprivation and cortical morphology: psychological, social, and biological determinants of ill health study. \| 2013 \| no whole brain \| \| Krishnadas \| The envirome and the connectome: exploring the structural noise in the human brain associated with socioeconomic deprivation. \| 2013 \| structural MRI \| \| Kronmüller \| Life events and hippocampal volume in first-episode major depression. \| 2008 \| no ACE \| \| Krueger \| Para-limbic Structural Abnormalities Are Associated With Internalizing Symptoms in Children With Prenatal Alcohol Exposure. \| 2020 \| structural MRI \| \| Kuban \| Association of circulating proinflammatory and anti-inflammatory protein biomarkers in extremely preterm born children with subsequent brain magnetic resonance imaging volumes and cognitive function at age 10 years. \| 2019 \| structural MRI \| \| Kuhn \| Mismatch or allostatic load? Timing of life adversity differentially shapes gray matter volume and anxious temperament. \| 2016 \| structural MRI \| \| Kühn \| From mother to child: Orbitofrontal cortex gyrification and changes of drinking behaviour during adolescence. \| 2016 \| irrelevant domain \| \| Kumari \| Lower anterior cingulate volume in seriously violent men with antisocial personality disorder or schizophrenia and a history of childhood abuse. \| 2014 \| no whole brain \| \| Kumari \| Reduced thalamic volume in men with antisocial personality disorder or schizophrenia and a history of serious violence and childhood abuse. \| 2013 \| structural MRI \| \| Lambert \| Altered development of hippocampus-dependent associative learning following early-life adversity. \| 2019 \| no significant interaction \| \| Lambert \| Hippocampal contribution to context encoding across development Is disrupted following early-life adversity. \| 2017 \| no whole brain \| \| Lammeyer \| Evidence of brain network aberration in healthy subjects with urban upbringing—A multimodal DTI and VBM study. \| 2019 \| structural MRI \| \| Landre \| Working memory processing of traumatic material in women with posttraumatic stress disorder. \| 2012 \| no ACE \| \| Landré \| Preserved subcortical volumes and cortical thickness in women with sexual abuse-related PTSD \| 2010 \| structural MRI \| \| Lang \| Cognitive reappraisal in trauma-exposed women with borderline personality disorder. \| 2012 \| functional activity \| \| Lanius \| Brain activation during script-driven imagery induced dissociative responses in PTSD: a functional magnetic resonance imaging investigation. \| 2002 \| irrelevant domain \| \| Lansing \| Cumulative trauma, adversity and grief symptoms associated with fronto-temporal regions in life-course persistent delinquent boys. \| 2016 \| structural MRI \| \| Lawson \| Associations between children’s socioeconomic status and prefrontal cortical thickness. \| 2013 \| no whole brain \| \| Lebel \| A longitudinal study of the long-term consequences of drinking during pregnancy: heavy in utero alcohol exposure disrupts the normal processes of brain development. \| 2012 \| irrelevant domain \| \| Lebel \| Prepartum and Postpartum Maternal Depressive Symptoms Are Related to Children’s Brain Structure in Preschool \| 2016 \| structural MRI \| \| Lederbogen \| City living and urban upbringing affect neural social stress processing in humans. \| 2011 \| no experimental article \| \| Lee \| Aberrant function of frontoamygdala circuits in adolescents with previous verbal abuse experiences. \| 2015 \| functional activity \| \| Lee \| Altered function of ventrolateral prefrontal cortex in adolescents with peer verbal abuse history. \| 2017 \| functional activity \| \| Lee \| The impact of prenatal and neonatal infection on neurodevelopmental outcomes in very preterm infants. \| 2014 \| irrelevant domain \| \| Lee \| Hippocampal subfields volume reduction in high schoolers with previous verbal abuse experiences. \| 2018 \| no whole brain \| \| Leicht-Deobald \| Work-related social support modulates effects of early life stress on limbic reactivity during stress. \| 2018 \| functional activity \| \| Lenze \| Childhood adversity predicts earlier onset of major depression but not reduced hippocampal volume. \| 2008 \| no whole brain \| \| Levesque \| The impact of the in utero and early postnatal environments on grey and white matter volume: a study with adolescent monozygotic twins. \| 2015 \| structural MRI \| \| Li \| Occipital-temporal reduction and Sustained Visual attention deficit in prenatal alcohol exposed adults. \| 2008 \| irrelevant domain \| \| Li \| Longitudinal changes of amygdala functional connectivity in adolescents prenatally exposed to cocaine \| 2019 \| no main effect \| \| Li \| Increased “default mode” activity in adolescents prenatally exposed to cocaine. \| 2011 \| no whole brain \| \| Li \| Longitudinal changes of amygdala and default mode activation in adolescents prenatally exposed to cocaine. \| 2016 \| no whole brain \| \| Li \| Prenatal cocaine exposure alters emotional arousal regulation and its effects on working memory. \| 2009 \| no whole brain \| \| Li \| Prenatal cocaine exposure alters functional activation in the ventral prefrontal cortex and its structural connectivity with the amygdala. \| 2013 \| no whole brain \| \| Liao \| Childhood maltreatment is associated with larger left thalamic gray matter volume in adolescents with generalized anxiety disorder. \| 2013 \| structural MRI \| \| Liberzon \| Childhood poverty and recruitment of adult emotion regulatory neurocircuitry. \| 2014 \| functional activity \| \| Lichtin \| Material hardship, prefrontal cortex-amygdala structure, and internalizing symptoms in children \| 2020 \| structural MRI \| \| Lieslehto \| Early adversity and brain response to faces in young adulthood. \| 2017 \| no whole brain \| \| Lim \| Neural correlates of error processing in young people qith a history of severe childhood abuse: an fMRI study. \| 2015 \| functional activity \| \| Lim \| Neurofunctional abnormalities during sustained attention in severe childhood abuse. \| 2016 \| irrelevant domain \| \| Lin \| Characterizing intrinsic functional connectivity in relation to impaired self-regulation in intellectually able male youth with autism spectrum disorder \| 2020 \| no ACE \| \| Lindgren \| Longitudinal evidence for smaller hippocampus volume as a vulnerability factor for perceived stress. \| 2016 \| no ACE \| \| Lischinsky \| Preliminary findings show maternal hypothyroidism may contribute to abnormal cortical morphology in offspring. \| 2016 \| structural MRI \| \| Little \| Linking the serotonin transporter gene, family environments, hippocampal volume and depression onset: A prospective imaging gene X environment analysis. \| 2015 \| no whole brain \| \| Little \| Multivariate models of brain volume for identification of children and adolescents with fetal alcohol spectrum disorder. \| 2019 \| structural MRI \| \| Liu \| Regional brain morphometry and impulsivity in adolescents following prenatal exposure to cocaine and tobacco. \| 2013 \| no whole brain \| \| Lombardo \| Fetal programming effects of testosterone on the reward system and behavioral approach tendencies in humans. \| 2012 \| no whole brain \| \| Long \| The brain’s functional connectome in young children with prenatal alcohol exposure. \| 2019 \| no significant interaction \| \| Longo \| The long-term effects of prenatal nicotine exposure on response inhibition: an fMRI study of young adults. \| 2013 \| functional activity \| \| Longo \| The long-term effects of prenatal nicotine exposure on verbal working memory: An fMRI study of young adults. \| 2014 \| functional activity \| \| Lotfipour \| Orbitofrontal cortex and drug use during adolescence: role of prenatal exposure to maternal smoking and BDNF genotype. \| 2009 \| no whole brain \| \| Loth \| Oxytocin receptor genotype modulates ventral striatal activity to social cues and response to stressful life events. \| 2014 \| no main effect \| \| Lu \| Effects of prenatal methamphetamine exposure on verbal memory revealed with functional magnetic resonance imaging. \| 2009 \| irrelevant domain \| \| Lu \| Reduced cingulate gyrus volume associated with enhanced cortisol awakening response in young healthy adults reporting childhood trauma. \| 2013 \| structural MRI \| \| Luby \| Association between early life adversity and risk for poor emotional and physical health in adolescence: a putative mechanistic neurodevelopmental pathway. \| 2017 \| no whole brain \| \| Luby \| Association of timing of adverse childhood experiences and caregiver support with regionally specific brain development in adolescents. \| 2019 \| structural MRI \| \| Luby \| The effects of poverty on childhood brain development: the mediating effect of caregiving and stressful life events. \| 2013 \| structural MRI \| \| Lugo-Candelas \| Associations between brain structure and connectivity in infants and exposure to selective serotonin reuptake inhibitors during pregnancy. \| 2018 \| structural MRI \| \| Luo \| Association between childhood trauma and risk for obesity: a putative neurocognitive developmental pathway. \| 2020 \| structural MRI \| \| Luoni \| Ankyrin-3 as a molecular marker of early-life stress and vulnerability to psychiatric disorders. \| 2016 \| no whole brain \| \| Lv \| Assessing effects of prenatal alcohol exposure using group-wise sparse representation of fMRI data. \| 2015 \| irrelevant domain \| \| Ly \| Subjective socioeconomic status predicts human ventral striatal responses to social status information. \| 2011 \| no ACE \| \| Lyons-Ruth \| Disorganized attachment in infancy predicts greater amygdala volume in adulthood. \| 2016 \| no whole brain \| \| Ma \| Neural responses to perceived pain in others predict real-life monetary donations in different socioeconomic contexts. \| 2011 \| no ACE \| \| Machado \| Interaction between perceived maternal care, anxiety symptoms, and the neurobehavioral response to palatable foods in adolescents. \| 2016 \| irrelevant domain \| \| Mackes \| Early childhood deprivation is associated with alterations in adult brain structure despite subsequent environmental enrichment. \| 2020 \| structural MRI \| \| Maheu \| A preliminary study of medial temporal lobe function in youths with a history of caregiver deprivation and emotional neglect. \| 2010 \| no whole brain \| \| Maier \| Association of childhood maltreatment with interpersonal distance and social touch preferences in adulthood. \| 2020 \| functional activity \| \| Malisza \| Comparison of spatial working memory in children with prenatal alcohol exposure and those diagnosed with ADHD; A functional magnetic resonance imaging study. \| 2012 \| functional activity \| \| Malter Cohen \| Early-life stress has persistent effects on amygdala function and development I mice and humans \| 2013 \| functional activity \| \| Malykhin \| Fronto-limbic volumetric changes in major depressive disorder. \| 2012 \| no whole brain \| \| Mareckova \| Temporally and sex-specific effects of maternal perinatal stress on offspring cortical gyrification and mood in young adulthood. \| 2020 \| structural MRI \| \| Marecková \| Identifying craniofacial features associated with prenatal exposure to androgens and testing their relationship with brain development. \| 2015 \| no significant interaction \| \| Marecková \| Perinatal stress and human hippocampal volume: Findings from typically developing young adults. \| 2018 \| no whole brain \| \| Maria \| Relationship between maternal pregnancy-related anxiety and infant brain responses to emotional speech - a pilot study. \| 2020 \| no MRI \| \| Marshall \| Deficient inhibitory control as an outcome of childhood trauma. \| 2016 \| no MRI \| \| Marusak \| Childhood trauma exposure disrupts the automatic regulation of emotional processing. \| 2015 \| functional activity \| \| Marusak \| Disrupted insula-based neural circuit organization and conflict interference in trauma-exposed youth. \| 2015 \| functional activity \| \| Marusak \| Amygdala responses to salient social cues vary with oxytocin receptor genotype in youth. \| 2015 \| no main effect \| \| Marusak \| Interactive effects of BDNF Val66Met genotype and trauma on limbic brain anatomy in childhood. \| 2016 \| no main effect \| \| Mattan \| External motivation to avoid prejudice alters neural responses to targets varying in race and status. \| 2018 \| no ACE \| \| Matthews \| Longitudinal preterm cerebellar volume: perinatal and neurodevelopmental outcome associations. \| 2018 \| structural MRI \| \| McCall \| Hippocampal volumetry and episodic memory in preterm born children. \| 2020 \| no experimental article \| \| McCrory \| Amygdala activation in maltreated children during pre-attentive emotional processing. \| 2013 \| functional activity \| \| McCrory \| Autobiographical memory: a candidate latent vulnerability mechanism for psychiatric disorder following childhood maltreatment. \| 2017 \| irrelevant domain \| \| McCrory \| Heightened neural reactivity to threat in child victims of family violence. \| 2011 \| no experimental article \| \| McLachlan \| Current Socioeconomic Status Correlates With Brain Volumes in Healthy Children and Adolescents but Not in Children With Prenatal Alcohol Exposure \| 2020 \| structural MRI \| \| McLaughlin \| Child maltreatment and neural systems underlying emotion regulation. \| 2015 \| functional activity \| \| McLaughlin \| Maltreatment exposure, brain structure, and fear conditioning in children and adolescents. \| 2016 \| no whole brain \| \| McLaughlin \| Widespread reductions in cortical thickness following severe early-life deprivation: A neurodevelopmental pathway to attention-deficit/hyperactivity disorder. \| 2014 \| no whole brain \| \| McQuaid \| Altered cortical structure and psychiatric symptom risk in adolescents exposed to maternal stress in utero: A retrospective investigation. \| 2019 \| structural MRI \| \| Mehta \| Hyporesponsive reward anticipation in the basal ganglia following severe institutional deprivation early in life. \| 2010 \| no significant interaction \| \| Mehta \| Amygdala, hippocampal and corpus callosum size following severe early institutional deprivation: the English and Romanian Adoptees study pilot. \| 2009 \| no whole brain \| \| Meintjes \| An fMRI study of number processing in children with fetal alcohol syndrome. \| 2010 \| irrelevant domain \| \| Merlini \| Mid-hindbrain malformations due to drugs taken during pregnancy. \| 2014 \| no experimental article \| \| Merz \| Socioeconomic status, amygdala volume, and internalizing symptoms in children and adolescents. \| 2018 \| no whole brain \| \| Merz \| Parental punitive discipline and children’s depressive symptoms: Associations with striatal volume. \| 2019 \| structural MRI \| \| Merz \| Socioeconomic disparities in chronic physiologic stress are associated with brain structure in children \| 2019 \| structural MRI \| \| Metz \| The influence of early life stress on the integration of emotion and working memory. \| 2018 \| no whole brain \| \| Mielke \| Alterations of brain volumes in women with early life maltreatment and their associations with oxytocin. \| 2018 \| structural MRI \| \| Mielke \| Maternal sensitivity and the empathic brain: Influences of early life maltreatment. \| 2016 \| structural MRI \| \| Migliorini \| Anterior cingulate cortex surface area relates to behavioral inhibition in adolescents with and without heavy prenatal alcohol exposure. \| 2015 \| no whole brain \| \| Miller \| Cognition-childhood maltreatment interactions in the prediction of antidepressant outcomes in Major Depressive Disorder patients: results from the iSPOT-D trial. \| 2015 \| functional activity \| \| Miller \| Functional connectivity in central executive network protects youth against cardiometabolic risks linked with neighborhood violence. \| 2018 \| no main effect \| \| Modi \| The effects of repeated antenatal glucocorticoid therapy on the developing brain. \| 2001 \| no significant interaction \| \| Molendijk \| BDNF val 66met affects hippocampal volume and emotion-related hippocampal memory activity. \| 2012 \| irrelevant domain \| \| Monk \| Amygdala and nucleus accumbens activation to emotional facial expressions in children and adolescents at risk for major depression. \| 2008 \| no whole brain \| \| Monteleone \| The effects of childhood maltreatment on brain structure in adults with eating disorders. \| 2019 \| structural MRI \| \| Moog \| Intergenerational effect of maternal exposure to childhood maltreatment on newborn brain anatomy. \| 2018 \| no ACE \| \| Moore \| Pituitary lacks sexual dimorphism and displays reduced signal intensity on T1-weighted MRI in adolescents with histories of heavy prenatal alcohol exposure. \| 2016 \| structural MRI \| \| Morandotti \| Childhood abuse is associated with structural impairment in the ventrolateral prefrontal cortex and aggressiveness in patients with borderline personality disorder. \| 2013 \| no whole brain \| \| Morey \| Amygdala, hippocampus, and ventral medial prefrontal cortex volumes differ in maltreated youth with and without chronic posttraumatic stress disorder. \| 2016 \| no significant interaction \| \| Morey \| Genetic predictors of hippocampal subfield volume in PTSD cases and trauma-exposed controls. \| 2020 \| structural MRI \| \| Morgan \| Maternal depression and warmth during childhood predict age 20 neural response to reward. \| 2014 \| no whole brain \| \| Morris \| Elevated brain oxygen extraction fraction in preterm newborns with anemia measured using noninvasive MRI. \| 2018 \| structural MRI \| \| Mortamais \| Effects of prenatal exposure to particulate matter air pollution on corpus callosum and behavioral problems in children. \| 2019 \| structural MRI \| \| Morton \| Maternal dietary intake of omega-3 fatty acids correlates positively with regional brain volumes in 1-month-old term infants \| 2020 \| structural MRI \| \| Moser \| Parental reflective functioning correlates to brain activation in response to video-stimuli of mother–child dyads: Links to maternal trauma history and PTSD. \| 2019 \| functional activity \| \| Mueller \| Early-life stress is associated with impairment in cognitive control in adolescence: an fMRI study. \| 2010 \| functional activity \| \| Muetzel \| Frequent bullying involvement and brain morphology in children. \| 2019 \| structural MRI \| \| Müller \| Altered reward processing in adolescents with prenatal exposure to maternal cigarette smoking. \| 2013 \| functional activity \| \| Mullins \| Neighborhood Deprivation Shapes Motivational-Neurocircuit Recruitment in Children. \| 2020 \| functional activity \| \| Murray \| Early life socioeconomic circumstance and late life brain hyperintensities--a population based cohort study. \| 2014 \| irrelevant domain \| \| Muscatell \| Social status modulates neural activity in the mentalizing network. \| 2012 \| no whole brain \| \| Mutluer \| Lateralization of neurobiological response in adolescents with post-traumatic stress disorder related to severe childhood sexual abuse: the Tri-Modal Reaction (T-MR) model of protection. \| 2018 \| no whole brain \| \| Nardelli \| Extensive Deep Gray Matter Volume Reductions in Children and Adolescents with Fetal Alcohol Spectrum Disorders. \| 2011 \| structural MRI \| \| Nassar \| Gestational age is dimensionally associated with structural brain network abnormalities across development. \| 2019 \| structural MRI \| \| Neukel \| The maternal brain in women with a history of early-life maltreatment: An imagination-based fMRI study of conflictual versus pleasant interactions with children. \| 2018 \| irrelevant domain \| \| Nicol \| Childhood trauma, midbrain activation and psychotic symptoms in borderline personality disorder. \| 2015 \| functional activity \| \| Nikolova \| Shifting priorities: highly conserved behavioral and brain network adaptations to chronic stress across species. \| 2018 \| no whole brain \| \| Noble \| Brain-behavior relationships in reading acquisition are modulated by socioeconomic factors. \| 2006 \| no MRI \| \| Noble \| Neurocognitive correlates of socioeconomic status in kindergarten children. \| 2005 \| no MRI \| \| Noble \| Socioeconomic gradients predict individual differences in neurocognitive abilities. \| 2007 \| no MRI \| \| Noble \| Hippocampal volume varies with educational attainment across the life-span. \| 2012 \| no whole brain \| \| Noble \| Family income, parental education and brain structure in children and adolescents. \| 2015 \| structural MRI \| \| Nogovitsyn \| Childhood trauma and amygdala nuclei volumes in youth at risk for mental illness \| 2020 \| structural MRI \| \| Noll-Hussong \| Aftermath of sexual abuse history on adult patients suffering from chronic functional pain syndromes: An fMRI pilot study. \| 2010 \| irrelevant domain \| \| Norman \| A functional magnetic resonance imaging study of spatial working memory in children with prenatal alcohol exposure: contribution of familial history of alcohol use disorders. \| 2013 \| functional activity \| \| Nwulia \| A pilot study of reduced olfactory bulb volume as a marker of PTSD in childhood trauma-exposed adult HIV-infected patients. \| 2017 \| no whole brain \| \| Nygaard \| Neuroanatomical characteristics of youths with prenatal opioid and poly-drug exposure. \| 2018 \| structural MRI \| \| O’Brien \| Effect of predictive cuing on response inhibition in children with heavy prenatal alcohol exposure. \| 2013 \| functional activity \| \| O’Hare \| Altered frontal-parietal functioning during verbal working memory in children and adolescents with heavy prenatal alcohol exposure. \| 2009 \| functional activity \| \| O’Hare \| Mapping cerebellar vermal morphology and cognitive correlates in prenatal alcohol exposure. \| 2005 \| no whole brain \| \| Opel \| Hippocampal atrophy in major depression: a function of childhood maltreatment rather than diagnosis? \| 2014 \| no whole brain \| \| Opel \| Differing brain structural correlates of familial and environmental risk for major depressive disorder revealed by a combined VBM/pattern recognition approach. \| 2016 \| structural MRI \| \| Opmeer \| Interaction of neuropeptide Y genotype and childhood emotional maltreatment on brain activity during emotional processing. \| 2014 \| no main effect \| \| Oshri \| Adverse childhood experiences and amygdalar reduction: high-resolution segmentation reveals associations with subnuclei and psychiatric outcomes. \| 2019 \| structural MRI \| \| Oswald \| History of childhood adversity is positively associated with ventral striatal dopamine responses to amphetamine. \| 2014 \| no MRI \| \| Pagliaccio \| Amygdala functional connectivity, HPA axis genetic variation, and life stress in children and relations to anxiety and emotion regulation. \| 2015 \| no whole brain \| \| Pagliaccio \| Stress-system genes and life stress predict cortisol levels and amygdala and hippocampal volumes in children. \| 2014 \| no whole brain \| \| Papagni \| Effects of stressful life events on human brain structure: a longitudinal voxel-based morphometry study. \| 2011 \| no ACE \| \| Paquola \| Hippocampal development in youth with a history of childhood maltreatment. \| 2017 \| no whole brain \| \| Pascoe \| Efficiency of structural connectivity networks relates to intrinsic motivation in children born extremely preterm. \| 2019 \| structural MRI \| \| Paus \| KCTD8 gene and brain growth in adverse intrauterine environment: a genome-wide association study. \| 2012 \| no main effect \| \| Paus \| Corpus callosum in adolescent offspring exposed prenatally to maternal cigarette smoking. \| 2008 \| no whole brain \| \| Pausova \| Genes, maternal smoking, and the offspring brain and body during adolescence: design of the Saguenay Youth Study. \| 2007 \| no significant interaction \| \| Pechtel \| Sensitive periods of amygdala development: The role of maltreatment in preadolescence. \| 2014 \| no whole brain \| \| Pederson \| Hippocampal volume and memory performace in a community-based sample of women with posttraumatic stress disorder secondary to child abuse \| 2004 \| no whole brain \| \| Perez \| Cingulo-insular structural alterations associated with psychogenic symptoms, childhood abuse and PTSD in functional neurological disorders. \| 2017 \| no whole brain \| \| Peters \| The roles of early-life adversity and rumination in neural response to emotional faces amongst anxious and depressed adults. \| 2019 \| functional activity \| \| Peterson \| Associations of Maternal Prenatal Drug Abuse With Measures of Newborn Brain Structure, Tissue Organization, and Metabolite Concentrations. \| 2020 \| structural MRI \| \| Peterson \| Effects of prenatal exposure to air pollutants (polycyclic aromatic hydrocarbons) on the development of brain white matter, cognition, and behavior in later childhood. \| 2015 \| structural MRI \| \| Philip \| Exposure to childhood trauma is associated with altered n-back activation and performance in healthy adults: implications for a commonly used working memory task. \| 2016 \| irrelevant domain \| \| Philip \| Early life stress is associated with greater default network deactivation during working memory in healthy controls: a preliminary report. \| 2013 \| no whole brain \| \| Picchioni \| Familial and environmental influences on brain volumes in twins with schizophrenia. \| 2017 \| no whole brain \| \| Piccolo \| Perceived stress is associated with smaller hippocampal volume in adolescence. \| 2018 \| no whole brain \| \| Piccolo \| Age-related differences in cortical thickness vary by socioeconomic status. \| 2016 \| structural MRI \| \| Poletti \| Effect of early stress on hippocampal gray matter is influenced by a functional polymorphism in EAAT2 in bipolar disorder. \| 2014 \| no whole brain \| \| Poletti \| Adverse childhood experiences influence the detrimental effect of bipolar disorder and schizophrenia on cortico-limbic grey matter volumes. \| 2016 \| structural MRI \| \| Popovic \| Traces of Trauma: A Multivariate Pattern Analysis of Childhood Trauma, Brain Structure, and Clinical Phenotypes. \| 2020 \| structural MRI \| \| Poppa \| Sexual trauma history is associated with reduced orbitofrontal network strength in substance-dependent women. \| 2019 \| no ACE \| \| Porto \| Morphometry of the pituitary gland and hypothalamus in long-term survivors of childhood trauma. \| 2011 \| no whole brain \| \| Powers \| Neural correlates and structural markers of emotion dysregulation in traumatized civilians. \| 2017 \| no ACE \| \| Pozzi \| Interaction between hypothalamic-pituitary-adrenal axis genetic variation and maternal behavior in the prediction of amygdala connectivity in children. \| 2019 \| no main effect \| \| Prasad \| Cognitive and neuroimaging findings in physically abused preschoolers. \| 2005 \| no MRI \| \| Puetz \| Altered neural response to rejection-related words in children exposed to maltreatment. \| 2016 \| functional activity \| \| Puetz \| Investigating patterns of neural response associated with childhood abuse v. childhood neglect. \| 2020 \| functional activity \| \| Puetz \| Neural response to social rejection in children with early separation experiences. \| 2014 \| irrelevant domain \| \| Pulido \| Family history of alcohol-use disorders and spatial working memory: Effects on adolescent alcohol expectancies. \| 2009 \| no whole brain \| \| Qiu \| Maternal anxiety and infants’ hippocampal development: timing matters. \| 2013 \| no whole brain \| \| Qiu \| Effects of antenatal maternal depressive symptoms and socio-economic status on neonatal brain development are modulated by genetic risk. \| 2017 \| structural MRI \| \| Quevedo \| The neurobiology of self-processing in abused depressed adolescents. \| 2017 \| functional activity \| \| Quevedo \| Brain activity and infant attachment history in young men during loss and reward processing. \| 2017 \| no significant interaction \| \| Qui \| COMT haplotypes modulate associations of antenatal maternal anxiety and neonatal cortical morphology. \| 2015 \| no significant interaction \| \| Qui \| Effects of Antenatal Maternal Depressive Symptoms and Socio-Economic Status on Neonatal Brain Development are Modulated by Genetic Risk. \| 2017 \| no whole brain \| \| Qui \| Prenatal maternal depression alters amygdala functional connectivity in 6-month-old infants. \| 2015 \| no whole brain \| \| Quidé \| Childhood trauma-related alterations in brain function during a Theory-of-Mind task in schizophrenia. \| 2017 \| irrelevant domain \| \| Quidé \| Effects of childhood trauma on working memory in affective and non-affective psychotic disorders. \| 2017 \| irrelevant domain \| \| Quidé \| Systemic inflammation and grey matter volume in schizophrenia and bipolar disorder: Moderation by childhood trauma severity. \| 2020 \| structural MRI \| \| Rabl \| Additive gene-environment effects on hippocampal structure in healthy humans. \| 2014 \| no ACE \| \| Raffington \| Blunted cortisol stress reactivity in low-income children relates to lower memory function. \| 2018 \| no whole brain \| \| Raine \| Reduced right hemisphere activation in severely abused violent offenders during a working memory task: An fMRI study. \| 2001 \| no whole brain \| \| Raizada \| Socioeconomic status predicts hemispheric specialisation of the left inferior frontal gyrus in young children. \| 2008 \| no whole brain \| \| Rajaprakash \| Cortical morphology in children with alcohol-related neurodevelopmental disorder. \| 2014 \| structural MRI \| \| Rando \| Prenatal cocaine exposure and gray matter volume in adolescent boys and girls: relationship to substance use initiation. \| 2013 \| structural MRI \| \| Rao \| Early parental care is important for hippocampal maturation: evidence from brain morphology in humans. \| 2010 \| no ACE \| \| Rao \| Altered resting cerebral blood flow in adolescents with in utero cocaine exposure revealed by perfusion functional MRI. \| 2007 \| no whole brain \| \| Rao \| Hippocampal changes associated with early-life adversity and vulnerability to depression. \| 2010 \| no whole brain \| \| Rätsep \| Brain structural and vascular anatomy is altered in offspring of pre-eclamptic pregnancies: A pilot study. \| 2016 \| no significant interaction \| \| Rattel \| Peritraumatic neural processing and intrusive memories: the role of lifetime adversity \| 2019 \| functional activity \| \| Rauh \| Brain anomalies in children exposed prenatally to a common organophosphate pesticide. \| 2012 \| structural MRI \| \| Real \| Brain structural correlates of obsessive-compulsive disorder with and without preceding stressful life events. \| 2016 \| no ACE \| \| Redlich \| Evidence of an IFN-gamma by early life stress interaction in the regulation of amygdala reactivity to emotional stimuli. \| 2015 \| no significant interaction \| \| Reed \| Interaction of childhood urbanicity and variation in dopamine genes alters adult prefrontal function as measured by functional magnetic resonance imaging (fMRI). \| 2018 \| irrelevant domain \| \| Regier \| Emotional, physical and sexual abuse are associated with a heightened limbic response to cocaine cues. \| 2017 \| irrelevant domain \| \| Richards \| Developmentally sensitive interaction effects of genes and the social environment on total and subcortical brain volumes. \| 2016 \| no main effect \| \| Richert \| Regional differences of the prefrontal cortex in pediatric PTSD: an MRI study. \| 2006 \| no whole brain \| \| Richmond \| Structural covariance networks in children and their associations with maternal behaviors. \| 2019 \| structural MRI \| \| Rifkin-Graboi \| Prenatal maternal depression associates with microstructure of right amygdala in neonates at birth. \| 2013 \| no significant interaction \| \| Riggins \| Memory ability and hippocampal volume in adolescents with prenatal drug exposure. \| 2012 \| no significant interaction \| \| Riikonen \| Deep serotonergic and dopaminergic structures in fetal alcoholic syndrome: A study with nor-ß-CIT-single-photon emission computed tomography and magnetic resonance imaging volumetry. \| 2005 \| structural MRI \| \| Rivkin \| Volumetric MRI study of brain in children with intrauterine exposure to cocaine, alcohol, tobacco, and marijuana. \| 2008 \| structural MRI \| \| Robey \| Relations among prospective memory, cognitive abilities, and brain structure in adolescents who vary in prenatal drug exposure. \| 2014 \| no whole brain \| \| Rodman \| Neurobiological markers of resilience to depression following childhood maltreatment: The role of neural circuits supporting the cognitive control of emotion. \| 2019 \| functional activity \| \| Roebuck \| Interhemispheric transfer in children with heavy prenatal alcohol exposure. \| 2002 \| no whole brain \| \| Roee \| Subtle findings on fetal brain imaging in CMV infected pregnancies: What is the clinical significance? A retrospective analysis with outcome correlation. \| 2020 \| irrelevant measure \| \| Romens \| Adolescent girls’ neural response to reward mediates the relation between childhood financial disadvantage and depression. \| 2015 \| functional activity \| \| Romeo \| Socioeconomic status and reading disability: neuroanatomy and plasticity in response to intervention. \| 2018 \| structural MRI \| \| Romund \| Maternal parenting behavior and emotion processing in adolescents-An fMRI study. \| 2016 \| no ACE \| \| Roos \| Effects of prenatal substance exposure on neurocognitive correlates of inhibitory control success and failure. \| 2017 \| functional activity \| \| Roos \| Structural brain changes in prenatal methamphetamine-exposed children. \| 2014 \| structural MRI \| \| Roos \| Structural brain network development in children following prenatal methamphetamine exposure. \| 2020 \| structural MRI \| \| Rosen \| Socioeconomic disparities in academic achievement: A multi-modal investigation of neural mechanisms in children and adolescents. \| 2018 \| structural MRI \| \| Rosso \| Neighborhood socioeconomic status and cognitive function in late life. \| 2016 \| no MRI \| \| Roussotte \| Abnormal brain activation during working memory in children with prenatal exposure to drugs of abuse: the effects of methamphetamine, alcohol, and polydrug exposure \| 2011 \| functional activity \| \| Roussotte \| Adolescents with prenatal cocaine exposure show subtle alterations in striatal surface morphology and frontal cortical volumes. \| 2012 \| no significant interaction \| \| Roussotte \| Regional brain volume reductions relate to facial dysmorphology and neurocognitive function in fetal alcohol spectrum disorders. \| 2012 \| structural MRI \| \| Sachsse \| Do childhood psychological trauma result in neurobiological changes in the adult brain? \| 2013 \| other language \| \| Sagiv \| Prenatal organophosphate pesticide exposure and traits related to autism spectrum disorders in a population living in proximity to agriculture. \| 2018 \| no MRI \| \| Samadi \| Children born to women treated for hypothyroidism during pregnancy show abnormal corpus callosum development. \| 2015 \| no whole brain \| \| Samplin \| Sex differences in resilience to childhood maltreatment: effects of trauma history on hippocampal volume, general cognition and subclinical psychosis in healthy adults. \| 2013 \| no whole brain \| \| Sandman \| Cortical thinning and neuropsychiatric outcomes in children exposed to prenatal adversity: A role for placental CRH? \| 2018 \| structural MRI \| \| Sandman \| Fetal exposure to maternal depressive symptoms is associated with cortical thickness in late childhood. \| 2015 \| structural MRI \| \| Santhanam \| Effects of prenatal alcohol exposure on brain activation during an arithmetic task: an fMRI study. \| 2009 \| no whole brain \| \| Savulich \| Effects of naltrexone are influenced by childhood adversity during negative emotional processing in addiction recovery. \| 2017 \| no main effect \| \| Saxbe \| Longitudinal associations between family aggression, externalizing behavior, and the structure and function of the amygdala. \| 2018 \| no whole brain \| \| Scharfenort \| Adversity-induced relapse of fear: neural mechanisms and implications for relapse prevention from a study on experimentally induced return-of-fear following fear conditioning and extinction. \| 2016 \| irrelevant domain \| \| Schechter \| Methylation of NR3C1 is related to maternal PTSD, parenting stress and maternal medial prefrontal cortical activity in response to child separation among mothers with histories of violence exposure. \| 2015 \| irrelevant domain \| \| Schechter \| The association of serotonin receptor 3A methylation with maternal violence exposure, neural activity, and child aggression. \| 2017 \| irrelevant domain \| \| Schwartz \| Behavioral inhibition in childhood predicts smaller hippocampal volume in adolescent offspring of parents with panic disorder. \| 2015 \| no whole brain \| \| Schweitzer \| Prenatal drug exposure to illicit drugs alters working memory-related brain activity and underlying network properties in adolescence. \| 2015 \| irrelevant domain \| \| Schweizer \| Enhanced emotion regulation capacity and its neural substrates in those exposed to moderate childhood adversity. \| 2015 \| no significant interaction \| \| Seghete \| General and emotion-specific alterations to cognitive control in women with a history of childhood abuse. \| 2017 \| functional activity \| \| Seghete \| Association between initial age of exposure to childhood abuse and cognitive control: preliminary evidence. \| 2018 \| no ACE \| \| Segreti \| Cortical thickness and volume reductions in young adults with current suicidal ideation. \| 2019 \| structural MRI \| \| Sellnow \| Isotypes of functional brain engagement during emotion processing differentiate heterogeneity in internalizing symptoms and interpersonal violence histories among adolescent girls \| 2020 \| functional activity \| \| Sethi \| Primary and secondary variants of psychopathy in a volunteer sample are associated with different neurocognitive mechanisms. \| 2018 \| functional activity \| \| Sethna \| Mother-infant interactions and regional brain volumes in infancy: an MRI study. \| 2017 \| irrelevant domain \| \| Shaked \| Dorsolateral prefrontal cortex volume as a mediator between socioeconomic status and executive function. \| 2018 \| structural MRI \| \| Shaked \| Sociodemographic disparities in corticolimbic structures. \| 2019 \| structural MRI \| \| Shang \| Decreased BOLD fluctuations in lateral temporal cortices of premature born adults. \| 2018 \| irrelevant measure \| \| Shanmugan \| Impact of tryptophan depletion on executive system function during menopause is moderated by childhood adversity. \| 2017 \| irrelevant domain \| \| Sharma \| Differentiating the Effects of Familial Risk for Alcohol Dependence and Prenatal Exposure to Alcohol on Offspring Brain Morphology. \| 2017 \| structural MRI \| \| Sharp \| Major depression in mothers predicts reduced ventral striatum activation in adolescent female offspring with and without depression. \| 2014 \| no significant interaction \| \| Sheffield \| Reduced gray matter volume in psychotic disorder patients with a history of childhood sexual abuse. \| 2013 \| structural MRI \| \| Sheinkopf \| Functional MRI and response inhibition in children exposed to cocaine in utero. Preliminary findings. \| 2009 \| functional activity \| \| Sheridan \| Dimensions of childhood adversity have distinct associations with neural systems underlying executive functioning. \| 2017 \| irrelevant domain \| \| Sheridan \| The impact of social disparity on prefrontal function in childhood. \| 2012 \| no whole brain \| \| Sheridan \| What are the links between maternal social status, hippocampal function, and HPA axis function in children? \| 2013 \| no whole brain \| \| Sheridan \| Variation in neural development as a result of exposure to institutionalization early in childhood \| 2012 \| structural MRI \| \| Sheu \| Harsh corporal punishment is associated with increased T2 relaxation time in dopamine-rich regions. \| 2010 \| irrelevant domain \| \| Shimada \| Reduced visual cortex grey matter volume in children and adolescents with reactive attachment disorder. \| 2015 \| structural MRI \| \| Shvil \| Sex differences in extinction recall in posttraumatic stress disorder: a pilot fMRI study. \| 2014 \| no ACE \| \| Sierk \| Allocentric spatial memory performance predicts intrusive memory severity in posttraumatic stress disorder. \| 2019 \| structural MRI \| \| Silvers \| Previous institutionalization Is followed by broader amygdala-Hippocampal-PFC network connectivity during aversive learning in human development. \| 2016 \| irrelevant domain \| \| Simmons \| Study protocol: families and childhood transitions study (FACTS) - a longitudinal investigation of the role of the family environment in brain development and risk for mental health disorders in community based children. \| 2017 \| no experimental article \| \| Simpson \| Toward identifying reproducible brain signatures of obsessive-compulsive profiles: Rationale and methods for a new global initiative. \| 2020 \| no experimental article \| \| Singh \| Reward processing in healthy offspring of parents with bipolar disorder. \| 2014 \| functional activity \| \| Sirnes \| Functional MRI in prenatally opioid-exposed children during a working memory-selective attention task. \| 2018 \| functional activity \| \| Sirnes \| Brain morphology in school-aged children with prenatal opioid exposure: A structural MRI study. \| 2017 \| no significant interaction \| \| Sjoerds \| Family history of alcohol dependence and gray matter abnormalities in non-alcoholic adults. \| 2013 \| structural MRI \| \| Skokauskas \| The role of sexual abuse on functional neuroimaging markers associated with major depressive disorder. \| 2015 \| functional activity \| \| Smith \| Effects of prenatal marijuana on response inhibition: an fMRI study of young adults. \| 2004 \| functional activity \| \| Smith \| Effects of prenatal marijuana on visuospatial working memory: an fMRI study in young adults. \| 2006 \| functional activity \| \| Smith \| The effects of prenatal and current marijuana exposure on response inhibition: a functional magnetic resonance imaging study. \| 2004 \| no experimental article \| \| Smith \| Brain proton magnetic resonance spectroscopy and imaging in children exposed to cocaine in utero. \| 2001 \| no significant interaction \| \| Smith \| Brain proton magnetic resonance spectroscopy in children exposed to methamphetamine in utero. \| 2001 \| no significant interaction \| \| Song \| Cortical volumetric correlates of childhood trauma, anxiety, and impulsivity in bipolar disorder. \| 2020 \| structural MRI \| \| Sowell \| Functional magnetic resonance imaging of verbal learning in children with heavy prenatal alcohol exposure. \| 2007 \| irrelevant domain \| \| Sowell \| Abnormal cortical thickness and brain-behavior correlation patterns in individuals with heavy prenatal alcohol exposure. \| 2008 \| no whole brain \| \| Sowell \| Mapping cortical gray matter asymmetry patterns in adolescents with heavy prenatal alcohol exposure. \| 2002 \| no whole brain \| \| Sowell \| Mapping callosal morphology and cognitive correlates - Effects of heavy prenatal alcohol exposure \| 2001 \| structural MRI \| \| Sowell \| Regional brain shape abnormalities persist into adolescence after heavy prenatal alcohol exposure. \| 2002 \| structural MRI \| \| Sowell \| Voxel-based morphometric analyses of the brain in children and adolescents prenatally exposed to alcohol. \| 2001 \| structural MRI \| \| Spadoni \| BOLD response during spatial working memory in youth with heavy prenatal alcohol exposure. \| 2009 \| functional activity \| \| Spann \| Prenatal socioeconomic status and social support are associated with neonatal brain morphology, toddler language and psychiatric symptoms. \| 2020 \| structural MRI \| \| Spielberg \| Adolescent development of inhibition as a function of SES and gender: Converging evidence from behavior and fMRI. \| 2015 \| no whole brain \| \| Spies \| Effects of HIV and childhood trauma on brain morphometry and neurocognitive function. \| 2016 \| structural MRI \| \| Sripada \| Trajectories of brain development in school-age children born preterm with very low birth weight. \| 2018 \| structural MRI \| \| Staff \| Childhood socioeconomic status and adult brain size: childhood socioeconomic status influences adult hippocampal size. \| 2012 \| structural MRI \| \| Stein \| Hippocampal volume in women victimized by childhood sexual abuse. \| 1997 \| no whole brain \| \| Steines \| Conflicting group memberships modulate neural activation in an emotional production-perception network. \| 2020 \| functional activity \| \| Steuwe \| Effect of direct eye contact in PTSD related to interpersonal trauma: an fMRI study of activation of an innate alarm system. \| 2014 \| irrelevant domain \| \| Stevens \| Childhood maltreatment predicts reduced inhibition-related activity in the rostral anterior cingulate in PTSD, but not trauma-exposed controls. \| 2016 \| no main effect \| \| Stevens \| Episodic memory after trauma exposure: Medial temporal lobe function is positively related to re-experiencing and inversely related to negative affect symptoms. \| 2018 \| no whole brain \| \| Stewart \| Prenatal PCB exposure, the corpus callosum, and response inhibition. \| 2003 \| no whole brain \| \| Strauss \| Touch aversion in patients with interpersonal traumatization. \| 2019 \| functional activity \| \| Sun \| Structural covariance network centrality in maltreated youth with posttraumatic stress disorder. \| 2018 \| irrelevant domain \| \| Sun \| Brain structural covariance network centrality in maltreated youth with PTSD and in maltreated youth resilient to PTSD. \| 2019 \| structural MRI \| \| Suttie \| Combined face–brain morphology and associated neurocognitive correlates in fetal alcohol spectrum disorders. \| 2018 \| structural MRI \| \| Suzuki \| Early life stress and trauma and enhanced limbic activation to emotionally valenced faces in depressed and healthy children. \| 2014 \| functional activity \| \| Swartz \| Developmental change in amygdala reactivity during adolescence: effects of family history of depression and stressful life events. \| 2015 \| no whole brain \| \| Swartz \| Post-secondary maternal education buffers against neural risk for psychological vulnerability to future life stress. \| 2018 \| no whole brain \| \| Takiguchi \| Ventral striatum dysfunction in children and adolescents with reactive attachment disorder: functional MRI study. \| 2015 \| functional activity \| \| Tannous \| Stress, inflammation and hippocampal subfields in depression: A 7 Tesla MRI Study. \| 2020 \| structural MRI \| \| Tanyildiz \| How does B12 deficiency of mothers affect their infants? \| 2017 \| no MRI \| \| Taylor \| Neural responses to emotional stimuli are associated with childhood family stress. \| 2006 \| functional activity \| \| Teicher \| Does sleep disruption mediate the effects of childhood maltreatment on brain structure? \| 2017 \| no main effect \| \| Teicher \| Childhood maltreatment is associated with reduced volume in the hippocampal subfields CA3, dentate gyrus, and subiculum. \| 2012 \| no whole brain \| \| Teicher \| Childhood maltreatment: altered network centrality of cingulate, precuneus, temporal pole and insula. \| 2014 \| no whole brain \| \| Teicher \| Differential effects of childhood neglect and abuse during sensitive exposure periods on male and female hippocampus. \| 2018 \| no whole brain \| \| Telzer \| Early experience shapes amygdala sensitivity to race: An international adoption design. \| 2013 \| no significant interaction \| \| Tervo-Clemmens \| Adolescent cannabis use and brain systems supporting adult working memory encoding, maintenance, and retrieval. \| 2018 \| no ACE \| \| Thomaes \| Treatment effects on insular and anterior cingulate cortex activation during classic and emotional Stroop interference in child abuse-related complex post-traumatic stress disorder. \| 2012 \| functional activity \| \| Thomaes \| Increased anterior cingulate cortex and hippocampus activation in Complex PTSD during encoding of negative words. \| 2013 \| no whole brain \| \| Thomaes \| Reduced anterior cingulate and orbitofrontal volumes in child abuse-related complex PTSD. \| 2010 \| structural MRI \| \| Thomas \| Pituitary volumes in pediatric maltreatment-related posttraumatic stress disorder. \| 2004 \| no whole brain \| \| Thompson \| Basal ganglia and thalamic tract connectivity in very preterm and full-term children; associations with 7-year neurodevelopment. \| 2020 \| structural MRI \| \| Thompson \| Characterisation of brain volume and microstructure at term-equivalent age in infants born across the gestational age spectrum. \| 2019 \| structural MRI \| \| Thompson \| Early life predictors of brain development at term-equivalent age in infants born across the gestational age spectrum. \| 2019 \| structural MRI \| \| Tokuda \| Identification of depression subtypes and relevant brain regions using a data-driven approach. \| 2018 \| no main effect \| \| Tomlinson \| Neighborhood poverty predicts altered neural and behavioral response inhibition. \| 2020 \| functional activity \| \| Tomoda \| Childhood sexual abuse is associated with reduced gray matter volume in visual cortex of young women. \| 2009 \| structural MRI \| \| Tomoda \| Exposure to parental verbal abuse is associated with increased gray matter volume in superior temporal gyrus. \| 2011 \| structural MRI \| \| Tomoda \| Reduced prefrontal cortical gray matter volume in young adults exposed to harsh corporal punishment. \| 2009 \| structural MRI \| \| Tomoda \| Reduced visual cortex gray matter volume and thickness in young adults who witnessed domestic violence during childhood. \| 2012 \| structural MRI \| \| Tooley \| Associations between neighborhood SES and functional brain network development. \| 2020 \| no main effect \| \| Toro \| Prenatal exposure to maternal cigarette smoking and the adolescent cerebral cortex. \| 2008 \| no whole brain \| \| Tottenham \| Prolonged institutional rearing is associated with atypically large amygdala volume and difficulties in emotion regulation \| 2010 \| no significant interaction \| \| Tottenham \| Elevated amygdala response to faces following early deprivation \| 2011 \| no whole brain \| \| Tozzi \| Single-nucleotide polymorphism of the FKBP5 gene and childhood maltreatment as predictors of structural changes in brain areas involved in emotional processing in depression. \| 2016 \| no significant interaction \| \| Tozzi \| Epigenetic changes of FKBP5 as a link connecting genetic and environmental risk factors with structural and functional brain changes in major depression. \| 2018 \| no whole brain \| \| Tozzi \| Interactive impact of childhood maltreatment, depression, and age on cortical brain structure: mega-analytic findings from a large multi-site cohort. \| 2019 \| structural MRI \| \| Treadway \| Early adverse events, HPA activity and rostral anterior cingulate volume in MDD \| 2009 \| no significant interaction \| \| Treit \| Longitudinal MRI reveals impaired cortical thinning in children and adolescents prenatally exposed to alcohol. \| 2014 \| structural MRI \| \| Treit \| Relationships between head circumference, brain volume and cognition in children with prenatal alcohol exposure. \| 2016 \| structural MRI \| \| Treit \| Sexual dimorphism of volume reduction but not cognitive deficit in fetal alcohol spectrum disorders: A combined diffusion tensor imaging, cortical thickness and brain volume study. \| 2017 \| structural MRI \| \| Tupler \| Segmented hippocampal volume in children and adolescents with posttraumatic stress disorder. \| 2006 \| no whole brain \| \| Turesky \| Relating anthropometric indicators to brain structure in 2-month-old Bangladeshi infants growing up in poverty: A pilot study. \| 2020 \| structural MRI \| \| Tyborowska \| Early-life and pubertal stress differentially modulate grey matter development in human adolescents. \| 2018 \| structural MRI \| \| Uban \| The Relationship Between Socioeconomic Status and Brain Volume in Children and Adolescents With Prenatal Alcohol Exposure \| 2020 \| structural MRI \| \| Urbain \| Converging function, structure, and behavioural features of emotion regulation in very preterm children. \| 2019 \| functional activity \| \| Vai \| Corticolimbic connectivity mediates the relationship between adverse childhood experiences and symptom severity in borderline personality disorder. \| 2018 \| FT not found \| \| Vai \| Mild adverse childhood experiences increase neural efficacy during affective theory of mind. \| 2018 \| irrelevant domain \| \| Vai \| Cortico-limbic functional connectivity mediates the effect of early life stress on suicidality in bipolar depressed 5-HTTLPR*s carriers. \| 2020 \| no significant interaction \| \| Van Dam \| Childhood maltreatment, altered limbic neurobiology, and substance use relapse severity via trauma-specific reductions in limbic gray matter volume. \| 2014 \| structural MRI \| \| van den Berg \| Pass it on? The neural responses to rejection in the context of a family study on maltreatment. \| 2018 \| no whole brain \| \| van den Bulk \| Amygdala habituation to emotional faces in adolescents with internalizing disorders, adolescents with childhood sexual abuse related PTSD and healthy adolescents. \| 2016 \| no whole brain \| \| van den Dries \| Prenatal exposure to organophosphate pesticides and brain morphology and white matter microstructure in preadolescents \| 2020 \| structural MRI \| \| van der Knaap \| Maternal depressive symptoms during pregnancy are associated with amygdala hyperresponsivity in children. \| 2018 \| no whole brain \| \| van der Meer \| Effects of dopaminergic genes, prenatal adversities, and their interaction on attention-deficit/hyperactivity disorder and neural correlates of response inhibition. \| 2017 \| functional activity \| \| van der Meulen \| Genetic and environmental influences on structure of the social brain in childhood. \| 2020 \| structural MRI \| \| van Harmelen \| Childhood emotional maltreatment severity is associated with dorsal medial prefrontal cortex responsivity to social exclusion in young adults. \| 2014 \| irrelevant domain \| \| van Harmelen \| Hypoactive medial prefrontal cortex functioning in adults reporting childhood emotional maltreatment. \| 2013 \| irrelevant domain \| \| van Harmelen \| Enhanced amygdala reactivity to emotional faces in adults reporting childhood emotional maltreatment. \| 2013 \| no significant interaction \| \| van Harmelen \| Reduced medial prefrontal cortex volume in adults reporting childhood emotional maltreatment. \| 2010 \| structural MRI \| \| van Hoof \| Emotional face processing in adolescents with childhood sexual abuse-related posttraumatic stress disorder, internalizing disorders and healthy controls. \| 2017 \| no significant interaction \| \| van Rooij \| Childhood trauma and COMT genotype interact to increase hippocampal activation in resilient individuals. \| 2016 \| no main effect \| \| van Schie \| The neural correlates of childhood maltreatment and the ability to understand mental states of others. \| 2017 \| irrelevant domain \| \| van Velzen \| Effect of childhood maltreatment and brain-derived neurotrophic factor on brain morphology. \| 2016 \| no whole brain \| \| Vandewouw \| Mapping the neuroanatomical impact of very preterm birth across childhood. \| 2020 \| structural MRI \| \| Veer \| Evidence for smaller right amygdala volumes in posttraumatic stress disorder following childhood trauma. \| 2015 \| no whole brain \| \| Vermetten \| Hippocampal and amygdalar volumes in dissociative identity disorder. \| 2006 \| no whole brain \| \| Vythilingam \| Childhood trauma associated with smaller hippocampal volume in women with major depression. \| 2002 \| no whole brain \| \| Waldstein \| Differential associations of socioeconomic status with global brain volumes and white matter lesions in african american and white adults: the HANDLS SCAN study. \| 2017 \| no ACE \| \| Walhovd \| Child neuroanatomical, neurocognitive, and visual acuity outcomes with maternal opioid and polysubstance detoxification. \| 2015 \| no significant interaction \| \| Walhovd \| Volumetric cerebral characteristics of children exposed to opiates and other substances in utero. \| 2007 \| structural MRI \| \| Waller \| Accelerated alcohol use across adolescence predicts early adult symptoms of alcohol use disorder via reward-related neural function. \| 2019 \| no ACE \| \| Walsh \| 5-HTTLPR-environment interplay and its effects on neural reactivity in adolescents. \| 2012 \| no main effect \| \| Walsh \| General and specific effects of early-life psychosocial adversities on adolescent grey matter volume. \| 2014 \| structural MRI \| \| Walton \| Longitudinal epigenetic predictors of amygdala:hippocampus volume ratio. \| 2017 \| no whole brain \| \| Wang \| Loss of sustained activity in the ventromedial prefrontal cortex in response to repeated stress in individuals with early-life emotional abuse: implications for depression vulnerability. \| 2013 \| no main effect \| \| Wang \| Altered resting functional network topology assessed using graph theory in youth with attention-deficit/hyperactivity disorder. \| 2020 \| no ACE \| \| Wang \| FKBP5 Moderates the Association between Antenatal Maternal Depressive Symptoms and Neonatal Brain Morphology. \| 2018 \| no significant interaction \| \| Wang \| Pathway to neural resilience: Self-esteem buffers against deleterious effects of poverty on the hippocampus. \| 2016 \| structural MRI \| \| Ware \| An fMRI study of behavioral response inhibition in adolescents with and without histories of heavy prenatal alcohol exposure. \| 2015 \| functional activity \| \| Warren \| Anxiety and stress alter decision-making dynamics and causal amygdala-dorsolateral prefrontal cortex circuits during emotion regulation in children \| 2020 \| no ACE \| \| Warton \| Prenatal methamphetamine exposure is associated with reduced subcortical volumes in neonates. \| 2018 \| no significant interaction \| \| Weems \| Developmental variation in amygdala volumes among children with posttraumatic stress. \| 2013 \| no whole brain \| \| Weismann \| Reduced hippocampal and amygdala volume as a mechanism underlying stress sensitization to depression following childhood trauma \| 2020 \| structural MRI \| \| Weissman \| Tuning of brain-autonomic coupling by prior threat exposure: Implications for internalizing problems in Mexican-origin adolescents. \| 2019 \| functional activity \| \| Weissman \| Income change alters default mode network connectivity for adolescents in poverty. \| 2018 \| no significant interaction \| \| Wen \| Influences of prenatal and postnatal maternal depression on amygdala volume and microstructure in young children. \| 2017 \| structural MRI \| \| Weniger \| Egocentric virtual maze learning in adult survivors of childhood abuse with dissociative disorders: evidence from functional magnetic resonance imaging. \| 2013 \| irrelevant domain \| \| Weniger \| Amygdala and hippocampal volumes and cognition in adult survivors of childhood abuse with dissociative disorders. \| 2008 \| no whole brain \| \| Weniger \| Reduced amygdala and hippocampus size in trauma-exposed women with borderline personality disorder and without posttraumatic stress disorder. \| 2009 \| no whole brain \| \| White \| Functional MRI approach to developmental methylmercury and polychlorinated biphenyl neurotoxicity. \| 2011 \| irrelevant domain \| \| White \| FKBP5 and emotional neglect interact to predict individual differences in amygdala reactivity. \| 2012 \| no whole brain \| \| Whittle \| Maternal responses to adolescent positive affect are associated with adolescents’ reward neuroanatomy. \| 2009 \| no ACE \| \| Whittle \| Childhood maltreatment, psychopathology, and the development of hippocampal subregions during adolescence. \| 2017 \| no whole brain \| \| Whittle \| Childhood maltreatment and psychopathology affect brain development during adolescence. \| 2013 \| structural MRI \| \| Whittle \| Role of positive parenting in the association between neighborhood social disadvantage and brain development across adolescence. \| 2017 \| structural MRI \| \| Willford \| An examination of the association between prenatal cocaine exposure and brain activation measures of arousal and attention in young adults: An fMRI study using the Attention Network Task. \| 2018 \| functional activity \| \| Willford \| Caudate asymmetry: a neurobiological marker of moderate prenatal alcohol exposure in young adults. \| 2010 \| no significant interaction \| \| Williams \| “Negativity bias” in risk for depression and anxiety: brain-body fear circuitry correlates, 5-HTT-LPR and early life stress. \| 2009 \| no whole brain \| \| Willoughby \| Effects of maternal hypothyroidism on offspring hippocampus and memory. \| 2014 \| no significant interaction \| \| Willoughby \| Effects of prenatal alcohol exposure on hippocampal volume, verbal learning, and verbal and spatial recall in late childhood. \| 2008 \| no whole brain \| \| Wolf \| Prefrontal-amygdala dysregulation to threat in pediatric posttraumatic stress disorder. \| 2016 \| functional activity \| \| Woods \| Prenatal alcohol exposure affects brain function during place learning in a virtual environment differently in boys and girls. \| 2018 \| functional activity \| \| Woods \| Altered parietal activation during non-symbolic number comparison in children with prenatal alcohol exposure. \| 2018 \| no whole brain \| \| Woods \| Parietal dysfunction during number processing in children with fetal alcohol spectrum disorders. \| 2015 \| no whole brain \| \| Woodward \| Early adversity and combat exposure interact to influence anterior cingulate cortex volume in combat veterans. \| 2013 \| no whole brain \| \| Wozniak \| Inter-hemispheric functional connectivity disruption in children with prenatal alcohol exposure. \| 2011 \| no whole brain \| \| Wright \| Mothers who were neglected in childhood show differences in neural response to their infant’s cry. \| 2017 \| irrelevant domain \| \| Wymbs \| Social supports moderate the effects of child adversity on neural correlates of threat processing. \| 2020 \| functional activity \| \| Xerxa \| Association of Poor Family Functioning From Pregnancy Onward With Preadolescent Behavior and Subcortical Brain Development \| 2020 \| structural MRI \| \| Yamamoto \| Increased amygdala reactivity following early life stress: a potential resilience enhancer role. \| 2017 \| no whole brain \| \| Yang \| Interaction between early life stress and alcohol dependence on neural stress reactivity. \| 2015 \| irrelevant domain \| \| Yang \| Effects of parental emotional warmth on the relationship between regional gray matter volume and depression-related personality traits. \| 2017 \| no ACE \| \| Yang \| Regional gray matter volume mediates the relationship between family socioeconomic status and depression-related trait in a young healthy sample. \| 2016 \| no ACE \| \| Yang \| Abnormal cortical thickness alterations in fetal alcohol spectrum disorders and their relationships with facial dysmorphology. \| 2012 \| structural MRI \| \| Yang \| Callosal Thickness Reductions Relate to Facial Dysmorphology in Fetal Alcohol Spectrum Disorders. \| 2012 \| structural MRI \| \| Yang \| Childhood maltreatment is associated with gray matter volume abnormalities in patients with first-episode depression. \| 2017 \| structural MRI \| \| Yaple \| Functional and structural brain correlates of socioeconomic status \| 2020 \| no experimental article \| \| Yip \| Prenatal cocaine exposure and adolescent neural responses to appetitive and stressful stimuli. \| 2014 \| irrelevant domain \| \| Yip \| Prenatal cocaine exposure, illicit-substance use and stress and craving processes during adolescence. \| 2016 \| irrelevant domain \| \| Yoon \| Salivary biomarkers of neural hypervigilance in trauma-exposed women. \| 2016 \| no ACE \| \| Younger \| Brain lateralization of phonological awareness varies by maternal education. \| 2019 \| functional activity \| \| Yuan \| Do maternal opioids reduce neonatal regional brain volumes? A pilot study. \| 2014 \| no significant interaction \| \| Yuan \| Smaller left hippocampal subfield CA1 volume is associated with reported childhood physical and/or sexual abuse in major depression: A pilot study. \| 2020 \| structural MRI \| \| Zanetti \| MRI study of corpus callosum in patients with borderline personality disorder: a pilot study. \| 2007 \| no whole brain \| \| Zhong \| Childhood maltreatment experience influences neural response to psychosocial stress in adults: an fMRI study. \| 2020 \| functional activity \| \| Zhou \| Preserved cortical asymmetry despite thinner cortex in children and adolescents with prenatal alcohol exposure and associated conditions. \| 2018 \| structural MRI \| \| Zhu \| Association of prepubertal and postpubertal exposure to childhood maltreatment with adult amygdala function. \| 2019 \| functional activity \| \| Zou \| Exposure to maternal depressive symptoms in fetal life or childhood and offspring brain development: A population-based imaging study \| 2019 \| structural MRI \| |
| --- | --- | --- | --- | --- | --- | --- | --- | --- | --- | --- | --- | --- | --- | --- | --- | --- | --- | --- | --- | --- | --- | --- | --- | --- | --- | --- | --- | --- | --- | --- | --- | --- | --- | --- | --- | --- | --- | --- | --- | --- | --- | --- | --- | --- | --- | --- | --- | --- | --- | --- | --- | --- | --- | --- | --- | --- | --- | --- | --- | --- | --- | --- | --- | --- | --- | --- | --- | --- | --- | --- | --- | --- | --- | --- | --- | --- | --- | --- | --- | --- | --- | --- | --- | --- | --- | --- | --- | --- | --- | --- | --- | --- | --- | --- | --- | --- | --- | --- | --- | --- | --- | --- | --- | --- | --- | --- | --- | --- | --- | --- | --- | --- | --- | --- | --- | --- | --- | --- | --- | --- | --- | --- | --- | --- | --- | --- | --- | --- | --- | --- | --- | --- | --- | --- | --- | --- | --- | --- | --- | --- | --- | --- | --- | --- | --- | --- | --- | --- | --- | --- | --- | --- | --- | --- | --- | --- | --- | --- | --- | --- | --- | --- | --- | --- | --- | --- | --- | --- | --- | --- | --- | --- | --- | --- | --- | --- | --- | --- | --- | --- | --- | --- | --- | --- | --- | --- | --- | --- | --- | --- | --- | --- | --- | --- | --- | --- | --- | --- | --- | --- | --- | --- | --- | --- | --- | --- | --- | --- | --- | --- | --- | --- | --- | --- | --- | --- | --- | --- | --- | --- | --- | --- | --- | --- | --- | --- | --- | --- | --- | --- | --- | --- | --- | --- | --- | --- | --- | --- | --- | --- | --- | --- | --- | --- | --- | --- | --- | --- | --- | --- | --- | --- | --- | --- | --- | --- | --- | --- | --- | --- | --- | --- | --- | --- | --- | --- | --- | --- | --- | --- | --- | --- | --- | --- | --- | --- | --- | --- | --- | --- | --- | --- | --- | --- | --- | --- | --- | --- | --- | --- | --- | --- | --- | --- | --- | --- | --- | --- | --- | --- | --- | --- | --- | --- | --- | --- | --- | --- | --- | --- | --- | --- | --- | --- | --- | --- | --- | --- | --- | --- | --- | --- | --- | --- | --- | --- | --- | --- | --- | --- | --- | --- | --- | --- | --- | --- | --- | --- | --- | --- | --- | --- | --- | --- | --- | --- | --- | --- | --- | --- | --- | --- | --- | --- | --- | --- | --- | --- | --- | --- | --- | --- | --- | --- | --- | --- | --- | --- | --- | --- | --- | --- | --- | --- | --- | --- | --- | --- | --- | --- | --- | --- | --- | --- | --- | --- | --- | --- | --- | --- | --- | --- | --- | --- | --- | --- | --- | --- | --- | --- | --- | --- | --- | --- | --- | --- | --- | --- | --- | --- | --- | --- | --- | --- | --- | --- | --- | --- | --- | --- | --- | --- | --- | --- | --- | --- | --- | --- | --- | --- | --- | --- | --- | --- | --- | --- | --- | --- | --- | --- | --- | --- | --- | --- | --- | --- | --- | --- | --- | --- | --- | --- | --- | --- | --- | --- | --- | --- | --- | --- | --- | --- | --- | --- | --- | --- | --- | --- | --- | --- | --- | --- | --- | --- | --- | --- | --- | --- | --- | --- | --- | --- | --- | --- | --- | --- | --- | --- | --- | --- | --- | --- | --- | --- | --- | --- | --- | --- | --- | --- | --- | --- | --- | --- | --- | --- | --- | --- | --- | --- | --- | --- | --- | --- | --- | --- | --- | --- | --- | --- | --- | --- | --- | --- | --- | --- | --- | --- | --- | --- | --- | --- | --- | --- | --- | --- | --- | --- | --- | --- | --- | --- | --- | --- | --- | --- | --- | --- | --- | --- | --- | --- | --- | --- | --- | --- | --- | --- | --- | --- | --- | --- | --- | --- | --- | --- | --- | --- | --- | --- | --- | --- | --- | --- | --- | --- | --- | --- | --- | --- | --- | --- | --- | --- | --- | --- | --- | --- | --- | --- | --- | --- | --- | --- | --- | --- | --- | --- | --- | --- | --- | --- | --- | --- | --- | --- | --- | --- | --- | --- | --- | --- | --- | --- | --- | --- | --- | --- | --- | --- | --- | --- | --- | --- | --- | --- | --- | --- | --- | --- | --- | --- | --- | --- | --- | --- | --- | --- | --- | --- | --- | --- | --- | --- | --- | --- | --- | --- | --- | --- | --- | --- | --- | --- | --- | --- | --- | --- | --- | --- | --- | --- | --- | --- | --- | --- | --- | --- | --- | --- | --- | --- | --- | --- | --- | --- | --- | --- | --- | --- | --- | --- | --- | --- | --- | --- | --- | --- | --- | --- | --- | --- | --- | --- | --- | --- | --- | --- | --- | --- | --- | --- | --- | --- | --- | --- | --- | --- | --- | --- | --- | --- | --- | --- | --- | --- | --- | --- | --- | --- | --- | --- | --- | --- | --- | --- | --- | --- | --- | --- | --- | --- | --- | --- | --- | --- | --- | --- | --- | --- | --- | --- | --- | --- | --- | --- | --- | --- | --- | --- | --- | --- | --- | --- | --- | --- | --- | --- | --- | --- | --- | --- | --- | --- | --- | --- | --- | --- | --- | --- | --- | --- | --- | --- | --- | --- | --- | --- | --- | --- | --- | --- | --- | --- | --- | --- | --- | --- | --- | --- | --- | --- | --- | --- | --- | --- | --- | --- | --- | --- | --- | --- | --- | --- | --- | --- | --- | --- | --- | --- | --- | --- | --- | --- | --- | --- | --- | --- | --- | --- | --- | --- | --- | --- | --- | --- | --- | --- | --- | --- | --- | --- | --- | --- | --- | --- | --- | --- | --- | --- | --- | --- | --- | --- | --- | --- | --- | --- | --- | --- | --- | --- | --- | --- | --- | --- | --- | --- | --- | --- | --- | --- | --- | --- | --- | --- | --- | --- | --- | --- | --- | --- | --- | --- | --- | --- | --- | --- | --- | --- | --- | --- | --- | --- | --- | --- | --- | --- | --- | --- | --- | --- | --- | --- | --- | --- | --- | --- | --- | --- | --- | --- | --- | --- | --- | --- | --- | --- | --- | --- | --- | --- | --- | --- | --- | --- | --- | --- | --- | --- | --- | --- | --- | --- | --- | --- | --- | --- | --- | --- | --- | --- | --- | --- | --- | --- | --- | --- | --- | --- | --- | --- | --- | --- | --- | --- | --- | --- | --- | --- | --- | --- | --- | --- | --- | --- | --- | --- | --- | --- | --- | --- | --- | --- | --- | --- | --- | --- | --- | --- | --- | --- | --- | --- | --- | --- | --- | --- | --- | --- | --- | --- | --- | --- | --- | --- | --- | --- | --- | --- | --- | --- | --- | --- | --- | --- | --- | --- | --- | --- | --- | --- | --- | --- | --- | --- | --- | --- | --- | --- | --- | --- | --- | --- | --- | --- | --- | --- | --- | --- | --- | --- | --- | --- | --- | --- | --- | --- | --- | --- | --- | --- | --- | --- | --- | --- | --- | --- | --- | --- | --- | --- | --- | --- | --- | --- | --- | --- | --- | --- | --- | --- | --- | --- | --- | --- | --- | --- | --- | --- | --- | --- | --- | --- | --- | --- | --- | --- | --- | --- | --- | --- | --- | --- | --- | --- | --- | --- | --- | --- | --- | --- | --- | --- | --- | --- | --- | --- | --- | --- | --- | --- | --- | --- | --- | --- | --- | --- | --- | --- | --- | --- | --- | --- | --- | --- | --- | --- | --- | --- | --- | --- | --- | --- | --- | --- | --- | --- | --- | --- | --- | --- | --- | --- | --- | --- | --- | --- | --- | --- | --- | --- | --- | --- | --- | --- | --- | --- | --- | --- | --- | --- | --- | --- | --- | --- | --- | --- | --- | --- | --- | --- | --- | --- | --- | --- | --- | --- | --- | --- | --- | --- | --- | --- | --- | --- | --- | --- | --- | --- | --- | --- | --- | --- | --- | --- | --- | --- | --- | --- | --- | --- | --- | --- | --- | --- | --- | --- | --- | --- | --- | --- | --- | --- | --- | --- | --- | --- | --- | --- | --- | --- | --- | --- | --- | --- | --- | --- | --- | --- | --- | --- | --- | --- | --- | --- | --- | --- | --- | --- | --- | --- | --- | --- | --- | --- | --- | --- | --- | --- | --- | --- | --- | --- | --- | --- | --- | --- | --- | --- | --- | --- | --- | --- | --- | --- | --- | --- | --- | --- | --- | --- | --- | --- | --- | --- | --- | --- | --- | --- | --- | --- | --- | --- | --- | --- | --- | --- | --- | --- | --- | --- | --- | --- | --- | --- | --- | --- | --- | --- | --- | --- | --- | --- | --- | --- | --- | --- | --- | --- | --- | --- | --- | --- | --- | --- | --- | --- | --- | --- | --- | --- | --- | --- | --- | --- | --- | --- | --- | --- | --- | --- | --- | --- | --- | --- | --- | --- | --- | --- | --- | --- | --- | --- | --- | --- | --- | --- | --- | --- | --- | --- | --- | --- | --- | --- | --- | --- | --- | --- | --- | --- | --- | --- | --- | --- | --- | --- | --- | --- | --- | --- | --- | --- | --- | --- | --- | --- | --- | --- | --- | --- | --- | --- | --- | --- | --- | --- | --- | --- | --- | --- | --- | --- | --- | --- | --- | --- | --- | --- | --- | --- | --- | --- | --- | --- | --- | --- | --- | --- | --- | --- | --- | --- | --- | --- | --- | --- | --- | --- | --- | --- | --- | --- | --- | --- | --- | --- | --- | --- | --- | --- | --- | --- | --- | --- | --- | --- | --- | --- | --- | --- | --- | --- | --- | --- | --- | --- | --- | --- | --- | --- | --- | --- | --- | --- | --- | --- | --- | --- | --- | --- | --- | --- | --- | --- | --- | --- | --- | --- | --- | --- | --- | --- | --- | --- | --- | --- | --- | --- | --- | --- | --- | --- | --- | --- | --- | --- | --- | --- | --- | --- | --- | --- | --- | --- | --- | --- | --- | --- | --- | --- | --- | --- | --- | --- | --- | --- | --- | --- | --- | --- | --- | --- | --- | --- | --- | --- | --- | --- | --- | --- | --- | --- | --- | --- | --- | --- | --- | --- | --- | --- | --- | --- | --- | --- | --- | --- | --- | --- | --- | --- | --- | --- | --- | --- | --- | --- | --- | --- | --- | --- | --- | --- | --- | --- | --- | --- | --- | --- | --- | --- | --- | --- | --- | --- | --- | --- | --- | --- | --- | --- | --- | --- | --- | --- | --- | --- | --- | --- | --- | --- | --- | --- | --- | --- | --- | --- | --- | --- | --- | --- | --- | --- | --- | --- | --- | --- | --- | --- | --- | --- | --- | --- | --- | --- | --- | --- | --- | --- | --- | --- | --- | --- | --- | --- | --- | --- | --- | --- | --- | --- | --- | --- | --- | --- | --- | --- | --- | --- | --- | --- | --- | --- | --- | --- | --- | --- | --- | --- | --- | --- | --- | --- | --- | --- | --- | --- | --- | --- | --- | --- | --- | --- | --- | --- | --- | --- | --- | --- | --- | --- | --- | --- | --- | --- | --- | --- | --- | --- | --- | --- | --- | --- | --- | --- | --- | --- | --- | --- | --- | --- | --- | --- | --- | --- | --- | --- | --- | --- | --- | --- | --- | --- | --- | --- | --- | --- | --- | --- | --- | --- | --- | --- | --- | --- | --- | --- | --- | --- | --- | --- | --- | --- | --- | --- | --- | --- | --- | --- | --- | --- | --- | --- | --- | --- | --- | --- | --- | --- | --- | --- | --- | --- | --- | --- | --- | --- | --- | --- | --- | --- | --- | --- | --- | --- | --- | --- | --- | --- | --- | --- | --- | --- | --- | --- | --- | --- | --- | --- | --- | --- | --- | --- | --- | --- | --- | --- | --- | --- | --- | --- | --- | --- | --- | --- | --- | --- | --- | --- | --- | --- | --- | --- | --- | --- | --- | --- | --- | --- | --- | --- | --- | --- | --- | --- | --- | --- | --- | --- | --- | --- | --- | --- | --- | --- | --- | --- | --- | --- | --- | --- | --- | --- | --- | --- | --- | --- | --- | --- | --- | --- | --- | --- | --- | --- | --- | --- | --- | --- | --- | --- | --- | --- | --- | --- | --- | --- | --- | --- | --- | --- | --- | --- | --- | --- | --- | --- | --- | --- | --- | --- | --- | --- | --- | --- | --- | --- | --- | --- | --- | --- | --- | --- | --- | --- | --- | --- | --- | --- | --- | --- | --- | --- | --- | --- | --- | --- | --- | --- | --- | --- | --- | --- | --- | --- | --- | --- | --- | --- | --- | --- | --- | --- | --- | --- | --- | --- | --- | --- | --- | --- | --- | --- | --- | --- | --- | --- | --- | --- | --- | --- | --- | --- | --- | --- | --- | --- | --- | --- | --- | --- | --- | --- | --- | --- | --- | --- | --- | --- | --- | --- | --- | --- | --- | --- | --- | --- | --- | --- | --- | --- | --- | --- | --- | --- | --- | --- | --- | --- | --- | --- | --- | --- | --- | --- | --- | --- | --- | --- | --- | --- | --- | --- | --- | --- | --- | --- | --- | --- | --- | --- | --- | --- | --- | --- | --- | --- | --- | --- | --- | --- | --- | --- | --- | --- | --- | --- | --- | --- | --- | --- | --- | --- | --- | --- | --- | --- | --- | --- | --- | --- | --- | --- | --- | --- | --- | --- | --- | --- | --- | --- | --- | --- | --- | --- | --- | --- | --- | --- | --- | --- | --- | --- | --- | --- | --- | --- | --- | --- | --- | --- | --- | --- | --- | --- | --- | --- | --- | --- | --- | --- | --- | --- | --- | --- | --- | --- | --- | --- | --- | --- | --- | --- | --- | --- | --- | --- | --- | --- | --- | --- | --- | --- | --- | --- | --- | --- | --- | --- | --- | --- | --- | --- | --- | --- | --- | --- | --- | --- | --- | --- | --- | --- | --- | --- | --- | --- | --- | --- | --- | --- | --- | --- | --- | --- | --- | --- | --- | --- | --- | --- | --- | --- | --- | --- | --- | --- | --- | --- | --- | --- | --- | --- | --- | --- | --- | --- | --- | --- | --- | --- | --- | --- | --- | --- | --- | --- | --- | --- | --- | --- | --- | --- | --- | --- | --- | --- | --- | --- | --- | --- | --- | --- | --- | --- | --- | --- | --- | --- | --- | --- | --- | --- | --- | --- | --- | --- | --- | --- | --- | --- | --- | --- | --- | --- | --- | --- | --- | --- | --- | --- | --- | --- | --- | --- | --- | --- | --- | --- | --- | --- | --- | --- | --- | --- | --- | --- | --- | --- | --- | --- | --- | --- | --- | --- | --- | --- | --- | --- | --- | --- | --- | --- | --- | --- | --- | --- | --- | --- | --- | --- | --- | --- | --- | --- | --- | --- | --- | --- | --- | --- | --- | --- | --- | --- | --- | --- | --- | --- | --- | --- | --- | --- | --- | --- | --- | --- | --- | --- | --- | --- | --- | --- | --- | --- | --- | --- | --- | --- | --- | --- | --- | --- | --- | --- | --- | --- | --- | --- | --- | --- | --- | --- | --- | --- | --- | --- | --- | --- | --- | --- | --- | --- | --- | --- | --- | --- | --- | --- | --- | --- | --- | --- | --- | --- | --- | --- | --- | --- | --- | --- | --- | --- | --- | --- | --- | --- | --- | --- | --- | --- | --- | --- | --- | --- | --- | --- | --- | --- | --- | --- | --- | --- | --- | --- | --- | --- | --- | --- | --- | --- | --- | --- | --- | --- | --- | --- | --- | --- | --- | --- | --- | --- | --- | --- | --- | --- | --- | --- | --- | --- | --- | --- | --- | --- | --- | --- | --- | --- | --- | --- | --- | --- | --- | --- | --- | --- | --- | --- | --- | --- | --- | --- | --- | --- | --- | --- | --- | --- | --- | --- | --- | --- | --- | --- | --- | --- | --- | --- | --- | --- | --- | --- | --- | --- | --- | --- | --- | --- | --- | --- | --- | --- | --- | --- | --- | --- | --- | --- | --- | --- | --- | --- | --- | --- | --- | --- | --- | --- | --- | --- | --- | --- | --- | --- | --- | --- | --- | --- | --- | --- | --- | --- | --- | --- | --- | --- | --- | --- | --- | --- | --- | --- | --- | --- | --- | --- | --- | --- | --- | --- | --- | --- | --- | --- | --- | --- | --- | --- | --- | --- | --- | --- | --- | --- | --- | --- | --- | --- | --- | --- | --- | --- | --- | --- | --- | --- | --- | --- | --- | --- | --- | --- | --- | --- | --- | --- | --- | --- | --- | --- | --- | --- | --- | --- | --- | --- | --- | --- | --- | --- | --- | --- | --- | --- | --- | --- | --- | --- | --- | --- | --- | --- | --- | --- | --- | --- | --- | --- | --- | --- | --- | --- | --- | --- | --- | --- | --- | --- | --- | --- | --- | --- | --- | --- | --- | --- | --- | --- | --- | --- | --- | --- | --- | --- | --- | --- | --- | --- | --- | --- | --- | --- | --- | --- | --- | --- | --- | --- | --- | --- | --- | --- | --- | --- | --- | --- | --- | --- | --- | --- | --- | --- | --- | --- | --- | --- | --- | --- | --- | --- | --- | --- | --- | --- | --- | --- | --- | --- | --- | --- | --- | --- | --- | --- | --- | --- | --- | --- | --- | --- | --- | --- | --- | --- | --- | --- | --- | --- | --- | --- | --- | --- | --- | --- | --- | --- | --- | --- | --- | --- | --- | --- | --- | --- | --- | --- | --- | --- | --- | --- | --- | --- | --- | --- | --- | --- | --- | --- | --- | --- | --- | --- | --- | --- | --- | --- | --- | --- | --- | --- | --- | --- | --- | --- | --- | --- | --- | --- | --- | --- | --- | --- | --- | --- | --- | --- | --- | --- | --- | --- | --- | --- | --- | --- | --- | --- | --- | --- | --- | --- | --- | --- | --- | --- | --- | --- | --- | --- | --- | --- | --- | --- | --- | --- | --- | --- | --- | --- | --- | --- | --- | --- | --- | --- | --- | --- | --- | --- | --- | --- | --- | --- | --- | --- | --- | --- | --- | --- | --- | --- | --- | --- | --- | --- | --- | --- | --- | --- | --- | --- | --- | --- | --- | --- | --- | --- | --- | --- | --- | --- | --- | --- | --- | --- | --- | --- | --- | --- | --- | --- | --- | --- | --- | --- | --- | --- | --- | --- | --- | --- | --- | --- | --- | --- | --- | --- | --- | --- | --- | --- | --- | --- | --- | --- | --- | --- | --- | --- | --- | --- | --- | --- | --- | --- | --- | --- | --- | --- | --- | --- | --- | --- | --- | --- | --- | --- | --- | --- | --- | --- | --- | --- | --- | --- | --- | --- | --- | --- | --- | --- | --- | --- | --- | --- | --- | --- | --- | --- | --- | --- | --- | --- | --- | --- | --- | --- | --- | --- | --- | --- | --- | --- | --- | --- | --- | --- | --- | --- | --- | --- | --- | --- | --- | --- | --- | --- | --- | --- | --- | --- | --- | --- | --- | --- | --- | --- | --- | --- | --- | --- | --- | --- | --- | --- | --- | --- | --- | --- | --- | --- | --- | --- | --- | --- | --- | --- | --- | --- | --- | --- | --- | --- | --- | --- | --- | --- | --- | --- | --- | --- | --- | --- | --- | --- | --- | --- | --- | --- | --- | --- | --- | --- | --- | --- | --- | --- | --- | --- | --- | --- | --- | --- | --- | --- | --- | --- | --- | --- | --- | --- | --- | --- | --- | --- | --- | --- | --- | --- | --- | --- | --- | --- | --- | --- | --- | --- | --- | --- | --- | --- | --- | --- | --- | --- | --- | --- | --- | --- | --- | --- | --- | --- | --- | --- | --- | --- | --- | --- | --- | --- | --- | --- | --- | --- | --- | --- | --- | --- | --- | --- | --- | --- | --- | --- | --- | --- | --- | --- | --- | --- | --- | --- | --- | --- | --- | --- | --- | --- | --- | --- | --- | --- | --- | --- | --- | --- | --- | --- | --- | --- | --- | --- | --- | --- | --- | --- | --- | --- | --- | --- | --- | --- | --- | --- | --- | --- | --- | --- | --- | --- | --- | --- | --- | --- | --- | --- | --- | --- | --- | --- | --- | --- | --- | --- | --- | --- | --- | --- | --- | --- | --- | --- | --- | --- | --- | --- | --- | --- | --- | --- | --- | --- | --- | --- | --- | --- | --- | --- | --- | --- | --- | --- | --- | --- | --- | --- | --- | --- | --- | --- | --- | --- | --- | --- | --- | --- | --- | --- | --- | --- | --- | --- | --- | --- | --- | --- | --- | --- | --- | --- | --- | --- | --- | --- | --- | --- | --- | --- | --- | --- | --- | --- | --- | --- | --- | --- | --- | --- | --- | --- | --- | --- | --- | --- | --- | --- | --- | --- | --- | --- | --- | --- | --- | --- | --- | --- | --- | --- | --- | --- | --- | --- | --- | --- | --- | --- | --- | --- | --- | --- | --- | --- | --- | --- | --- | --- | --- | --- | --- | --- | --- | --- | --- | --- | --- | --- | --- | --- | --- | --- | --- | --- | --- | --- | --- | --- | --- | --- | --- | --- | --- | --- | --- | --- | --- | --- | --- | --- | --- | --- | --- | --- | --- | --- | --- | --- | --- | --- | --- | --- | --- | --- | --- | --- | --- | --- | --- | --- | --- | --- | --- | --- | --- | --- | --- | --- | --- | --- | --- | --- | --- | --- | --- | --- | --- | --- | --- | --- | --- | --- | --- | --- | --- | --- | --- | --- | --- | --- | --- | --- | --- | --- | --- | --- | --- | --- | --- | --- | --- | --- | --- | --- | --- | --- | --- | --- | --- | --- | --- | --- | --- | --- | --- | --- | --- | --- | --- | --- | --- | --- | --- | --- | --- | --- | --- | --- | --- | --- | --- | --- | --- | --- | --- | --- | --- | --- | --- | --- | --- | --- | --- | --- | --- | --- | --- | --- | --- | --- | --- | --- | --- | --- | --- | --- | --- | --- | --- | --- | --- | --- | --- | --- | --- | --- | --- | --- | --- | --- | --- | --- | --- | --- |

**Supplementary Table 3. Included ELA-subtypes.**

| **ELA-subtype** | **Classification** | **Studies** |
| --- | --- | --- |
| Drug exposure | prenatal | Grewen, 2015; Li, 2019; Radhakrishnan, 2020; Salzwedel, 2015 |
| Family adversity | postnatal, social | Herringa, 2016 |
| Harsh parenting | postnatal, social | La Buissonniere-Ariza, 2019 |
| Institutionalization | postnatal, social | Gee, 2013; Silvers, 2016 |
| Interpersonal violence | postnatal, social | Buchweitz, 2019 |
| Maltreatment | postnatal, social | Birn, 2014; Cancel, 2017; Cisler, 2017; Dean, 2014; Duque-Alarcon, 2019; Fan, 2014; 2015; Fonzo, 2013; Herringa, 2013; Holz, 2015; Jedd, 2015; Krause-Utz, 2014; Maier, 2020; Peverill, 2019; Quidé, 2020; van der Werff, 2013;2013 |
| Maternal psychiatry (depression/anxiety) | prenatal | Posner, 2016; Scheinost, 2016 |
| Maternal hostility | postnatal, social | Kopala-Sibley, 2020 |
| Neighborhood disadvantage | Postnatal, socioeconomic | Gard, 2020 |
| Physical abuse | postnatal, social | Kraynak, 2019 |
| Poverty | Postnatal, socioeconomic | Barch, 2016; Turesky, 2019 |
| Social-economic status | Postnatal, socioeconomic | Dégeilb, 2020; Hanson, 2019; Javanbakht, 2015 |
| Stressful live events | postnatal | Hanford, 2019; Park, 2018 |
| Threat-related early life stress | postnatal, social | Kaiser, 2018 |
| Trauma | postnatal, social | Fortenbaugh, 2017; Keding, 2016; Pagliaccio, 2015; Thomason, 2015; van Rooij, 2020; Wolf, 2016 |
| Traumatic stress | postnatal, social | Colich, 2017 |
| Verbal abuse | postnatal, social | Lee, 2015 |
| Violence exposure | postnatal, social | Dark, 2020; van Rooij, 2020; Zielinski, 2018 |

**Supplementary Table 4. Included studies per analysis.**

| **Analysis** | **Included studies** |
| --- | --- |
| All studies | Barch, 2016 (1); Birn, 2014 (2); Buchweitz, 2019 (3); Cancel (4); Cisler, 2017 (5); Colich, 2017 (6); Dark, 2020 (7); Dean, 2014 (8); Dégeilh, 2020 (9); Duque-Alarcón, 2019 (10); Fan, 2014 (11); Fan, 2015 (12); Fonzo, 2013 (13); Fortenbaugh, 2017 (14); Gard, 2020 (15); Gee, 2013 (16); Grewen, 2015 (17); Hanford, 2019 (18); Hanson, 2019 (19); Herringa, 2013 (20); Herringa, 2016 (21); Holz, 2015 (22); Javanbakht, 2015 (23); Jedd, 2015 (24); Kaiser, 2018 (25); Keding, 2016 (26); Kopala-Sibley, 2020 (27); Krause-Utz, 2014 (28); Kraynak, 2019 (29); La Buissonniere-Ariza, 2019 (30); Lee, 2015 (31); Li, 2019 (32); Maier, 2020 (33); Pagliaccio, 2015 (34); Park, 2018 (35); Peverill, 2019 (36); Posner, 2016 (37); Quidé, 2020 (38); Radhakrishnan, 2020 (39); Salzwedel, 2015 (40); Scheinost, 2016 (41); Silvers, 2016 (42); Thomason, 2015 (43); Turesky, 2019 (44); van der Werff, 2013 (45); van der Werff, 2013 (46); van Rooij, 2020 (47); Wolf, 2016 (48); Zielinski, 2018 (49) |
| Decrease in connectivity | Barch, 2016 (1); Birn, 2014 (2); Cancel (4); Cisler, 2017 (5); Colich, 2017 (6); Dark, 2020 (7); Dégeilh, 2020 (9); Duque-Alarcón, 2019 (10); Fan, 2014 (11); Fonzo, 2013 (13); Fortenbaugh, 2017 (14); Gee, 2013 (16); Hanford, 2019 (18); Hanson, 2019 (19); Herringa, 2013 (20); Holz, 2015 (22); Jedd, 2015 (24); Kaiser, 2018 (25); Kopala-Sibley, 2020 (27); Kraynak, 2019 (29); La Buissonniere-Ariza, 2019 (30); Lee, 2015 (31); Park, 2018 (35); Peverill, 2019 (36); Posner, 2016 (37); Radhakrishnan, 2020 (39); Scheinost, 2016 (41); Thomason, 2015 (43); van der Werff, 2013 (45); van der Werff, 2013 (46); Wolf, 2016 (48) |
| Increase in connectivity | Barch, 2016 (1); Birn, 2014 (2); Buchweitz, 2019 (3); Dean, 2014 (8); Dégeilh, 2020 (9); Fan, 2015 (12); Fonzo, 2013 (13); Fortenbaugh, 2017 (14); Gard, 2020 (15); Grewen, 2015 (17); Hanford, 2019 (18); Hanson, 2019 (19); Herringa, 2013 (20); Herringa, 2016 (21); Holz, 2015 (22); Javanbakht, 2015 (23); Kaiser, 2018 (25); Keding, 2016 (26); Krause-Utz, 2014 (28); La Buissonniere-Ariza, 2019 (30); Li, 2019 (32); Maier, 2020 (33); Pagliaccio, 2015 (34); Quidé, 2020 (38); Radhakrishnan, 2020 (39); Salzwedel, 2015 (40); Silvers, 2016 (42); Thomason, 2015 (43); Turesky, 2019 (44); van der Werff, 2013 (45); van der Werff, 2013 (46); van Rooij, 2020 (47); Zielinski, 2018 (49) |
| Right hemisphere | Barch, 2016 (1); Birn, 2014 (2); Buchweitz, 2019 (3); Colich, 2017 (6); Dégeilh, 2020 (9); Duque-Alarcón, 2019 (10); Fan, 2014 (11); Fan, 2015 (12); Fortenbaugh, 2017 (14); Gard, 2020 (15); Gee, 2013 (16); Hanford, 2019 (18); Hanson, 2019 (19); Herringa, 2013 (20); Herringa, 2016 (21); Jedd, 2015 (24); Kaiser, 2018 (25); Keding, 2016 (26); Kopala-Sibley, 2020 (27); Krause-Utz, 2014 (28); Kraynak, 2019 (29); Lee, 2015 (31); Li, 2019 (32); Maier, 2020 (33); Park, 2018 (35); Posner, 2016 (37); Quidé, 2020 (38); Radhakrishnan, 2020 (39); Silvers, 2016 (42); Thomason, 2015 (43); Turesky, 2019 (44); van der Werff, 2013 (45); van der Werff, 2013 (46) |
| Left hemisphere | Barch, 2016 (1); Birn, 2014 (2); Buchweitz, 2019 (3); Cisler, 2017 (5); Colich, 2017 (6); Dark, 2020 (7); Dégeilh, 2020 (9); Fan, 2015 (12); Fonzo, 2013 (13); Fortenbaugh, 2017 (14); Gard, 2020 (15); Grewen, 2015 (17); Hanford, 2019 (18); Hanson, 2019 (19); Herringa, 2013 (20); Holz, 2015 (22); Javanbakht, 2015 (23); Jedd, 2015 (24); Kaiser, 2018 (25); Kopala-Sibley, 2020 (27); Kraynak, 2019 (29); La Buissonniere-Ariza, 2019 (30); Li, 2019 (32); Pagliaccio, 2015 (34); Park, 2018 (35); Peverill, 2019 (36); Posner, 2016 (37); Radhakrishnan, 2020 (39); Salzwedel, 2015 (40); Scheinost, 2016 (41); Silvers, 2016 (42); Thomason, 2015 (43); Turesky, 2019 (44); van der Werff, 2013 (46); van Rooij, 2020 (47); Wolf, 2016 (48); Zielinski, 2018 (49) |
| Resting-state | Barch, 2016 (1); Birn, 2014 (2); Cisler, 2017 (5); Dark, 2020 (7); Dean, 2014 (8); Dégeilh, 2020 (9); Duque-Alarcón, 2019 (10); Fan, 2014 (11); Fortenbaugh, 2017 (14); Grewen, 2015 (17); Hanson, 2019 (19); Herringa, 2013 (20); Kaiser, 2018 (25); Krause-Utz, 2014 (28); Kraynak, 2019 (29); Li, 2019 (32); Pagliaccio, 2015 (34); Park, 2018 (35); Posner, 2016 (37); Radhakrishnan, 2020 (39); Salzwedel, 2015 (40); Scheinost, 2016 (41); Thomason, 2015 (43); Turesky, 2019 (44); van der Werff, 2013 (45); van der Werff, 2013 (46); Zielinski, 2018 (49) |
| Task-based | Buchweitz, 2019 (3); Cancel (4); Colich, 2017 (6); Fan, 2015 (12); Fonzo, 2013 (13); Gard, 2020 (15); Gee, 2013 (16); Hanford, 2019 (18); Herringa, 2016 (21); Holz, 2015 (22); Javanbakht, 2015 (23); Jedd, 2015 (24); Keding, 2016 (26); Kopala-Sibley, 2020 (27); La Buissonniere-Ariza, 2019 (30); Lee, 2015 (31); Maier, 2020 (33); Peverill, 2019 (36); Quidé, 2020 (38); Silvers, 2016 (42); van Rooij, 2020 (47); Wolf, 2016 (48) |
| Emotion processing | Buchweitz, 2019 (3); Cancel (4); Colich, 2017 (6); Fonzo, 2013 (13); Gard, 2020 (15); Gee, 2013 (16); Hanford, 2019 (18); Herringa, 2016 (21); Holz, 2015 (22); Javanbakht, 2015 (23); Jedd, 2015 (24); Keding, 2016 (26); Kopala-Sibley, 2020 (27); La Buissonniere-Ariza, 2019 (30); Lee, 2015 (31); Maier, 2020 (33); Peverill, 2019 (36); Quidé, 2020 (38); Silvers, 2016 (42); van Rooij, 2020 (47); Wolf, 2016 (48) |
| Adults | Birn, 2014 (2); Cancel (4); Dark, 2020 (7); Dean, 2014 (8); Duque-Alarcón, 2019 (10); Fan, 2014 (11); Fan, 2015 (12); Fonzo, 2013 (13); Fortenbaugh, 2017 (14); Gard, 2020 (15); Herringa, 2013 (20); Herringa, 2016 (21); Holz, 2015 (22); Javanbakht, 2015 (23); Jedd, 2015 (24); Kaiser, 2018 (25); Krause-Utz, 2014 (28); Kraynak, 2019 (29); Maier, 2020 (33); Quidé, 2020 (38); van der Werff, 2013 (45); van der Werff, 2013 (46); Zielinski, 2018 (49) |
| Children | Barch, 2016 (1); Buchweitz, 2019 (3); Cisler, 2017 (5); Colich, 2017 (6); Dégeilh, 2020 (9); Gee, 2013 (16); Grewen, 2015 (17); Hanford, 2019 (18); Hanson, 2019 (19); Keding, 2016 (26); Kopala-Sibley, 2020 (27); La Buissonniere-Ariza, 2019 (30); Lee, 2015 (31); Li, 2019 (32); Pagliaccio, 2015 (34); Park, 2018 (35); Peverill, 2019 (36); Posner, 2016 (37); Radhakrishnan, 2020 (39); Salzwedel, 2015 (40); Scheinost, 2016 (41); Silvers, 2016 (42); Thomason, 2015 (43); Turesky, 2019 (44); van Rooij, 2020 (47); Wolf, 2016 (48) |
| Healthy | Barch, 2016 (1); Buchweitz, 2019 (3); Colich, 2017 (6); Dark, 2020 (7); Dégeilh, 2020 (9); Fan, 2014 (11); Fan, 2015 (12); Gard, 2020 (15); Gee, 2013 (16); Grewen, 2015 (17); Hanford, 2019 (18); Hanson, 2019 (19); Herringa, 2013 (20); Holz, 2015 (22); Javanbakht, 2015 (23); Jedd, 2015 (24); Kopala-Sibley, 2020 (27); Kraynak, 2019 (29); La Buissonniere-Ariza, 2019 (30); Lee, 2015 (31); Li, 2019 (32); Maier, 2020 (33); Park, 2018 (35); Peverill, 2019 (36); Posner, 2016 (37); Radhakrishnan, 2020 (39); Salzwedel, 2015 (40); Scheinost, 2016 (41); Silvers, 2016 (42); Thomason, 2015 (43); Turesky, 2019 (44) |
| Social ACE | Birn, 2014 (2); Buchweitz, 2019 (3); Cancel (4); Cisler, 2017 (5); Colich, 2017 (6); Dark, 2020 (7); Dean, 2014 (8); Duque-Alarcón, 2019 (10); Fan, 2014 (11); Fan, 2015 (12); Fonzo, 2013 (13); Fortenbaugh, 2017 (14); Gee, 2013 (16); Herringa, 2013 (20); Herringa, 2016 (21); Holz, 2015 (22); Jedd, 2015 (24); Kopala-Sibley, 2020 (27); Kaiser, 2018 (25); Keding, 2016 (26); Krause-Utz, 2014 (28); Kraynak, 2019 (29); La Buissonniere-Ariza, 2019 (30); Lee, 2015 (31); Maier, 2020 (33); Pagliaccio, 2015 (34); Peverill, 2019 (36); Quidé, 2020 (38); Silvers, 2016 (42); Thomason, 2015 (43); van der Werff, 2013 (45); van der Werff, 2013 (46); van Rooij, 2020 (47); Wolf, 2016 (48); Zielinski, 2018 (49) |
| Postnatal | Barch, 2016 (1); Birn, 2014 (2); Buchweitz, 2019 (3); Cancel (4); Cisler, 2017 (5); Colich, 2017 (6); Dark, 2020 (7); Dean, 2014 (8); Dégeilh, 2020 (9); Duque-Alarcón, 2019 (10); Fan, 2014 (11); Fan, 2015 (12); Fonzo, 2013 (13); Fortenbaugh, 2017 (14); Gard, 2020 (15); Gee, 2013 (16); Hanford, 2019 (18); Hanson, 2019 (19); Herringa, 2013 (20); Herringa, 2016 (21); Holz, 2015 (22); Javanbakht, 2015 (23); Jedd, 2015 (24); Kaiser, 2018 (25); Keding, 2016 (26); Kopala-Sibley, 2020 (27); Krause-Utz, 2014 (28); Kraynak, 2019 (29); La Buissonniere-Ariza, 2019 (30); Lee, 2015 (31); Maier, 2020 (33); Pagliaccio, 2015 (34); Park, 2018 (35); Peverill, 2019 (36); Quidé, 2020 (38); Silvers, 2016 (42); Thomason, 2015 (43); Turesky, 2019 (44); van der Werff, 2013 (45); van der Werff, 2013 (46); van Rooij, 2020 (47); Wolf, 2016 (48); Zielinski, 2018 (49) |
| Retrospective assessment | Birn, 2014 (2); Buchweitz, 2019 (3); Cancel (4); Cisler, 2017 (5); Colich, 2017 (6); Dean, 2014 (8); Duque-Alarcón, 2019 (10); Fan, 2014 (11); Fan, 2015 (12); Fonzo, 2013 (13); Fortenbaugh, 2017 (14); Gee, 2013 (16); Hanford, 2019 (18); Herringa, 2013 (20); Holz, 2015 (22); Javanbakht, 2015 (23); Kaiser, 2018 (25); Keding, 2016 (26); Krause-Utz, 2014 (28); Kraynak, 2019 (29); Lee, 2015 (31); Maier, 2020 (33); Pagliaccio, 2015 (34); Park, 2018 (35); Peverill, 2019 (36); Quidé, 2020 (38); Scheinost, 2016 (41); Silvers, 2016 (42); Thomason, 2015 (43); van der Werff, 2013 (45); van der Werff, 2013 (46); van Rooij, 2020 (47); Wolf, 2016 (48); Zielinski, 2018 (49) |
| Subjective self-report by participants | Birn, 2014 (2); Buchweitz, 2019 (3); Cancel (4); Cisler, 2017 (5); Dark, 2020 (7); Dean, 2014 (8); Duque-Alarcón, 2019 (10); Fan, 2014 (11); Fan, 2015 (12); Fonzo, 2013 (13); Fortenbaugh, 2017 (14); Hanford, 2019 (18); Herringa, 2013 (20); Holz, 2015 (22); Kaiser, 2018 (25); Keding, 2016 (26); Krause-Utz, 2014 (28); Kraynak, 2019 (29); Lee, 2015 (31); Maier, 2020 (33); Pagliaccio, 2015 (34); Peverill, 2019 (36); Quidé, 2020 (38); Thomason, 2015 (43); van der Werff, 2013 (45); van der Werff, 2013 (46); van Rooij, 2020 (47); Wolf, 2016 (48); Zielinski, 2018 (49) |

ACE, adverse childhood experiences.

**Supplementary Table 5.**

| **ALE analysis** | ***N*** | **#Experi-ments** | **#Foci** | **Cluster** | **Volume (mm^3^)** | **Brain region** | ***X*** | ***Y*** | ***Z*** | **ALE value** | **Z score** | **Studies contributing to cluster** |
| --- | --- | --- | --- | --- | --- | --- | --- | --- | --- | --- | --- | --- |
| Decrease in connectivity (all studies) | 2175 | 30 | 131 | 1 | 1344 | L ACC  L ACC | 2  -6 | 42  38 | 14  6 | 0.0146  0.0138 | 3.87  3.76 | Cisler, 2017; Fan, 2014; Kraynak, 2020; Lee, 2015; Radhakrishnan, 2020; Thomason, 2015; Wolf, 2016 |
| Decrease in connectivity (mean age > 10 years)* | 2127 | 28 | 126 | 1 | 1384 | L ACC | 2 | 42 | 14 | 0.0146 | 3.89 | Cisler, 2017; Fan, 2014; Kraynak, 2020; Lee, 2015; Thomason, 2015; Wolf, 2016 |

*without Radhakrishnan, 2020 (39); Scheinost, 2016 (41).

L = left; *X, Y, Z* coordinates in MNI152 space. ACC, anterior cingulate cortex; ALE, activation likelihood estimation.

**Supplemental References**

1. Barch D, Pagliaccio D, Belden A, Harms MP, Gaffrey M, Sylvester CM, et al. Effect of hippocampal and amygdala connectivity on the relationship between preschool poverty and school-age depression. Am J Psychiatry. 2016 Jun;173(6):625–34.

2. Birn RM, Patriat R, Phillips ML, Germain A, Herringa RJ. Childhood maltreatment and combat posttraumatic stress differentially predict fear-related fronto-subcortical connectivity. Depress Anxiety. 2014 Oct;31(10):880–92.

3. Buchweitz A, de Azeredo LA, Sanvicente-Vieira B, Cara VM, Esper NB, Soder RB, et al. Violence and Latin-American preadolescents: A study of social brain function and cortisol levels. Dev Sci. 2019 Sep;22(5, SI):e12799.

4. Cancel A, Comte M, Boutet C, Schneider FC, Rousseau P-F, Boukezzi S, et al. Childhood trauma and emotional processing circuits in schizophrenia: A functional connectivity study. Schizophr Res. 2017 Jun;184:69–72.

5. Cisler JM. Childhood trauma and functional connectivity between amygdala and medial prefrontal cortex: A dynamic functional connectivity and large-scale network perspective. Front Syst Neurosci. 2017 May;11:29.

6. Colich NL, Williams ES, Ho TC, King LS, Humphreys KL, Price AN, et al. The association between early life stress and prefrontal cortex activation during implicit emotion regulation is moderated by sex in early adolescence. Dev Psychopathol. 2017 Dec;29(5):1851–64.

7. Dark H, Harnett N, Goodman A, Wheelock M, Mrug S, Schuster M, et al. Violence exposure, affective stule, and stress-induced changes in resting state functional connectivity. Cogn Affect Behav Neurosci. 2020;20:1261–77.

8. Dean AC, Kohno M, Hellemann G, London ED. Childhood maltreatment and amygdala connectivity in methamphetamine dependence: a pilot study. Brain Behav. 2014;4(6):867–76.

9. Dégeilh F, Dégeilh F, Beauchamp MH, Beauchamp MH, Leblanc É, Daneault V, et al. Socioeconomic status in infancy and the developing brain: Functional connectivity of the hippocampus and amygdala. Dev Neurosci. 2020;41(5–6):327–40.

10. Duque-Alarcón X, Alcalá-Lozano R, González-Olvera JJ, Garza-Villarreal EA, Pellicer F, X. D-A, et al. Effects of childhood maltreatment on social cognition and brain functional connectivity in borderline personality disorder patients. Front Psychiatry. 2019 Mar;10(MAR).

11. Fan Y, Herrera-Melendez AL, Pestke K, Feeser M, Aust S, Otte C, et al. Early life stress modulates amygdala-prefrontal functional connectivity: implications for oxytocin effects. Hum Brain Mapp. 2014 Oct;35(10):5328–39.

12. Fan Y, Pestke K, Feeser M, Aust S, Pruessner JC, Boker H, et al. Amygdala-hippocampal connectivity changes during acute psychosocial stress: joint effect of early life stress and oxytocin. Neuropsychopharmacology. 2015 Nov;40(12):2736–44.

13. Fonzo GA, Flagan TM, Sullivan S, Allard CB, Grimes EM, Simmons AN, et al. Neural functional and structural correlates of childhood maltreatment in women with intimate-partner violence-related posttraumatic stress disorder. Psychiatry Res - Neuroimaging. 2013 Feb;211(2):93–103.

14. Fortenbaugh FC, Corbo V, Poole V, McGlinchey R, Milberg W, Salat D, et al. Interpersonal early-life trauma alters amygdala connectivity and sustained attention performance. Brain Behav. 2017 May;7(5):e00684.

15. Gard AM, Maxwell AM, Shaw DS, Mitchell C, Brooks-Gunn J, McLanahan SS, et al. Beyond family-level adversities: Exploring the developmental timing of neighborhood disadvantage effects on the brain. Dev Sci. 2020 May;e12985.

16. Gee DG, Gabard-Durnam LJ, Flannery J, Goff B, Humphreys KL, Telzer EH, et al. Early developmental emergence of human amygdala-prefrontal connectivity after maternal deprivation. Proc Natl Acad Sci U S A. 2013;110(39):15638–43.

17. Grewen K, Salzwedel AP, Gao W. Functional connectivity disruption in neonates with prenatal marijuana exposure. Front Hum Neurosci. 2015;9:601.

18. Hanford LC, Eckstrand K, Manelis A, Hafeman DM, Merranko J, Ladouceur CD, et al. The impact of familial risk and early life adversity on emotion and reward processing networks in youth at-risk for bipolar disorder. PLoS One. 2019;14(12):e0226135.

19. Hanson JL, Albert WD, Skinner AT, Shen SH, Dodge KA, Lansford JE. Resting state coupling between the amygdala and ventromedial prefrontal cortex is related to household income in childhood and indexes future psychological vulnerability to stress. Dev Psychopathol. 2019;31(3):1053–66.

20. Herringa RJ, Birn RM, Ruttle PL, Burghy CA, Stodola DE, Davidson RJ, et al. Childhood maltreatment is associated with altered fear circuitry and increased internalizing symptoms by late adolescence. Proc Natl Acad Sci U S A. 2013 Nov;110(47):19119–24.

21. Herringa R, Burghy C, Stodola D, Fox M, Davidson R, Essex M. Enhanced prefrontal-amygdala connectivity following childhood adversity as a protective mechanism against internalizing in adolescence. Biol Psychiatry Cogn Neurosci Neuroimaging. 2016;1(4):326–34.

22. Holz N, Buchmann A, Boecker R, Blomeyer D, Baumeister S, Wolf I, et al. Role of FKBP5 in emotion processing: results on amygdala activity, connectivity and volume. Brain Struct Funct. 2015;220:1355–68.

23. Javanbakht A, King AP, Evans GW, Swain JE, Angstadt M, Phan KL, et al. Childhood poverty predicts adult amygdala and frontal activity and connectivity in response to emotional faces. Front Behav Neurosci. 2015;9:154.

24. Jedd K, Hunt RH, Cicchetti D, Hunt E, Cowell RA, Rogosch FA, et al. Long-term consequences of childhood maltreatment: Altered amygdala functional connectivity. Dev Psychopathol. 2015 Nov;27(4 Pt 2):1577–89.

25. Kaiser RH, Clegg R, Goer F, Pechtel P, Beltzer M, Vitaliano G, et al. Childhood stress, grown-up brain networks: corticolimbic correlates of threat-related early life stress and adult stress response. Psychol Med. 2018;48(7):1157–66.

26. Keding TJ, Herringa RJ. Paradoxical prefrontal-amygdala recruitment to angry and happy expressions in pediatric posttraumatic stress disorder. Neuropsychopharmacology. 2016 Nov;41(12):2903–12.

27. Kopala-Sibley DC, Cyr M, Finsaas MC, Orawe J, Huang A, Tottenham N, et al. Early childhood parenting predicts late childhood brain functional connectivity during emotion perception and reward processing. Child Dev. 2020 Jan;91(1):110–28.

28. Krause-Utz A, Veer I, Rombouts S, Bohus M, Schmahl C, Elzinga B. Amygdala and anterior cingulate resting-state functional connectivity in borderline personality disorder patients with a history of interpersonal trauma. Psychol Med. 2014;44:2889–901.

29. Kraynak TE, Marsland AL, Hanson JL, Gianaros PJ. Retrospectively reported childhood physical abuse, systemic inflammation, and resting corticolimbic connectivity in midlife adults. Brain Behav Immun. 2019;82:203–13.

30. La Buissonniere-Ariza V, Seguin JR, Nassim M, Boivin M, Pine DS, Lepore F, et al. Chronic harsh parenting and anxiety associations with fear circuitry function in healthy adolescents: A preliminary study. Biol Psychol. 2019 Jul;145:198–210.

31. Lee SW, Yoo JH, Kim KW, Lee J-S, Kim D, Park H, et al. Aberrant function of frontoamygdala circuits in adolescents with previous verbal abuse experiences. Neuropsychologia. 2015 Dec;79(A):76–85.

32. Li Z, Lei K, Coles C, Lynch M, Hu X. Longitudinal changes of amygdala functional connectivity in adolescents prenatally exposed to cocaine. Drug Alcohol Depend. 2019;200:50–8.

33. Maier A, Heinen-Ludwig L, Güntürkün O, Hurlemann R, Scheele D. Childhood maltreatment alters the neural processing of chemosensory stress signals. Front Psychiatry. 2020;11.

34. Pagliaccio D, Luby JL, Bogdan R, Agrawal A, Gaffrey MS, Belden AC, et al. Amygdala functional connectivity, HPA axis genetic variation, and life stress in children and relations to anxiety and emotion regulation. J Abnorm Psychol. 2015 Nov;124(4):817–33.

35. Park AT, Leonard JA, Saxler PK, Cyr AB, Gabrieli JDE, Mackey AP. Amygdala-medial prefrontal cortex connectivity relates to stress and mental health in early childhood. Soc Cogn Affect Neurosci. 2018 Apr;13(4):430–9.

36. Peverill M, Sheridan MA, Busso DS, McLaughlin KA. Atypical prefrontal-amygdala circuitry following childhood exposure to abuse: links with adolescent psychopathology. Child Maltreat. 2019 Nov;24(4, SI):411–23.

37. Posner J, Cha J, Roy AK, Peterson BS, Bansal R, Gustafsson HC, et al. Alterations in amygdala-prefrontal circuits in infants exposed to prenatal maternal depression. Transl Psychiatry. 2016 Nov;6(11):e935.

38. Quidé Y, Girshkin L, Watkeys OJ, Carr VJ, Green MJ. The relationship between cortisol reactivity and emotional brain function is differently moderated by childhood trauma, in bipolar disorder, schizophrenia and healthy individuals. Eur Arch Psychiatry Clin Neurosci [Internet]. 2020; Available from: http://dx.doi.org/10.1007/s00406-020-01190-3

39. Radhakrishnan R, Elsaid NMH, Sadhasivam S, Reher TA, Hines AC, Yoder KK, et al. Resting state functional MRI in infants with prenatal opioid exposure-a pilot study. Neuroradiology. 2020 Sep;

40. Salzwedel AP, Grewen KM, Vachet C, Gerig G, Lin W, Gao W. Prenatal drug exposure affects neonatal brain functional connectivity. J Neurosci. 2015 Apr;35(14):5860–9.

41. Scheinost D, Kwon SH, Lacadie C, Sze G, Sinha R, Constable RT, et al. Prenatal stress alters amygdala functional connectivity in preterm neonates. NeuroImage Clin. 2016;12:381–8.

42. Silvers JA, Lumian DS, Gabard-Durnam L, Gee DG, Goff B, Fareri DS, et al. Previous institutionalization is followed by broader amygdala-hippocampal-PFC network connectivity during aversive learning in human development. J Neurosci. 2016 Jun;36(24):6420–30.

43. Thomason M, Marusak H, Tocco M, Vila A, McGarragle O, Rosenberg D. Altered amygdala connectivity in urban youth exposed to trauma. Soc Cogn Affect Neurosci. 2015;10(11):1460–8.

44. Turesky T, Jensen S, Yu X, Kumar S, Wang Y, Sliva D, et al. The relationship between biological and psychosocial risk factors and resting-state functional connectivity in 2-month-old Bangladeshi infants: A feasibility and pilot study. Dev Sci. 2019;22(5):e12841.

45. van der Werff SJA, Pannekoek JN, Veer IM, van Tol M-J, Aleman A, Veltman DJ, et al. Resting-state functional connectivity in adults with childhood emotional maltreatment. Psychol Med. 2013 Sep;43(9):1825–36.

46. van der Werff SJA, Pannekoek JN, Veer IM, van Tol M-JM-J, Aleman A, Veltman DJ, et al. Resilience to childhood maltreatment is associated with increased resting-state functional connectivity of the salience network with the lingual gyrus. Child Abus Negl. 2013 Nov;37(11):1021–9.

47. van Rooij SJH, Smith RD, Stenson AF, Ely TD, Yang X, Tottenham N, et al. Increased activation of the fear neurocircuitry in children exposed to violence. Depress Anxiety. 2020;

48. Wolf RC, Herringa RJ. Prefrontal-amygdala dysregulation to threat in pediatric posttraumatic stress disorder. Neuropsychopharmacology. 2016 Feb;41(3):822–31.

49. Zielinski MJ, Privratsky AA, Smitherman S, Kilts CD, Herringa RJ, Cisler JM. Does development moderate the effect of early life assaultive violence on resting-state networks? An exploratory study. Psychiatry Res - Neuroimaging. 2018 Nov;281:69–77.
